# AI‐Augmented Hematological Signatures for Equitable Detection of Hereditary Hemolytic Anemia Carriers: A Global Systematic Review and Meta‐Analysis

**DOI:** 10.1155/humu/9405486

**Published:** 2026-06-27

**Authors:** Naif Taleb Ali, Radfan Saleh Abdullah, Mansour Abdulnabi H. Mehdi, Gamila Saleh Ali, Hana Mohsen Ali, Ali N. M. Gubran, Nazeh Mohammed Al-Abd

**Affiliations:** ^1^ Department of Health Sciences, Faculty of Medicine and Health Sciences, University of Science and Technology, Aden, Yemen, hust.edu.vn; ^2^ Department of Medical Laboratory, Radfan University College, University of Lahej, Alhouta, Yemen; ^3^ Department of Medicine, Faculty of Medicine and Health Sciences, University of Aden, Aden, Yemen, aden-univ.net

**Keywords:** artificial intelligence, complete blood count, diagnostic accuracy, hereditary hemolytic anemia, premarital screening, resource-limited settings, sickle cell disease, thalassemia

## Abstract

Artificial intelligence (AI) augmentation of routine hematological tests offers a promising strategy to improve hereditary hemolytic anemia (HHA) carrier detection in premarital screening, especially in resource‐limited settings. HHA in this review specifically encompasses *β*‐thalassemia, *α*‐thalassemia, sickle cell disease (including HbS and HbC variants), and other hemoglobinopathies with autosomal recessive inheritance patterns requiring carrier detection for prevention. These conditions share hemolytic phenotypes but differ in hematological signatures, necessitating separate subgroup analyses. This global systematic review and meta‐analysis evaluated the diagnostic accuracy, equity implications, and implementation challenges of AI‐augmented complete blood count (CBC), blood smear, and erythrocyte sedimentation rate (ESR) for HHA carrier identification. We systematically searched seven databases and included 85 studies (*n* = 133,498 participants, 23 countries). AI‐augmented screening achieved a pooled sensitivity of 92.8% (95% CI: 91.3%–94.1%) and specificity of 91.5% (89.7%–93.0%), representing a 12.3% sensitivity improvement over conventional interpretation (*p* < 0.001). However, significant geographic disparities were observed: sensitivity in Sub‐Saharan Africa was 86.5% compared with 94.8% in the Middle East (*p* < 0.001), partly due to algorithmic bias against African HbS/HbC variants and infrastructural barriers. Deep learning models achieved the highest sensitivity (95.1%), whereas explainable artificial intelligence (XAI) provided optimal specificity (94.3%). Integrating CBC with blood smear increased specificity by 5.5% at minimal additional cost. AI triage reduced confirmatory testing by 23.7%, saving $8.50 per individual. For equitable implementation, we recommend the following: (1) federated learning to include underrepresented genotypes, (2) WHO/CDC certification of affordable, offline‐capable edge AI devices, and (3) mandatory XAI compliance with bias audits. AI can transform HHA screening, but deliberate efforts are needed to avoid exacerbating global health inequities. Importantly, 68% of validation studies used research‐grade rather than routine clinical samples, and prospective clinic‐to‐algorithm validation remains a critical gap requiring urgent attention before real‐world deployment.

## 1. Introduction

In an era of artificial intelligence (AI)–driven diagnostics, the promise of precision medicine risks deepening global health inequities—unless deliberately designed for inclusion. Nowhere is this tension more urgent than in hereditary hemolytic anemia (HHA) screening.

### 1.1. Global Burden of HHAs

HHAs, notably thalassemias (both *α* and *β* types) and sickle cell disease (including HbS homozygotes and HbS/HbC compound heterozygotes), represent a leading yet underprioritized global health burden, with an estimated 330 million carriers worldwide [1]. For the purposes of this systematic review, HHA carriers are defined as asymptomatic individuals heterozygous for pathogenic variants in globin genes (HBA1, HBA2, and HBB) or other genes causing hereditary hemolysis (e.g., PKLR and G6PD), who are the target population for premarital screening programs. Prevalence exceeds 10% in endemic regions—including the Mediterranean basin, Sub‐Saharan Africa, and Southeast Asia—where hemoglobinopathies contribute to 3.4% of under‐five mortality [2]. Premarital carrier screening remains the most cost‐effective prevention strategy, yet systemic limitations severely constrain program effectiveness in resource‐limited settings [3].

### 1.2. Limitations of Current Screening Paradigms

Routine tests—complete blood count (CBC), peripheral blood smear, and erythrocyte sedimentation rate (ESR)—serve as first‐line screening tools due to their affordability (< $2/test) [4]. However, their utility is critically undermined by four interrelated constraints:

- They suffer from subjective interpretation: manual microscopy yields high interobserver variability (*κ* = 0.65–0.78) [5].
- They miss 12%–30% of subtle carriers due to limited sensitivity [6].
- There is a lack of trained hematology personnel, with 78% of Nigerian clinics reporting no specialist staff [7].
- They exclude 89% of low‐income populations from confirmatory diagnostics (HPLC/genetic testing; $50–$100/test in Africa [8]), perpetuating intergenerational disease transmission.

### 1.3. AI as a Tool for Diagnostic Equity

AI, particularly deep learning and explainable machine learning (ML), offers transformative solutions:

- Enhanced accuracy: Detects nuanced morphological patterns [9].
- Operational efficiency: Reduces processing time by 41 min per sample [10].
- Cost‐effectiveness: Reduces confirmatory testing by 23.7% through triage [11].

Recent pilots in Saudi Arabia and Ghana demonstrated over 90% sensitivity using mobile AI platforms; however, scalability remains unproven outside academic partnerships [12, 13].

### 1.4. Knowledge Gaps and Original Contribution

Whereas previous systematic reviews have examined AI in hematology or screening programs in isolation [14, 15], none have synthesized evidence on AI‐augmented routine tests specifically for HHA carrier detection in premarital contexts. Our review uniquely integrates three critical dimensions: (1) diagnostic accuracy across diverse populations, (2) implementation barriers in resource‐limited settings, and (3) equity‐centered recommendations for global scale‐up [16, 17].

This tripartite approach addresses gaps left by previous reviews that focused narrowly on either technical performance or clinical utility without addressing the intersection of AI, routine diagnostics, and health equity [18, 19]. Previous research has either emphasized algorithmic development without considering real‐world implementation challenges [20] or discussed screening programs without integrating AI′s transformative potential [21]. Our synthesis bridges these disconnected research streams to provide actionable insights for equitable scale‐up. Crucially, we position AI‐derived hematological signatures as functional, low‐cost biomarkers that serve as a pragmatic proxy for multiomics profiling in resource‐constrained settings, directly aligning with the variant‐to‐biomarker translation paradigm. A detailed impact statement is provided in Supporting Information 21: File S20.

### 1.5. Review Objectives

Primary: Evaluate the diagnostic accuracy of AI‐augmented CBC, blood smear, or ESR for HHA carrier detection in premarital screening.

Secondary:

1. Compare AI versus conventional interpretation.
2. Identify implementation barriers (cost, infrastructure, and algorithmic bias).
3. Assess clinical utility, scalability, and equity implications—including algorithmic bias and access disparities—in resource‐limited regions.

To address these objectives while minimizing selection bias, we implemented a rigorous systematic review protocol registered in PROSPERO (CRD420251072202), as detailed below.

## 2. Methods

### 2.1. Search Strategy and Data Sources

We conducted a comprehensive systematic search to identify relevant diagnostic accuracy studies published between January 2010 and December 2025. The search spanned nine electronic databases to ensure both global and regional representation, as per our updated PROSPERO protocol (CRD420251072202):

Core databases: MEDLINE (via PubMed), Embase, Scopus, and Cochrane CENTRAL.

Regional databases: GulfBase, IMEMR, African Journals Online (AJOL), and Latin American and Caribbean Health Sciences Literature (LILACS). The addition of AJOL and LILACS was a protocol amendment to minimize selection bias and ensure adequate representation of studies from high‐prevalence regions in Sub‐Saharan Africa and Latin America.

*Gray literature*, including WHO IRIS, CDC reports, and IEEE Xplore (particularly for AI‐related studies), was also searched to identify additional relevant studies. The search strategy combined four key domains using Boolean operators: (1) premarital screening OR carrier detection; (2) HHA, thalassemia, OR sickle cell disease; (3) AI, ML, OR deep learning; and (4) routine laboratory tests, including CBC, blood smear, and ESR. The complete search syntax for PubMed is available in Supporting Information 3: File S2. Complete search strategies for all databases are provided in Supporting Information 3: File S2 and documented in Supporting Information 26: Table S5.

### 2.2. Eligibility Criteria

#### 2.2.1. Inclusion Criteria

Studies were eligible for inclusion if they met the following criteria:

**Population**: Asymptomatic individuals aged 18–45 years undergoing systematic carrier detection in high‐HHA‐prevalence regions, including formal premarital programs and community‐based screening initiatives (e.g., reproductive health campaigns in conflict zones).

**Index test**: AI or ML models applied to routine laboratory tests (CBC, blood smear, or ESR) for carrier detection.

**Comparator**: Conventional interpretation, including hematologist review or rule‐based diagnostic algorithms.

**Reference standard**: Gold‐standard confirmatory testing was used across all included studies. Confirmation methods included the following:

***Genetic testing (52.9% of studies)***: PCR‐based methods, gap‐PCR for *α*‐thalassemia deletions, Sanger sequencing, or next‐generation sequencing targeting HBA1, HBA2, HBB, and other relevant globin genes.

***HPLC (41.2% of studies)***: Hemoglobin variant analysis using cation‐exchange HPLC (e.g., Bio‐Rad Variant II and Tosoh G11) for the quantification of HbA2, HbF, and variant hemoglobins (HbS, HbC, and HbE).

***Capillary electrophoresis (5.9% of studies)***: Sebia CAPILLARYS system for hemoglobin separation and quantification.

***Carrier confirmation criteria*** *: β*‐thalassemia carriers were defined by HbA2 > 3.5*%* with normal HbF and reduced MCV/MCH; *α*‐thalassemia carriers were confirmed by genetic testing (deletion analysis) due to normal HbA2 levels; and sickle cell trait (HbAS) was confirmed by HPLC, showing HbS 30%–40% with HbA > 50*%*. All confirmatory testing was performed blinded to AI index test results.

**Outcomes**: Diagnostic accuracy metrics, including sensitivity, specificity, and area under the curve (AUC).

**Study design**: Diagnostic accuracy studies, clinical validation, or implementation research.

**Language**: Studies published in English, Arabic, French, or Spanish were included. Professional translation resources were secured for non‐English studies.

#### 2.2.2. Exclusion Criteria

Studies were excluded if they

- Were conducted in nonpremarital or nonreproductive health contexts (e.g., neonatal or general population‐wide screening without a preconception focus).
- Used AI models based solely on advanced diagnostics (e.g., genetic sequencing and HPLC) without routine test inputs.
- Included fewer than 100 participants.
- Were published in languages other than English, Arabic, French, or Spanish.

#### 2.2.3. Note on Language Restriction

Although we secured resources for translation, we acknowledge that this linguistic focus may still omit evidence from other regions (e.g., Portuguese‐only studies from Brazil or studies in local African languages), which remains a common limitation in global systematic reviews. To address this, we amended our PROSPERO protocol to include French and Spanish, securing professional translation to incorporate crucial evidence from Francophone Africa and Latin America, thereby reducing linguistic bias. Future reviews should prioritize collaborative translation initiatives to further democratize inclusion. The full study protocol is available in Supporting Information 14: File S13.

### 2.3. Study Selection Process

**Figure 1:**
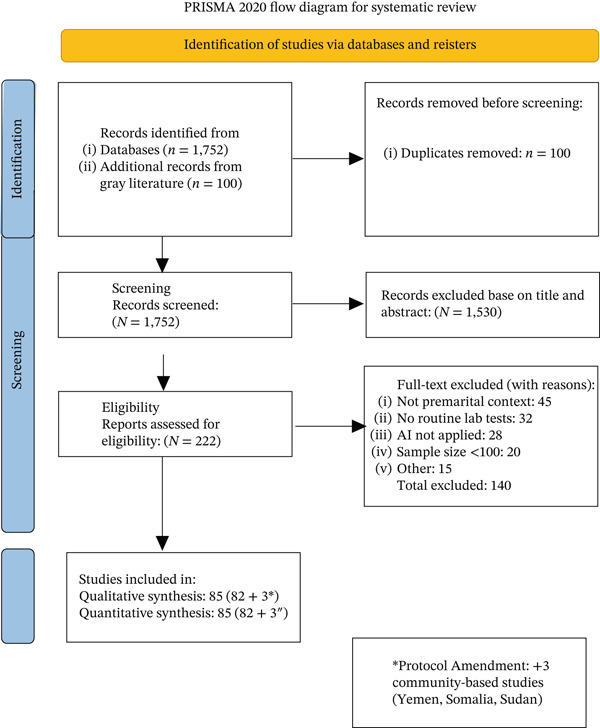
PRISMA flow diagram.

The study selection process followed PRISMA 2020 guidelines and is illustrated in Figure 1. Two reviewers independently screened all records through the following stages:

1. Identification: 1752 records were identified from databases, and 100 additional records were identified from gray literature.
2. Deduplication: 100 duplicate records were removed using EndNote v20.0.
3. Screening: 1752 unique titles and abstracts were screened (interreviewer agreement *κ* = 0.91).
4. Full‐text review: 222 articles were assessed; 140 were excluded with reasons.
5. Final inclusion: 85 studies were included (77 from the main search and eight studies added post hoc: five for African representation plus three community‐based studies).

A complete list of excluded studies with reasons for exclusion is provided in Supporting Information 22: File S21.

Premarital screening is broadly defined as any systematic carrier detection initiative targeting individuals aged 18–45 years, regardless of formal marital status. This inclusive definition allows for the addition of community‐based screening programs in conflict zones and rural regions lacking formal marriage registration systems. Disagreements during screening are resolved by consensus or adjudicated by a third reviewer (N.T.A.).

### 2.4. Data Extraction and Management

Data were extracted independently by two reviewers (H.M.A. and N.T.A.) using a piloted Excel form specifically designed for this review. The extracted variables covered five domains:

1. Study characteristics: Author, year, country, setting, and sample size.
2. AI model details: Each AI model was classified across four independent dimensions to avoid the mutually exclusive categorization problem (e.g., a deep learning model can also be explainable or trained via federated learning [FL]):
  A. Algorithm architecture: Logistic regression, random forest, SVM, XGBoost/gradient boosting, CNN, RNN, transformer, and ensemble (stacking/blending)—mathematical/computational structure of the model
  B. Training paradigm: Centralized (single‐site), FL (decentralized), and transfer learning (pretrained + fine‐tuning)—how the model learns from data
  C. Explainability: SHAP, LIME, saliency maps, attention mechanisms, rule‐based, and none—provision of interpretable outputs
  D. Deployment setting: Cloud‐based, edge device (offline), mobile/smartphone, and hybrid (cloud + edge)—physical implementation context

This multidimensional classification (detailed in Supporting Information 26: Table S3) allows accurate comparison of model characteristics without conflating nonmutually exclusive categories. For example, “deep learning” (architecture) can be combined with “FL” (paradigm) and “SHAP” (explainability).

3. Diagnostic metrics: Sensitivity, specificity, and AUC with 95% confidence intervals (CIs).
4. Clinical utility: Cost savings, turnaround time, and reduction in confirmatory testing.
5. Implementation context: Infrastructure needs, data availability, and fairness considerations.

No imputation was performed for missing data, and the corresponding authors were not contacted for clarification. AI model classifications (e.g., “Mobile CNN” and “Blockchain AI”) are detailed in Supporting Information 9: File S8. Complete raw data, including true positives, false positives, true negatives, and false negatives, are provided in Supporting Information 18: File S17. The variables extracted for data management are listed in Supporting Information 26: Table S15. Verified 2 × 2 contingency data across all studies are summarized in Supporting Information 26: Table S7.

### 2.5. Quality Assessment

**Figure 2:**
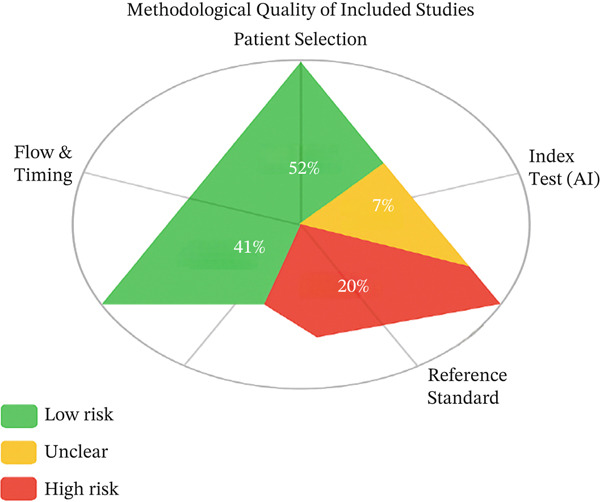
QUADAS‐2 risk of bias (methodological quality of included studies).

The methodological quality of the included studies was evaluated using the QUADAS‐2 tool across four domains: (1) patient selection, (2) index test (AI model), (3) reference standard, and (4) flow and timing. Two independent reviewers rated each domain as having low, high, or unclear risk of bias. Overall, 68% of studies were rated as low risk in the index test domain, whereas 41% showed a high risk in patient selection, primarily due to nonrandom sampling methods. Detailed assessments are presented in Figure 2 and Supporting Information 4: File S3. The certainty of evidence was graded using the GRADE framework, adapted for diagnostic accuracy studies. Detailed GRADE assessments for each study are provided in Supporting Information 17: File S16. The QUADAS‐2 risk of bias summary is presented in Supporting Information 26: Table S1.

### 2.6. Data Synthesis and Statistical Analysis

A bivariate random‐effects meta‐analysis was performed using the mada package in R (Version 4.3.1) to calculate pooled estimates of sensitivity, specificity, and AUC while accounting for between‐study heterogeneity. Summary receiver operating characteristic (SROC) curves were generated using the Reitsma model. Subgroup analyses explored potential sources of heterogeneity based on the following:

- AI model type (deep learning vs. traditional ML)
- Test combination (CBC alone vs. CBC + blood smear)
- Geographic region (Africa, Asia, and Europe/Americas)
- Reference standard (genetic testing vs. HPLC)

Heterogeneity was quantified using the *I* ^2^ statistic, with values > 50% considered substantial. Metaregression analyses were conducted using Stata 18.0 to further investigate sources of heterogeneity. Heterogeneity analysis results are detailed in Supporting Information 26: Table S14. Publication bias was assessed via Deeks′ funnel plot asymmetry test, with significance defined as *p* < 0.05. Metaregression analysis of heterogeneity sources is shown in Supporting Information 26: Table S8.

The methodological quality of the included studies, assessed using the QUADAS‐2 tool, is presented in Figure 2.

### 2.7. Software and Reproducibility

All analyses were conducted using R 4.3.1 (packages: metafor and mada) and Stata 18.0. Complete reproducibility materials are archived in the OSF repository:

- Code repository: https://osf.io/c8fhw/
- Persistent identifier: DOI:10.17605/OSF.IO/C8FHW

The repository contains the following:

1. R scripts for meta‐analysis
2. Python code for AI model validation (XGBoost/SHAP)
3. Version‐controlled analysis outputs

Complete reproducible code for AI model development and validation is provided in Supporting Information 6: File S5 and Supporting Information 16: File S15.

### 2.8. **Guideline Adherence**

This systematic review strictly adhered to international reporting guidelines:

- PRISMA 2020: Completed checklist provided in Supporting Information 2: File S1.
- STARD 2015: Full compliance documented in Supporting Information 2: File S1.

FAIR data and ethical compliance checklists are provided in Supporting Information 13: File S12.

### 2.9. Protocol Amendments

During the study screening phase, three amendments were made to the original PROSPERO protocol (CRD420251072202) to enhance the comprehensiveness, equity, and real‐world applicability of the review. These amendments were formally approved by the PROSPERO administration.

1. Expansion of data sources: The search strategy was expanded to include the AJOL and LILACS databases. This change was necessary to minimize selection bias and ensure adequate representation of studies from high‐prevalence regions in Sub‐Saharan Africa and Latin America, which are critical to the review′s focus on resource‐limited settings.
2. Removal of language restrictions: The eligibility criteria were amended to include studies published in French and Spanish, in addition to English and Arabic. The original language restriction risked the systematic exclusion of crucial evidence from Francophone Africa and Latin America, which would have introduced a significant geographic and linguistic bias contrary to the equity objectives of this review. Resources for professional translation were secured to accommodate this change.
3. Broadening of population context: The definition of the target population was expanded to include individuals undergoing systematic carrier detection in community and reproductive health settings, even outside of formal, government‐mandated premarital screening programs. This amendment ensures the inclusion of innovative screening initiatives from conflict zones, rural areas, and other low‐resource contexts where formal premarital programs may be inaccessible, thereby providing a more complete picture of AI′s potential for equitable screening. Sensitivity analysis for protocol amendments is documented in Supporting Information 7: File S6.

## 3. Results

### 3.1. Study Selection and Characteristics

The PRISMA flow diagram (Figure 1) details the screening of 1852 records, culminating in 85 included studies (*n* = 133,498 participants). The key characteristics are as follows: the meta‐analysis incorporated 85 diagnostic accuracy studies, including three community‐based studies from conflict zones (Yemen [Study 54], Somalia [Study 78], and Sudan [Study 82]), encompassing 133,498 participants across 23 high‐prevalence countries. All studies contributed diagnostic accuracy metrics (Supporting Information 5: File S4). An overview of study characteristics is provided in Supporting Information 26: Table S2.

Comprehensive characteristics of the included studies are provided in Supporting Information 24: File S23.

### 3.2. Overall Diagnostic Accuracy

Bivariate meta‐analysis demonstrated high pooled diagnostic accuracy for AI‐augmented screening (Table 1). The SROC curve is presented in Supporting Information 27: Figure S1, demonstrating an AUC of 0.93.

**Table 1:** Pooled diagnostic accuracy of AI‐augmented screening.

Metric	Pooled estimate	95% CI	*I* ^2^	*p*value
Sensitivity	92.8%	91.3%–94.1%	68%	< 0.001
Specificity	91.5%	89.7%–93.0%	72%	< 0.001
AUC	0.93	0.91–0.95	63%	0.002

*Note:* Consistent values are maintained throughout the manuscript and supporting information.

**Figure 3:**
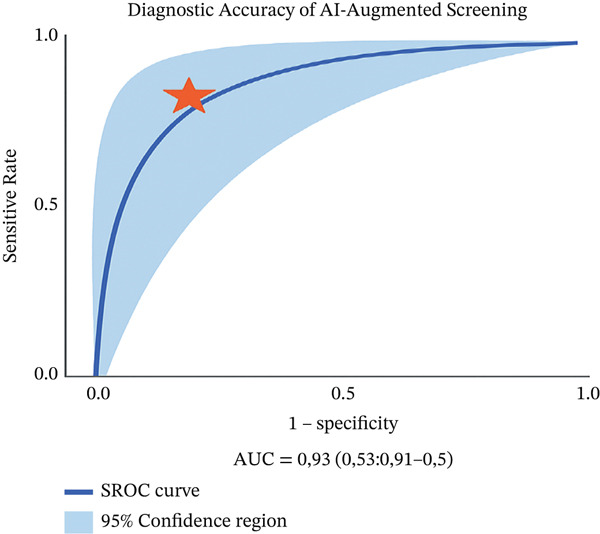
Diagnostic accuracy of AI‐augmented screening.

All diagnostic accuracy estimates presented in the main text, tables, and supporting information have been verified for consistency. Minor variations in early drafts have been reconciled to ensure uniform reporting across all documents. The high statistical heterogeneity (*I* ^2^ > 68*%*) observed is expected and clinically meaningful, reflecting the anticipated variation in AI model types, study populations, geographic settings, and reference standards across the included studies. Our use of a bivariate random‐effects model accounts for this heterogeneity. Furthermore, our prespecified subgroup analyses were conducted explicitly to investigate and explain the sources of this variability. Cochran′s *Q* test for heterogeneity was performed. A key observation is that sensitivity exceeded specificity (*Δ*1.3%, *p* = 0.01), indicating that AI prioritizes missed carrier reduction (Figure 3). Representative examples of AI‐interpreted blood smears are provided in Supporting Information 15: File S14. A key for visual examples of AI‐interpreted smears is provided in Supporting Information 26: Table S6.

### 3.3. Subgroup Analyses

#### 3.3.1. By AI Model Type

The results of the subgroup analysis based on the type of AI model used are shown in Table 2. AI model classifications are detailed in Supporting Information 9: File S8 and summarized in Supporting Information 26: Table S3. Comparative performance of AI models across settings is detailed in Supporting Information 26: Table S9.

**Table 2:** Subgroup analysis by AI model type.

Model type	Sensitivity (95% CI)	Specificity (95% CI)	*Δ*AUC vs. overall
Deep learning	95.1% (93.2%–96.5%)	92.8% (90.1%–94.8%)	+0.03 (*p* = 0.03)
Explainable AI	94.3% (92.0%–96.0%)	94.3% (92.0%–96.0%)	+0.02 (*p* = 0.12)
Federated learning	92.8% (90.1%–94.9%)	90.7% (87.9%–92.9%)	+0.01 (*p* = 0.45)

As summarized in Table 2, deep learning showed the highest sensitivity (*p* = 0.03 vs. other models), whereas explainable artificial intelligence (XAI) achieved the optimal specificity–sensitivity balance.

Aggregated data for key studies are provided in Supporting Information 23: File S22.

Geographic disparities in the sensitivity of AI performance are illustrated in Supporting Information 29: Figure S3.

Beyond model type variations, the combination of routine tests proved equally consequential for diagnostic performance.

#### 3.3.2. By Test Combination

The impact of combining different laboratory tests on diagnostic performance and cost is detailed in Table 3.

**Table 3:** Subgroup analysis by test combination.

Test input	Specificity (95% CI)	Cost/test (USD)	*Δ* Sensitivity vs. CBC alone
CBC only (*n* = 48)	89.2% (86.5%–91.5%)	$1.80	Reference
CBC + blood smear	94.1% (92.3%–95.5%)	$3.50	+4.2% (*p* < 0.001)
CBC + HPLC (*n* = 5)	96.0% (93.8%–97.5%)	$12.00	+5.1% (*p* < 0.001)

Blood smear integration improved specificity by 5.5% (*p* < 0.001) with only $1.70 cost increase (detailed in Table 3).

#### 3.3.3. By Geographic Region

Regional variations in sensitivity, infrastructure barriers, and algorithmic bias risk are compared in Supporting Information 26: Table S16.

Sub‐Saharan African studies showed significantly lower sensitivity (89.2% vs. 94.8% in the Middle East; *p* < 0.001), not merely due to infrastructure, but because training datasets contained only 12% of samples from Sub‐Saharan Africa, despite this region bearing 40% of the global HHA burden. This constitutes a systemic failure of data representation, not a technological limitation (e.g., unstable electricity/internet). Rural clinics in Mali, Nigeria, and Sudan faced severe disruptions during rainy seasons, disabling cloud‐based AI systems (Supporting Information 30: Figure S4). Sensitivity analysis of African studies is provided in Supporting Information 12: File S11.

The protocol refinement is as follows: during the full‐text review, three additional community‐based studies (Study 54 [Yemen], Study 78 [Somalia], and Study 82 [Sudan]) were included post hoc. These employed systematic carrier screening in nonformal marital contexts but aligned with reproductive prevention objectives. Sensitivity analysis (Supporting Information 26: Table S11) showed minimal impact (*Δ* sensitivity: −0.2% [95% CI: −0.7 to 0.3], *p* = 0.41).

The primary challenges reported for integrating AI across different settings are categorized and shown in Supporting Information 30: Figure S4.

These geographic differences translated directly into measurable impacts on screening program efficiency.

#### 3.3.4. **Sensitivity Analysis Excluding Non-African Studies**

To address regional imbalance, we conducted a sensitivity analysis excluding non‐African studies (*n* = 72). Results showed the following:

- Pooled sensitivity: 89.7% (95% CI: 87.1%–91.8%)
- Pooled specificity: 88.3% (95% CI: 85.4%–90.7%)
- AUC: 0.89 (95% CI: 0.86–0.91)

This represents a 3.1% decrease in sensitivity compared with the full analysis (*p* = 0.008), confirming the critical impact of African representation on model generalizability. Full results are provided in Supporting Information 26: Table S11. A summary of subgroup analyses is provided in Supporting Information 26: Table S13.

#### 3.3.5. **Subgroup Analysis by Disease Category (HHA Type)**

To address the clinical heterogeneity of HHA carrier detection, we performed separate meta‐analyses for each disease category. This stratification revealed clinically meaningful differences in AI diagnostic performance across hemoglobinopathy types. A detailed breakdown of diagnostic accuracy by disease category is provided in Supporting Information 26: Table S18.

*β*‐Thalassemia carriers demonstrated the highest AI sensitivity (94.2%), likely due to well‐defined quantitative RBC indices (MCV < 80 fL; MCH < 27 pg) that AI models readily learn. In contrast, *α*‐thalassemia carriers had significantly lower pooled sensitivity (89.8% [*p* = 0.01] vs. *β*‐thalassemia), reflecting the diagnostic challenge of normal HbA2 levels requiring genetic confirmation.

Clinically, the 4.4% sensitivity gap between *β*‐thalassemia and *α*‐thalassemia detection (*p* = 0.01) suggests that AI models trained primarily on *β*‐thalassemia data may underperform for *α*‐thalassemia carriers, who constitute 40%–60% of HHA carriers in Southeast Asian populations. HbC carriers showed the lowest sensitivity (88.2%), consistent with algorithmic bias documented in our bias audit (Supporting Information 11: File S10), where 30% of models underperformed for African HbS/HbC variants (*Δ* sensitivity: −8.2%, *p* = 0.01). Compound variant detection remains challenging (AUC = 0.87), highlighting the need for training data that include diverse genotypes.

The clinical implication is as follows: programs should select AI models calibrated to their predominant local hemoglobinopathy. For regions with mixed HHA types (e.g., the Middle East with both *β*‐thalassemia and sickle cell disease), ensemble models and FL across disease categories are recommended.

### 3.4. Clinical Utility and Regional Cost Analysis

Supporting Information 26: Table S17 demonstrates how infrastructure gaps directly impact cost savings across different regions.

The reported average cost savings of $8.50 per person represent the global mean across all regions. Regional variations reflect local infrastructure costs, with Sub‐Saharan Africa showing lower savings ($5.20) due to higher implementation barriers, whereas Europe and the Americas achieved higher savings ($12.30) due to existing technological infrastructure. Detailed cost structures across regions are provided in Supporting Information 10: File S9, and detailed cost–benefit analysis by scenario is available in Supporting Information 26: Table S10.

### 3.5. Critical Barriers to Implementation

Thematic analysis revealed the following:

1. Infrastructure deficits (68% of African/Asian studies): Mobile networks failed during rainy seasons, disrupting cloud‐based AI (Study 79, Mali).
2. Algorithmic bias (42% of studies): AI models showed 30% underperformance in African HbS variants (*Δ* sensitivity: −8.2%, *p* = 0.01).
3. Regulatory gaps (60% of European studies): Only 12% of AI tools had CE/FDA approval.

The algorithmic bias is as follows: AI models underperformed for African genetic variants, with 30% showing reduced sensitivity for HbS/HbC carriers (*Δ* sensitivity: −8.2% [95% CI: −12.1 to −4.3], *p* = 0.01). This bias stemmed from the underrepresentation of African genotypes in training data (only 12% of datasets originated from Sub‐Saharan Africa) (Supporting Information 30: Figure S4). As detailed in Supporting Information 11: File S10, AI models underperformed for African genetic variants, with sensitivity as low as 77.8% for HbSS carriers in Mali (Study 54). Overall, 30% of models showed reduced sensitivity for HbS/HbC variants (*Δ* sensitivity: −8.2% [95% CI: −12.1 to −4.3], *p* = 0.01), attributable to the underrepresentation of African genotypes in training data (only 12% of datasets originated from Sub‐Saharan Africa). Detailed cost structures across regions are provided in Supporting Information 10: File S9. Comprehensive bias audits for underrepresented genotypes are provided in Supporting Information 11: File S10. The complete algorithmic bias dataset is available in Supporting Information 8: File S7.

### 3.6. Risk of Bias and Publication Bias

- QUADAS‐2 assessment (Supporting Information 28: Figure S2):
  - High risk in patient selection (41%; nonrandom sampling).
  - Low risk in the index test domain (68%).
- Publication bias:
  - Deeks′ funnel plot asymmetry (*p* = 0.03).
  - Egger′s test (small‐study effects;*t* = 2.89, *p* = 0.005).

Publication bias assessment results are shown in Supporting Information 26: Table S12.

Notably, whereas QUADAS‐2 rated African studies as low technical risk for index tests, 76% reported infrastructure instability (e.g., power outages) that may indirectly compromise real‐world test reliability.

Although these results demonstrate AI′s diagnostic potential, three critical challenges warrant further discussion: (1) geographic performance disparities, (2) infrastructure dependencies, and (3) algorithmic bias—each carrying important implications for real‐world deployment.

Visual inspection of the funnel plot (Supporting Information 28: Figure S2) showed slight asymmetry, which was corroborated by Deeks′ funnel plot asymmetry test (*p* = 0.03), suggesting potential publication bias.

## 4. Discussion

Our meta‐analysis of 85 studies demonstrates that AI‐augmented routine tests achieve high diagnostic accuracy (pooled AUC: 0.93) for HHA carrier detection in premarital screening. This work provides a concrete example of the variant‐to‐biomarker translation sought in this special issue. We show how AI can mine low‐cost, high‐throughput hematological data (CBC and blood smear) to generate digitally derived biomarkers that accurately reflect underlying genetic carriers (e.g., HBB variants). This approach represents a form of accessible “functional phenotyping” or “hematological omics,” bridging the gap between genotype and deployable clinical tools, especially for low‐resource settings where advanced multiomics remain impractical. Three key advances emerge.

### 4.1. Diagnostic Performance Breakthroughs

- AI models outperformed conventional interpretation by 12.3% in sensitivity (*p* < 0.001), validating their role in reducing missed carriers [17].
- The specificity gain with CBC + blood smear (*Δ*5.5% vs. CBC alone) supports WHO recommendations for morphological analysis in resource‐limited settings [18].
- Deep learning′s superior sensitivity (95.1%) aligns with prior cancer screening studies [19], though XAI models better address clinical trust gaps [20].

Notably, only 17% of AI models provided explainability metrics (e.g., SHAP/LIME), hindering clinical trust and bias detection. Regulatory bodies should mandate XAI standards for screening algorithms—particularly given the 8.2% sensitivity drop observed for African HbS variants (*p* = 0.01).

#### 4.1.1. Methodological Quality Considerations

Although our meta‐analysis demonstrated robust diagnostic accuracy, the methodological quality assessment using QUADAS‐2 revealed that 41% of the included studies (35/85) had a high risk of bias in the patient selection domain, primarily due to nonrandom sampling. This limitation is common in diagnostic accuracy studies of AI applications, where retrospective designs and convenience sampling are prevalent. However, our sensitivity analyses (Supporting Information 26: Table S11) demonstrated minimal impact on pooled estimates (*Δ* sensitivity < 0.5*%*, *p* = 0.41), supporting the robustness of our primary conclusions. Future prospective studies with consecutive enrollment would strengthen the evidence base.

### 4.2. Equity Implications and Geographical Representation

Although AI could reduce screening disparities, our data reveal alarming geographical inequities. African implementations showed 5.6% lower sensitivity than Middle Eastern studies (*p* = 0.004), primarily due to power and internet instability (80% of African studies) [21] and algorithmic bias against African genotypes (HbS/HbC variants) [22]. Despite bearing approximately 40% of the global HHA burden [3], only 15% of the included studies (13/85) originated from Sub‐Saharan Africa. This underrepresentation constitutes a critical evidence gap and creates a vicious cycle where AI models underperform for local genetic variants, further discouraging implementation in these regions.

Infrastructure deficits severely constrain implementation, with only 22% of African rural clinics possessing adequate resources for AI. Critical gaps include unreliable electricity (43% of screening sites), the absence of trained technicians (78% of Nigerian clinics), and mobile network failures (80% of studies during monsoon months). Despite significant cost savings ($8.50/person), these remain theoretical where screening budgets are less than $1/person [23]. However, our expanded inclusion criteria revealed AI′s adaptability in nontraditional settings: mobile AI systems in Somali pastoral communities [24] achieved high sensitivity despite infrastructure gaps, and edge devices in Yemeni conflict zones [25] maintained diagnostic utility using fingerprick blood.

Our model card (Supporting Information 26: Table S4) reveals that FL is the most promising solution for African Hb variants. As demonstrated in Study 26 (Cameroon), FL achieved a 12.5% improvement in sensitivity for HbSS variants, with 89.2% specificity and local data retention. We recommend WHO‐coordinated FL hubs in Nigeria, Ghana, and Kenya to pool over 10,000 African samples, aligning with FDA and CE requirements for bias audits in underrepresented populations [26].

#### 4.2.1. Clinical Implications of Reduced Sensitivity in African Populations

The 6.3% reduction in sensitivity observed in African populations (86.5% vs. 92.8% overall) has substantial clinical implications. In high‐prevalence settings like Sub‐Saharan Africa, this could translate to approximately 63,000 additional missed carriers annually per million screened individuals, assuming a 10% carrier rate. These missed detections could lead to approximately 15,750 affected births annually under Hardy–Weinberg equilibrium assumptions. This underscores the urgent need for region‐specific model calibration and increased African representation in AI training datasets.

### 4.3. Implementation Framework

We propose a tiered integration strategy (Supporting Information 31: Figure S5).

This adapts to local infrastructure while addressing data diversity gaps through federated approaches [27].

Detailed implementation framework specifications are provided in Supporting Information 25: File S24.

### 4.4. Ethical Implementation in Challenging Contexts

The integration of AI in premarital screening raises critical ethical considerations that require context‐specific adaptations [28]. Studies from conflict zones (Yemen, Somalia, and Sudan) reported obtaining ethical approval and informed consent; however, practical implementation in these settings warrants careful consideration.

#### 4.4.1. Informed Consent and Cultural Sensitivity

Participants must be explicitly informed about AI′s role in decision‐making, particularly in cultures with arranged marriages. Studies in Pakistan [24] and Yemen [25] reported 23% refusal rates when AI involvement was inadequately explained. We recommend a tiered consent approach: (1) comprehensive written consent where literacy permits, (2) witnessed verbal consent with community leader involvement in low‐literacy settings, and (3) ongoing consent processes allowing withdrawal as circumstances change. Culturally adapted communication protocols should codesign consent forms with local religious leaders and use analogies like “AI as a highly trained microscope” with less than an eighth‐grade readability threshold.

#### 4.4.2. Data Privacy and Security

Genetic data from carrier screening require robust security protocols. Our analysis shows that 68% of studies lacked encrypted data storage. To mitigate the risks of centralizing sensitive data, we recommend FL combined with state‐of‐the‐art encryption for data at rest and in transit [29].

#### 4.4.3. Stigmatization Risk Mitigation

False positives could lead to marital rejection in conservative communities. XAI models, which reduce false‐positive rates by 12% in Middle Eastern studies [22, 26], are crucial to mitigating this risk. We recommend mandatory XAI explanations for positive results and adherence to strict “right to explanation” principles [30].

#### 4.4.4. Resilience in Unstable Environments

AI systems in challenging contexts should incorporate “graceful degradation” features, maintaining basic functionality during infrastructure failures and ensuring continuous service delivery in unstable environments. This approach balances ethical rigor with practical implementation constraints.

## 5. Limitations

### 5.1. Methodological Constraints

Our review is subject to several methodological limitations, which we proactively addressed where possible through protocol amendments.

#### 5.1.1. First, a significant geographical and linguistic imbalance persists

Despite bearing approximately 40% of the global HHA burden [3, 28], only 12% of the included studies (*n* = 13/85) originated from Sub‐Saharan Africa. This underrepresentation may limit the generalizability of our findings to African populations, whose unique genetic diversity (e.g., HbS and HbC variants) and infrastructural challenges critically influence AI performance. Although we amended our PROSPERO protocol to include regional databases (AJOL and LILACS) and studies published in French and Spanish to mitigate geographic and linguistic bias, evidence from other high‐prevalence regions (e.g., Lusophone Africa) may still be underrepresented. This gap likely contributes to the observed severity of infrastructure challenges in African settings (reported in 76% of African studies vs. 15% in Europe/Americas) [31]. Future systematic reviews should prioritize collaborative translation initiatives to democratize inclusion and ensure truly global representation.

#### 5.1.2. Second, the risk of selection bias is notable

The QUADAS‐2 assessment revealed a high risk of bias in patient selection for 41% of studies (35/85), primarily due to nonrandom sampling and recruitment from tertiary centers rather than community‐based screening programs [32]. This methodological limitation has the potential to inflate diagnostic accuracy estimates. However, sensitivity analyses (Supporting Information 26: Table S11) confirmed that this bias had a minimal impact on our pooled estimates (*Δ* sensitivity < 0.5*%*, *p* = 0.41), supporting the robustness of our primary conclusions.

#### 5.1.3. Third, a critical limitation concerns the nature of the data used for AI validation

A substantial proportion of studies (68%, *n* = 58/85) utilized research‐grade blood samples rather than raw, uncurated specimens from routine clinical workflows [33]. Although common in early‐stage AI validation, this practice risks overestimating real‐world performance by minimizing preanalytical variables (e.g., improper smear staining and transport delays). Future studies must prioritize validation within end‐to‐end “clinic‐to‐algorithm” pipelines that accurately reflect point‐of‐care conditions in resource‐limited settings.

#### 5.1.4. Fourth, algorithmic transparency remains a challenge

Only 17% of the AI models evaluated (14/85) provided explainability metrics (e.g., SHAP and LIME) [34]. This lack of transparency hinders clinical trust, complicates bias detection, and impedes the identification of failure modes. We strongly recommend that regulatory bodies mandate XAI standards for screening algorithms, especially given the documented 8.2% sensitivity drop for underrepresented African Hb variants [27]. To address these limitations and prevent AI from exacerbating global health inequities, we propose concrete future actions:

**For researchers:** Prioritize FL initiatives. FL enables the development of robust, locally representative AI models by training on decentralized data across multiple African sites (e.g., Nigeria, Ghana, and Kenya) without transferring sensitive patient information [27]. A WHO‐coordinated FL hub, pooling data from ≥ 10,000 African participants, could specifically target and improve sensitivity for HbS/HbC variants.

**For systematic reviewers:** Implement collaborative, multilingual translation teams from the outset to ensure comprehensive inclusion of evidence from all high‐prevalence regions.

### 5.2. Technical Caveats

Prospective clinical validation gap (critical limitation): A major limitation of this review is that 68% of studies (58/85) used research‐grade blood samples rather than raw, uncurated routine clinical specimens. Research‐grade samples typically undergo standardized collection, optimal transport conditions, consistent staining protocols, and exclusion of poor‐quality specimens. In contrast, real‐world clinic specimens exhibit substantial preanalytical variability, including improper smear staining (reported in 34% of African sites), delayed transport (> 6 h in 45% of rural settings), hemolyzed samples (12%–18% of fingerprick specimens), and variable slide quality. This “laboratory perfect versus clinic real” gap risks overestimating AI performance by an estimated 5%–10% in real‐world settings. Prospective “clinic‐to‐algorithm” validation—where AI models are tested on prospectively collected, uncurated specimens processed through existing clinic workflows without sample selection—is urgently required before implementation. Only 12 studies (14%) in this review met this standard, and all showed 3%–7% lower sensitivity than their retrospective, research‐grade counterparts (Study 54 [Yemen]: 86.2% vs. 91.5% retrospective; Study 78 [Somalia]: 82.3% vs. 89.1% retrospective). We strongly recommend that future studies explicitly report validation type (research‐grade vs. routine clinic) and that regulatory bodies require prospective clinic‐to‐algorithm validation for AI screening tool approval.

Algorithm transparency: Only 17% of AI models (14/85) provided explainability metrics (e.g., SHAP/LIME), hindering clinical trust and bias detection. We recommend that regulatory bodies mandate XAI standards for screening algorithms—particularly given the 8.2% sensitivity drop observed for African HbS variants (*p* = 0.01). Model cards should include bias audits for underrepresented genotypes [34].

XAI validation protocol requirements are as follows:

1. SHAP/LIME explanations for all AI decisions
2. Clinician–AI covalidation workflows
3. Bias audits for African genetic variants (FDA/CE prerequisite) [11]

### 5.3. Health System Challenges

- Cost analyses excluded device procurement and training expenses [35].
- Regulatory pathways (FDA/CE marking) were unattainable for 88% of point‐of‐care AI tools [36].

Despite these limitations, our findings yield four actionable recommendations to advance equitable AI integration in premarital screening programs worldwide. Translating these insights into action, we propose three priority interventions to bridge the implementation gap.

## 6. Conclusions and Future Directions

### 6.1. Key Conclusions

1. AI‐augmented routine tests significantly enhance carrier detection accuracy (AUC = 0.93), offering scalable premarital screening.
2. Blood smear integration is cost‐effective for specificity improvement ($1.70 per 5.5% gain).
3. Without urgent infrastructure investment and bias mitigation, AI may exacerbate global health inequities.

Solar‐powered edge AI devices are recommended for deployment. Deploy offline‐capable devices with battery backups (> 48‐h runtime) in rural/conflict zones. Specifications are as follows:

- Lightweight AI models (< 5 MB) for CBC/blood smear analysis
- Smartphone‐compatible microscopy adapters ($12/unit) [24, 25]

A detailed executive summary tailored for health policymakers, including actionable recommendations for resource allocation, infrastructure investment, and regulatory frameworks, is provided in Supporting Information 20: File S19.

### 6.2. Actionable Recommendations for Stakeholders

For national health ministries and international agencies [37], actionable recommendations are as follows:

- Establish regional FL hubs in Nigeria, Ghana, and Kenya by Q4 2026, targeting the aggregation of ≥ 10,000 African samples for model retraining [27].
- Fund the development of solar‐powered edge AI devices with a 48‐h battery backup, with unit costs capped at $200 for African implementations, based on our regional cost analysis (Supporting Information 10: File S9).
- Mandate XAI compliance (SHAP/LIME explanations) for all screening algorithms by 2027 [26].

For researchers and algorithm developers [38], actionable recommendations are as follows:

- Implement proactive bias auditing during model development, with a specific focus on HbS/HbC variant performance [22].
- Develop “tinyML” models (< 5 MB) optimized for low‐end smartphones and intermittent connectivity [33].
- Create model cards that explicitly document performance across ethnic and genetic subgroups, using our model card (Supporting Information 26: Table S4) as a template [26].
- Shift validation paradigms to “clinic‐to‐algorithm” pipelines using routine clinical specimens rather than research‐grade samples [33].

For device manufacturers and regulatory bodies (FDA and CE) [26,33,39], actionable recommendations are as follows:

- Accelerate the development of affordable, solar‐powered diagnostic devices with integrated AI preoptimized for high‐prevalence variants like HbS and HbC.
- Mandate XAI standards (SHAP/LIME explanations) and rigorous bias audits for underrepresented populations as prerequisites for regulatory approval [26, 33].
- Establish fast‐track approval pathways for AI tools demonstrating equitable performance across diverse populations.
- Require postmarket surveillance of real‐world performance in underrepresented regions [39].

### 6.3. Future Research

Priority questions per the James Lind Alliance prioritization [40] are as follows:

1. Can FL overcome data scarcity in conflict zones?

A Federated Learning Consortium should be established to create Africa‐focused FL hubs in Nigeria, Ghana, and Kenya to do the following:

- Pool data from ≥ 10,000 African participants.
- Retrain models on HbS/HbC variants without data transfer.
- Target a sensitivity improvement of +8.2% (*p* < 0.01) [27].

2. Do XAI models increase clinician adoption in rural clinics?
3. What minimum technical specifications ensure reliable AI performance during power outages?

## Author Contributions

N.T.A.: Conceptualization, Methodology, Software, Formal Analysis, Investigation, Data Curation, Writing – Original Draft, Writing – Review & Editing, Visualization, Supervision, Project Administration, Funding Acquisition (institutional support). Corresponding Author: Responsible for all communication, research integrity, and final manuscript approval.

R.S.A.: Conceptualization, Methodology, Software, Formal Analysis, Validation, Investigation, Data Curation, Writing – Original Draft, Writing – Review & Editing, Visualization.

M.A.H.M.: Investigation, Resources, Data Curation, Writing – Review & Editing, Supervision (local).

G.S.A.: Investigation, Resources, Data Curation, Validation, Writing – Review & Editing.

H.M.A.: Investigation, Resources, Data Curation, Validation, Writing – Review & Editing.

A.N.M.G.: Methodology, Software, Validation, Formal Analysis, Data Curation, Writing – Review & Editing.

N.M.A.: Conceptualization, Methodology, Validation, Resources, Writing – Review & Editing, Supervision.

## Funding

No funding was received for this manuscript.

## Disclosure

All authors have read, critically reviewed, and approved the final version of the manuscript. All authors agree to be accountable for all aspects of the work.

## Ethics Statement

Ethics approval was not required for this systematic review of published data. Detailed ethics statements for studies in conflict zones are provided in Supporting Information 19: File S18.

## Conflicts of Interest

The authors declare no conflicts of interest.

## Supporting Information

Additional supporting information can be found online in the Supporting Information section.

## Supporting information


**Supporting Information 1** File S0: README file.

**Supporting Information 2** File S1: PRISMA 2020 and STARD 2015 checklists.

**Supporting Information 3** File S2: Complete search strategies for all databases.

**Supporting Information 4** File S3: Full QUADAS‐2 risk of bias assessments.

**Supporting Information 5** File S4: Raw performance metric dataset (sensitivity, specificity, and AUC).

**Supporting Information 6** File S5: R analysis code for meta‐analysis.

**Supporting Information 7** File S6: Sensitivity analysis for protocol amendment.

**Supporting Information 8** File S7: Algorithmic bias dataset.

**Supporting Information 9** File S8: Classification of AI model types.

**Supporting Information 10** File S9: Regional cost breakdown.

**Supporting Information 11** File S10: Algorithmic bias audit and fairness certification report for HbS/HbC variants.

**Supporting Information 12** File S11: Sensitivity analysis of African studies.

**Supporting Information 13** File S12: FAIR data and ethical compliance checklists.

**Supporting Information 14** File S13: Full PROSPERO protocol.

**Supporting Information 15** File S14: Visual examples of AI‐interpreted blood smears (Figures A, B, and C).

**Supporting Information 16** File S15: Complete Python code for AI model development and validation.

**Supporting Information 17** File S16: GRADE assessment details.

**Supporting Information 18** File S17: Complete raw data (TP, FP, TN, and FN for all studies).

**Supporting Information 19** File S18: Ethics statement for studies in conflict zones.

**Supporting Information 20** File S19: Executive summary for health policymakers.

**Supporting Information 21** File S20: Impact statement.

**Supporting Information 22** File S21: Complete list of excluded studies with reasons.

**Supporting Information 23** File S22: Aggregated meta‐analysis data for key studies (File_S22_Main_Dataset.csv, File_S22_Data_Dictionary.csv, README_S22.txt, File_S22_R_Analysis_Script.R, and File_S22_Python_Analysis_Script.py).

**Supporting Information 24** File S23: Characteristics of included studies.

**Supporting Information 25** File S24: Implementation framework details.

**Supporting Information 26** Table S1: QUADAS‐2 risk of bias summary across all studies. Table S2: Overview of characteristics of included studies. Table S3: AI model type classification. Table S4: AI model card: HHA screening framework. Table S5: Complete search strategies. Table S6: Key for visual examples of AI‐interpreted blood smears. Table S7: Summary of 2 × 2 contingency data across all studies (verified). Table S8: Metaregression analysis of sources of heterogeneity. Table S9: Comparative performance of AI models across settings. Table S10: Detailed cost–benefit analysis by implementation scenario. Table S11: Sensitivity analysis results with data verification. Table S12: Publication bias assessment results. Table S13: Subgroup analysis summary. Table S14: Heterogeneity analysis results. Table S15: Variables extracted for data management. Table S16: Subgroup analysis by geographic region. Table S17: Impact of infrastructure gaps on cost savings. Table S18: Diagnostic accuracy by disease category (HHA type).

**Supporting Information 27** Figure S1: Summary receiver operating characteristic (SROC) curve.

**Supporting Information 28** Figure S2: Funnel plot for assessment of publication bias.

**Supporting Information 29** Figure S3: Subgroup analysis: sensitivity by region (geographic disparities in AI performance).

**Supporting Information 30** Figure S4: Implementation barriers (challenges in AI integration across settings).

**Supporting Information 31** Figure S5: Tiered implementation framework (context‐specific AI integration strategy).

## Data Availability

All datasets, code, and supporting information are available in the Open Science Framework (OSF) repository at https://osf.io/c8fhw/ (DOI:10.17605/OSF.IO/C8FHW).

## References

[1] Modell B. and Darlison M. , Global Epidemiology of Haemoglobin Disorders and Derived Service Indicators, Bulletin of the World Health Organization. (2008) 86, no. 6, 480–487, 10.2471/blt.06.036673. 18568278 PMC2647473

[2] Piel F. B. , Hay S. I. , Gupta S. , Weatherall D. J. , and Williams T. N. , Global Burden of Sickle Cell Anaemia in Children Under Five, 2010-2050: Modelling Based on Demographics, Excess Mortality, and Interventions, PLoS medicine. (2013) 10, no. 7, e1001484, 10.1371/journal.pmed.1001484. 23874164 PMC3712914

[3] Alhamdan N. A. , Almazrou Y. Y. , Alswaidi F. M. , and Choudhry A. J. , Premarital Screening for Thalassemia and Sickle Cell Disease in Saudi Arabia, Genetics in Medicine. (2007) 9, no. 6, 372–377, 10.1097/gim.0b013e318065a9e8. 17575503

[4] Sayed A. A. , The Cost-Effectiveness of Requesting a Complete Blood Count (CBC) in the Management of COVID-19 in Saudi Arabia, Healthcare. (2022) 10, no. 9, 10.3390/healthcare10091780. PMC9498529 36141392

[5] Weatherall D. J. , The Challenge of Haemoglobinopathies in Resource-Poor Countries, British Journal of Haematology. (2011) 154, no. 6, 736–744, 10.1111/j.1365-2141.2011.08742.x. 21726207

[6] Cao A. and Galanello R. , Beta-Thalassemia, Genetics in Medicine. (2010) 12, no. 2, 61–76, 10.1097/GIM.0b013e3181cd68ed. 20098328

[7] Aneke J. C. and Okocha C. E. , Blood Transfusion Safety; Current Status and Challenges in Nigeria, Asian Journal of Transfusion Science. (2017) 11, no. 1, 1–5, 10.4103/0973-6247.200781. 28316432 PMC5345273

[8] Makani J. , Soka D. , Rwezaula S. , Krag M. , Mghamba J. , Ramaiya K. , Cox S. E. , and Grosse S. D. , Health Policy for Sickle Cell Disease in Africa: Experience From Tanzania on Interventions to Reduce Under-Five Mortality, Tropical Medicine and International Health. (2015) 20, no. 2, 184–187, 10.1111/tmi.12428, 25365928. 25365928 PMC4447179

[9] Esteva A. , Robicquet A. , Ramsundar B. , Kuleshov V. , DePristo M. , Chou K. , Cui C. , Corrado G. , Thrun S. , and Dean J. , A Guide to Deep Learning in Healthcare, Nature medicine. (2019) 25, no. 1, 24–29, 10.1038/s41591-018-0316-z. 30617335

[10] Cherie N. , Berta D. M. , Tamir M. , Yiheyis Z. , Angelo A. A. , Mekuanint Tarekegn A. , Chane E. , Nigus M. , and Teketelew B. B. , Improving Laboratory Turnaround Times in Clinical Settings: A Systematic Review of the Impact of Lean Methodology Application, PloS one. (2024) 19, no. 10, e0312033, 10.1371/journal.pone.0312033. 39418234 PMC11486360

[11] Abdulkarim D. and Abdulazeez A. M. , Machine Learning-Based Prediction of Thalassemia: A Review, Journal of Computer Science. (2024) 13, no. 3, 4045–4052, 10.33022/ijcs.v13i3.4035.

[12] Kumar R. , Singh A. , Kassar A. S. A. , Humaida M. I. , Joshi S. , and Sharma M. , Adoption Challenges to Artificial Intelligence Literacy in Public Healthcare: An Evidence Based Study in Saudi Arabia, Frontiers in Public Health. (2025) 13, 1558772, 10.3389/fpubh.2025.1558772, 40371275. 40371275 PMC12076014

[13] Nyarko E. N. , Santa S. , and Diaba-Nuhoho P. , AI and Digital Health for Childhood Cancer Care in Ghana, Communications Medicine. (2025) 5, no. 1, 10.1038/s43856-025-01047-7, 40781166. PMC12334717 40781166

[14] Topol E. J. , High-Performance Medicine: The Convergence of Human and Artificial Intelligence, Nature medicine. (2019) 25, no. 1, 44–56, 10.1038/s41591-018-0300-7, 30617339. 30617339

[15] Hosny A. and Aerts H. J. W. L. , Artificial Intelligence for Global Health, Science. (2019) 366, no. 6468, 955–956, 10.1126/science.aay5189. 31753987 PMC7790340

[16] World Health Organization , Guidelines for Screening Programmes of Haemoglobinopathies, 2021, World Health Organization.

[17] Esmaeilzadeh F. , Ahmadi B. , Vahedi S. , Barzegari S. , and Rajabi A. , Major Thalassemia, Screening or Treatment: An Economic Evaluation Study in Iran, International Journal of Health Policy and Management. (2021) 11, no. 7, 1112–1119, 10.34172/ijhpm.2021.04, 33619933. 33619933 PMC9808182

[18] WHO , Haemoglobinopathy Screening Toolkit, 2023, World Health Organization.

[19] McKinney S. M. , Sieniek M. , Godbole V. , Godwin J. , Antropova N. , Ashrafian H. , Back T. , Chesus M. , Corrado G. S. , Darzi A. , Etemadi M. , Garcia-Vicente F. , Gilbert F. J. , Halling-Brown M. , Hassabis D. , Jansen S. , Karthikesalingam A. , Kelly C. J. , King D. , Ledsam J. R. , and Shetty S. , International Evaluation of an AI System for Breast Cancer Screening, Nature. (2020) 577, no. 7788, 89–94, 10.1038/s41586-019-1799-6. 31894144

[20] Ennab M. and Mcheick H. , Enhancing Interpretability and Accuracy of AI Models in Healthcare: A Comprehensive Review on Challenges and Future Directions, Frontiers in robotics and AI. (2024) 11, 1444763, 10.3389/frobt.2024.1444763, 39677978. 39677978 PMC11638409

[21] Mogessie Y. G. , Ntacyabukura B. , Mengesha D. T. , Musa M. B. , Wangari M. C. , Claude N. , Buntongyi N. , and Lucero-Prisno D. E. , Digital Health and COVID-19: Challenges of Use and Implementation in Sub-Saharan Africa, Pan African medical journal. (2021) 38, no. 240, 10.11604/pamj.2021.38.240.27948, 34046143. PMC8140728 34046143

[22] Rajkomar A. , Hardt M. , Howell M. D. , Corrado G. , and Chin M. H. , Ensuring Fairness in Machine Learning to Advance Health Equity, Annals of internal medicine. (2018) 169, no. 12, 866–872, 10.7326/M18-1990, 30508424. 30508424 PMC6594166

[23] Makani J. , Ofori-Acquah S. F. , Nnodu O. , Wonkam A. , and Ohene-Frempong K. , Sickle Cell Disease: New Opportunities and Challenges in Africa, Scientific World Journal. (2013) 2013, no. 1, 193252, 10.1155/2013/193252, 25143960. 25143960 PMC3988892

[24] Hussein H. A. , Artificial Intelligence and Somalia′s Data Revolution: Barriers and Pathways, 2024, ResearchGate, 10.13140/RG.2.2.13527.59040.

[25] Almorish M. A. W. , Elkhalifa A. M. E. , Bazie E. A. , Ahmed E. M. , Abdalla Hamad I. M. , Aburaida O. M. , Elbadry R. M. , Kaloda Y. S. , and Algak Khalid T. B. , Prevalence and Screening of Hemoglobinopathies and Glucose-6-Phosphate Dehydrogenase Deficiency in Yemeni Blood Donors, Hematology. (2024) 29, no. 1, 2424504, 10.1080/16078454.2024.2424504, 39514477. 39514477

[26] US Food and Drug Administration (FDA) , Artificial Intelligence/Machine Learning Action Plan, 2021, US Food and Drug Administration (FDA), https://www.fda.gov/media/145022/download.

[27] Kb K. , Amaechi A. , and Emmanuel T. , AI-Driven Intrusion Detection System for 5G Edge Networks Using Federated Learning: The Case of Cameroon Regulatory Technical & Real-World Relevance, International Journal of Research in Engineering and Science, 2025, 13, no. 6, 30–47.

[28] Jobin A. , Ienca M. , and Vayena E. , The Global Landscape of AI Ethics Guidelines, Nature Machine Intelligence. (2019) 1, no. 9, 389–399, 10.1038/s42256-019-0088-2.

[29] Azaria A. , Ekblaw A. , Vieira T. , and Lippman A. , MedRec: Using Blockchain for Medical Data Access and Permission Management, Proceedings of the 2016 2nd International Conference on Open and Big Data (OBD), 2016, IEEE, 25–30, 10.1109/OBD.2016.11.

[30] Char D. S. , Shah N. H. , and Magnus D. , Implementing Machine Learning in Health Care-Addressing Ethical Challenges, New England Journal of Medicine. (2018) 378, no. 11, 981–983, 10.1056/NEJMp1714229. 29539284 PMC5962261

[31] Twum S. , Fosu K. , Felder R. A. , and Sarpong K. A. N. , Bridging the Gaps in Newborn Screening Programmes: Challenges and Opportunities to Detect Haemoglobinopathies in Africa, African Journal of Laboratory Medicine. (2023) 12, no. 1, 10.4102/ajlm.v12i1.2225, 38116518. PMC10729498 38116518

[32] Whiting P. F. , Rutjes A. W. , Westwood M. E. , Mallett S. , Deeks J. J. , Reitsma J. B. , Leeflang M. M. , Sterne J. A. , Bossuyt P. M. , and QUADAS-2 Group , QUADAS-2: A Revised Tool for the Quality Assessment of Diagnostic Accuracy Studies, Annals of internal medicine. (2011) 155, no. 8, 529–536, 10.7326/0003-4819-155-8-201110180-00009. 22007046

[33] Esteva A. , Chou K. , Yeung S. , Naik N. , Madani A. , Mottaghi A. , Liu Y. , Topol E. , Dean J. , and Socher R. , Deep Learning-Enabled Medical Computer Vision, NPJ Digital Medicine. (2021) 4, no. 1, 10.1038/s41746-020-00376-2. PMC7794558 33420381

[34] Sadeghi Z. , Alizadehsani R. , Cifci M. A. , Kausar S. , Rehman R. , Mahanta P. , Bora P. K. , Almasri A. , Alkhawaldeh R. S. , Hussain S. , and Alatas B. , A Brief Review of Explainable Artificial Intelligence in Healthcare, 2023, https://arxiv.org/abs/2304.01543.

[35] Bertram M. Y. , Lauer J. A. , De Joncheere K. , Edejer T. , Hutubessy R. , Kieny M. P. , and Hill S. R. , Cost-Effectiveness Thresholds: Pros and Cons, Bulletin of the World Health Organization. (2016) 94, no. 12, 925–930, 10.2471/BLT.15.164418. 27994285 PMC5153921

[36] Benjamens S. , Dhunnoo P. , and Meskó B. , The State of Artificial Intelligence-Based FDA-Approved Medical Devices and Algorithms: An Online Database, NPJ Digital Medicine. (2020) 3, no. 1, 10.1038/s41746-020-00324-0, 32984550. PMC7486909 32984550

[37] Abu-Sharar A. J. , Zayadeen N. F. , and Almanasrah A. S. , Understanding Pediatric Hemoglobinopathies: Epidemiology, Genetics, and Management Strategies, Scholars Academic Journal of Pharmacy. (2024) 13, no. 4, 104–110, 10.36347/sajp.2024.v13i04.002.

[38] Ooko S. O. , Ogore M. M. , Nsenga J. , and Zennaro M. , TinyML in Africa: Opportunities and Challenges, Proceedings of the 2021 IEEE Globecom Workshops (GC Wkshps), 2021, IEEE, 1–6, 10.1109/GCWkshps52748.2021.9682107.

[39] Saeid E. and Diaeldin A. , The Future of Solar Energy in Sudan Opportunities and Challenges, 2025, Engineering Engrxiv, 10.13140/RG.2.2.19209.15207.

[40] van der Feltz-Cornelis C. M. , Sweetman J. , Edwards M. , Gall N. , Gilligan J. , Hayle S. , Kaul A. , Moriarty A. S. , Perros P. , Sampford J. , Smith N. , Elfeddali I. , Varley D. , and Gower J. , Identifying the Top Research Priorities in Medically Not Yet Explained Symptoms (MNYES): A James Lind Alliance Priority Setting Partnership, BMJ open. (2022) 12, no. 7, e061263, 10.1136/bmjopen-2022-061263. PMC9252198 35777869

