# Supplementary material for: AI‐Augmented Hematological Signatures for Equitable Detection of Hereditary Hemolytic Anemia Carriers: A Global Systematic Review and Meta‐Analysis
Source: Hum Mutat. 2026 Jun 27;2026:9405486. doi: 10.1155/humu/9405486 (PMC13309745; doi:10.1155/humu/9405486)
Supplement: Supplementary file 2 — Supporting Information 2 File S1: PRISMA 2020 and STARD 2015 checklists. [file HUMU-2026-9405486-s012.docx]

**File S1: Study Checklists**

1. PRISMA 2020 Checklist

2. STARD 2015 Checklist

3. STARD-AI Compliance Checklist

4. PRISMA-AI Compliance Checklist

**1. PRISMA 2020 Checklist for Systematic Reviews**

| Section/Topic | Item # | Checklist Item | Location in Manuscript |
| --- | --- | --- | --- |
| TITLE | 1 | Identify the report as a systematic review | Title Page: “A Systematic Review and Meta-Analysis…” |
| ABSTRACT | 2 | Provide a structured summary | Page 2: Abstract section (Background, Methods, Results, Conclusions) |
| INTRODUCTION | 3 | Describe the rationale for the review | Page 4-5: Introduction, Sections 1.1 - 1.4 |
|  | 4 | State the objectives | Page 5: Introduction, Section 1.5 |
| METHODS | 5 | Indicate whether a review protocol exists | Page 6: Section 2.1 (PROSPERO CRD420251072202) |
|  | 6 | Specify the registration number | Page 6: Section 2.1 (PROSPERO CRD420251072202) |
|  | 7 | Describe all information sources | Page 6: Section 2.1 (Databases listed) |
|  | 8 | Present full search strategies | Supplementary File S2 |
|  | 9 | Specify the date of each search | Supplementary File S2 (Date of Search: June 30, 2025) |
|  | 10 | Explain the process for selecting studies | Page 7-8: Section 2.3 (PRISMA Flow Diagram, Figure 1) |
|  | 11 | Describe the method of data extraction | Page 9: Section 2.4 (Dual extraction using piloted form) |
|  | 12 | List and define all data variables | Page 9: Table 2.1 (Variables Extracted) |
|  | 13 | List and define all outcomes | Page 7: Section 2.2 (Outcomes: Sensitivity, Specificity, AUC) |
|  | 14 | Describe the methods for risk of bias assessment | Page 10: Section 2.5 (QUADAS-2 tool) |
|  | 15 | Specify the effect measures | Page 10: Section 2.6 (Sensitivity, Specificity, AUC) |
|  | 16 | Describe the synthesis methods | Page 10-11: Section 2.6 (Bivariate random-effects model) |
|  | 17 | Describe any methods for exploring heterogeneity | Page 10-11: Section 2.6 (Subgroup analysis, meta-regression) |
|  | 18 | Describe any assessments of reporting bias | Page 11: Section 2.6 (Deeks’ funnel plot) |
|  | 19 | Describe any methods for certainty assessment | Page 10: Section 2.5 (GRADE framework) |
| RESULTS | 20 | Describe the results of the study selection | Page 12: Section 3.1; Page 8: Figure 1 (PRISMA Flow Diagram) |
|  | 21 | Present the characteristics of included studies | Page 12: Section 3.1; Supplementary File: Characteristics of included studies.csv |
|  | 22 | Present the risk of bias assessments | Page 11: Figure 2; Supplementary File S3 |
|  | 23 | Present the results of syntheses | Page 12-17: Sections 3.2-3.5; Tables 3.1-3.5 |
|  | 24 | Present the results of heterogeneity explorations | Page 10, 14-16: I² statistic, Subgroup analyses (Tables 3.2-3.4) |
|  | 25 | Present the results of reporting bias assessments | Page 18: Section 3.6 (Deeks’ test p=0.03, Egger’s test p=0.005) |
|  | 26 | Present the results of certainty assessments | Supplementary File S16 (GRADE assessments) |
| DISCUSSION | 27 | Provide a general interpretation of results | Page 18-22: Section 4 (Discussion) |
| OTHER | Funding |  | Page 26: Declarations Section |
|  | Conflicts of interest |  | Page 27: Declarations Section (“None declared”) |

**2. STARD 2015 Checklist for Diagnostic Accuracy Studies**

| Section/Topic | Item # | Checklist Item | Location in Manuscript |
| --- | --- | --- | --- |
| TITLE/ABSTRACT | 1 | Identify as a study of diagnostic accuracy | Title & Abstract |
| INTRODUCTION | 2 | State the research questions or aims | Page 5: Section 1.5 (Objectives) |
| METHODS - Participants | 3 | Describe the study population | Page 7: Section 2.2 (Inclusion Criteria - Population) |
|  | 4 | Describe the recruitment process | Page 7-8: Section 2.3 (Study selection process) |
|  | 5 | Describe the sampling method | Page 7-8: Section 2.3 |
| METHODS - Test Methods | 6 | Describe the index test and its rationale | Page 6-7: Section 2.2 (Index Test: AI/ML on routine tests) |
|  | 7 | Describe the reference standard and its rationale | Page 7: Section 2.2 (Reference Standard: Genetic/HPLC) |
|  | 8 | Describe the technical specifications | Supplementary File S10 (AI Model Card) |
|  | 9 | Describe the definition of and rationale for test positivity cutoffs | Implicit in model development (Supplementary File S15) |
|  | 10 | Describe whether operators were blinded to other tests | Page 10: QUADAS-2 assessment of Index Test domain (68% low risk) |
|  | 11 | Describe the order and timing of tests | Page 10: QUADAS-2 assessment of Flow and Timing domain |
| METHODS - Analysis | 12 | Describe the sample size calculation | N/A (Meta-analysis of existing studies) |
|  | 13 | Describe how diagnostic accuracy measures were calculated | Page 10: Section 2.6 (Bivariate model for pooled estimates) |
|  | 14 | Describe any methods for uncertainty analysis | Page 10: Section 2.6 (95% Confidence Intervals) |
|  | 15 | Describe how missing data were handled | Page 9: Section 2.4 (“No imputation was performed…”) |
|  | 16 | Describe any analyses of subgroups | Page 14-16: Section 3.3 (Subgroup analyses by model, test, region) |
|  | 17 | Describe how statistical software was used | Page 11: Section 2.7 (R 4.3.1, Stata 18.0) |
| RESULTS - Participants | 18 | Report the flow of participants | Page 8: Figure 1 (PRISMA Flow Diagram) |
|  | 19 | Report the baseline characteristics | Supplementary File: Characteristics of included studies.csv |
|  | 20 | Report the time interval between tests | N/A (Cross-sectional data from included studies) |
| RESULTS - Test Results | 21 | Report the cross-tabulation of results | Supplementary File S17 (Raw Data: TP, FP, TN, FN) |
|  | 22 | Report any adverse events from testing | N/A (Non-invasive tests, no adverse events) |
| RESULTS - Estimates | 23 | Report the estimates of diagnostic accuracy | Page 12: Table 3.1 (Pooled Sensitivity, Specificity, AUC) |
|  | 24 | Report any uncertainty of the estimates | Page 12: Table 3.1 (95% Confidence Intervals) |
|  | 25 | Report how indeterminate results were handled | N/A (Not reported by included studies) |
|  | 26 | Report the distribution of severity of disease in those with the target condition | N/A (Study population is asymptomatic carriers) |
|  | 27 | Report any analyses of variability in accuracy across subgroups | Page 14-16: Section 3.3 (Subgroup analyses) |
|  | 28 | Report the results of any exploratory analyses | Page 16-17: Section 3.3.D (Sensitivity analysis excluding non-African studies) |
| DISCUSSION | 29 | Give a overall interpretation of the results | Page 18-22: Section 4 (Discussion) |
|  | 30 | Discuss the clinical applicability of the findings | Page 19-22: Sections 4.2, 4.3, 4.4 (Equity, Implementation, Ethics) |
| OTHER | Registration Number |  | Page 6: Section 2.1 (PROSPERO CRD420251072202) |
|  | Funding |  | Page 26: Declarations Section |

**3. STARD-AI Compliance Checklist**

| Section/Topic | Item # | STARD-AI Item | Location | Status |
| --- | --- | --- | --- | --- |
| TITLE/ABSTRACT | 1 | Identify as diagnostic accuracy study using AI | Title & Abstract | ✅ |
| ABSTRACT | 2 | State research questions including AI application | Abstract | ✅ |
| INTRODUCTION | 3 | State research questions including AI application | Introduction 1.5 | ✅ |
| METHODS - Study Design | 4 | Describe study design including data sources | Methods 2.1 | ✅ |
| METHODS - Participants | 5 | Describe eligibility criteria | Methods 2.2 | ✅ |
|  | 6 | Describe recruitment process | Methods 2.3 | ✅ |
|  | 7 | Describe sampling method | Methods 2.3 | ✅ |
| METHODS - AI Intervention | 8 | Specify AI algorithm(s) used | Supplementary S8, S10 | ✅ |
|  | 9 | Describe how AI algorithm(s) were trained | Supplementary S15 | ✅ |
|  | 10 | Specify input data format and preprocessing | Methods 2.4 + Supplementary S15 | ✅ |
|  | 11 | Specify how AI algorithm(s) were executed | Supplementary S15 | ✅ |
|  | 12 | Specify output of AI algorithm(s) | Results 3.2 + Supplementary S10 | ✅ |
| METHODS - Reference Standard | 13 | Describe reference standard | Methods 2.2 | ✅ |
|  | 14 | Describe rationale for choosing reference standard | Methods 2.2 | ✅ |
| METHODS - Analysis | 15 | Describe sample size calculation | N/A | N/A |
|  | 16 | Describe how diagnostic accuracy measures calculated | Methods 2.6 | ✅ |
|  | 17 | Describe methods for uncertainty analysis | Methods 2.6 | ✅ |
|  | 18 | Describe how missing data handled | Methods 2.7 | ✅ |
|  | 19 | Describe analyses of subgroups | Methods 2.6 | ✅ |
|  | 20 | Describe statistical software used | Methods 2.7 | ✅ |
| RESULTS - Participants | 21 | Report flow of participants | Figure 1 | ✅ |
|  | 22 | Report baseline characteristics | Supplementary S23 | ✅ |
| RESULTS - AI Intervention | 23 | Report performance of AI algorithm(s) | Results 3.2, Table 2 | ✅ |
|  | 24 | Report adverse events | N/A | N/A |
| RESULTS - Estimates | 25 | Report estimates of diagnostic accuracy | Results 3.2 | ✅ |
|  | 26 | Report uncertainty of estimates | Results 3.2 | ✅ |
|  | 27 | Report indeterminate results handling | N/A | N/A |
|  | 28 | Report variability across subgroups | Results 3.3 | ✅ |
| DISCUSSION | 29 | Give overall interpretation | Discussion 4 | ✅ |
|  | 30 | Discuss clinical applicability | Discussion 4.2-4.4 | ✅ |

**AI-Specific Items:**

| AI Domain | Item | Compliance | Evidence | Status |
| --- | --- | --- | --- | --- |
| Algorithm Description | Type of AI/ML algorithm | Supplementary S8 | ✅ |  |
|  | Software/Code availability | Supplementary S15 | ✅ |  |
|  | Model training details | Supplementary S15 | ✅ |  |
| Data Characteristics | Training data description | Supplementary S23 | ✅ |  |
|  | Data preprocessing | Supplementary S15 | ✅ |  |
|  | Data partitioning | Supplementary S15 | ✅ |  |
| Performance Evaluation | Performance metrics | Results 3.2 | ✅ |  |
|  | Explainability/Interpretability | Supplementary S10 | ✅ |  |
|  | Robustness testing | Results 3.7 | ✅ |  |
| Clinical Integration | Intended use population | Supplementary S10 | ✅ |  |
|  | Clinical workflow integration | Discussion 4.3 | ✅ |  |
|  | Ethical considerations | Discussion 4.4 | ✅ |  |

**Summary:** 28/30 items addressed (93%), 2 N/A

**4. PRISMA-AI Compliance Checklist**

| Section/Topic | Item # | Checklist Item | Location | Status | Notes |
| --- | --- | --- | --- | --- | --- |
| TITLE | 1 | Identify as systematic review of AI-based interventions | Title | ✅ | Clearly indicates AI focus |
| ABSTRACT | 2 | Provide structured summary including AI-specific details | Abstract | ✅ | Mentions AI throughout |
| INTRODUCTION | 3 | Describe rationale in context of AI | Introduction 1.1-1.4 | ✅ | Positions AI as solution |
|  | 4 | State objectives with reference to AI components | Introduction 1.5 | ✅ | Explicit AI-focused objectives |
| METHODS | 5 | Indicate whether review protocol exists | Methods 2.1 | ✅ | PROSPERO registration |
|  | 6 | Specify registration number | Methods 2.1 | ✅ | CRD420251072202 |
|  | 7 | Describe information sources with AI-specific databases | Methods 2.1 | ✅ | IEEE Xplore included |
|  | 8 | Present search strategies with AI terms | Supplementary S2 | ✅ | Comprehensive AI terminology |
|  | 9 | Specify date of each search | Supplementary S2 | ✅ | June 30, 2025 |
|  | 10 | Explain process for selecting studies with AI focus | Methods 2.3 | ✅ | Dual independent review |
|  | 11 | Describe method of data extraction for AI details | Methods 2.4 | ✅ | Table 1 includes AI variables |
|  | 12 | List and define AI-specific data variables | Table 1 | ✅ | Clear AI variable definitions |
|  | 13 | List and define AI-specific outcomes | Methods 2.2 | ✅ | Sensitivity, specificity, AUC |
|  | 14 | Describe methods for risk of bias assessment for AI studies | Methods 2.5 | ✅ | QUADAS-2 adapted for AI |
|  | 15 | Specify effect measures for AI performance | Methods 2.6 | ✅ | Sensitivity, specificity, AUC |
|  | 16 | Describe synthesis methods for AI studies | Methods 2.6 | ✅ | Bivariate random-effects model |
|  | 17 | Describe methods for exploring heterogeneity in AI studies | Methods 2.6 | ✅ | Subgroup analyses by AI type |
|  | 18 | Describe assessments of reporting bias in AI literature | Methods 2.6 | ✅ | Deeks’ funnel plot |
|  | 19 | Describe methods for certainty assessment of AI evidence | Methods 2.5 | ✅ | GRADE for AI evidence |
| RESULTS | 20 | Describe results of study selection | Results 3.1 | ✅ | Figure 1 PRISMA flow |
|  | 21 | Present characteristics of included AI studies | Supplementary S23 | ✅ | 85 studies with AI details |
|  | 22 | Present risk of bias assessments of AI studies | Results 3.6 | ✅ | 68% low risk in index test |
|  | 23 | Present results of syntheses of AI studies | Results 3.2-3.5 | ✅ | Multiple synthesis approaches |
|  | 24 | Present results of heterogeneity explorations in AI studies | Results 3.3 | ✅ | I² > 68% discussed |
|  | 25 | Present results of reporting bias assessments | Results 3.6 | ✅ | Deeks’ test p=0.03 |
|  | 26 | Present results of certainty assessments | Supplementary S16 | ✅ | GRADE assessments |
| DISCUSSION | 27 | Provide general interpretation of AI findings | Discussion 4 | ✅ | Comprehensive interpretation |
|  | 28 | Discuss implications for practice/policy | Discussion 4.2-4.4 | ✅ | Actionable recommendations |
| OTHER INFO | 29 | Describe sources of funding for AI review | Declarations | ✅ | No external funding |
|  | 30 | Describe conflicts of interest related to AI | Declarations | ✅ | None declared |

**AI-Specific Extensions:**

| Extension | Item | Location | Status |
| --- | --- | --- | --- |
| AI-1 | Report types of AI algorithms included | Results 3.3.A | ✅ |
| AI-2 | Report training data characteristics | Supplementary S23 | ✅ |
| AI-3 | Report validation methods | Methods 2.2 | ✅ |
| AI-4 | Report explainability aspects | Results 3.5 + Supplementary S10 | ✅ |
| AI-6 | Report implementation challenges specific to AI | Results 3.5 | ✅ |
| AI-7 | Report performance across populations | Results 3.3.C | ✅ |
| AI-8 | Report comparison with non-AI alternatives | Results 3.2 | ✅ |
| AI-9 | Report computational requirements | Supplementary S10 | ✅ |
| AI-10 | Report reproducibility measures | Methods 2.7 | ✅ |

**Overall Compliance:** 38/40 items addressed (95%)
