# Supplementary material for: AI‐Augmented Hematological Signatures for Equitable Detection of Hereditary Hemolytic Anemia Carriers: A Global Systematic Review and Meta‐Analysis
Source: Hum Mutat. 2026 Jun 27;2026:9405486. doi: 10.1155/humu/9405486 (PMC13309745; doi:10.1155/humu/9405486)
Supplement: Supplementary file 3 — Supporting Information 3 File S2: Complete search strategies for all databases. [file HUMU-2026-9405486-s013.docx]

**File S2. Full Search Strategy**

/ ================================================

Database: Embase

Search Date: 2025-06-30

================================================ /

'premarital screening'/exp OR 'carrier detection'

'hemolytic anemia'/exp OR 'thalassemia'/exp OR 'sickle cell anemia'/exp

'artificial intelligence'/exp OR 'machine learning'/exp OR 'deep learning'/exp

'complete blood count'/exp OR 'blood smear'/exp OR 'erythrocyte sedimentation rate'/exp

#1 AND #2 AND #3 AND #4

[2010-2025]/py AND [english OR arabic]/lg

/ ================================================

Database: Scopus

Search Date: 2025-06-30

================================================ /

TITLE-ABS-KEY(("premarital screening" OR "carrier detection")

AND ("hereditary hemolytic anemia" OR thalassemia OR "sickle cell disease")

AND ("artificial intelligence" OR "machine learning" OR "deep learning")

AND ("complete blood count" OR "blood smear" OR "erythrocyte sedimentation rate"))

PUBYEAR > 2009 AND (LANGUAGE(english) OR LANGUAGE(arabic))

/ ================================================

Regional Databases (GulfBase, IMEMR, AJOL, LILACS)

Used equivalent terms in Arabic for key concepts

================================================ /
