# Supplementary material for: AI‐Augmented Hematological Signatures for Equitable Detection of Hereditary Hemolytic Anemia Carriers: A Global Systematic Review and Meta‐Analysis
Source: Hum Mutat. 2026 Jun 27;2026:9405486. doi: 10.1155/humu/9405486 (PMC13309745; doi:10.1155/humu/9405486)
Supplement: Supplementary file 6 — Supporting Information 6 File S5: R analysis code for meta‐analysis. [file HUMU-2026-9405486-s016.docx]

**S5: COMPREHENSIVE R CODE FOR META-ANALYSIS**

Title: R Analysis Script for “AI-Augmented Hematological Signatures for Equitable Detection of Hereditary Hemolytic Anemia Carriers: A Global Systematic Review and Meta-Analysis”

Author: Systematic Review Team

OSF Repository: https://osf.io/c8fhw/

DOI: 10.17605/OSF.IO/C8FHW

**1. Setup and Configuration**

# =============================================================================

# COMPREHENSIVE META-ANALYSIS SCRIPT

# PROSPERO: CRD420251072202

# =============================================================================

# Clear workspace

rm(list = ls())

gc()

# Install required packages

required_packages <- c(

"metafor", # General meta-analysis

"mada", # Diagnostic accuracy meta-analysis

"ggplot2", # Publication-quality graphics

"dplyr", # Data manipulation

"readr", # Fast data reading

"writexl", # Excel output

"rmarkdown" # Reporting

)

# Install missing packages

new_packages <- required_packages[!(required_packages %in% installed.packages()[,"Package"])]

if(length(new_packages)) install.packages(new_packages)

# Load packages

invisible(lapply(required_packages, library, character.only = TRUE))

# Set global options

options(scipen = 999) # Disable scientific notation

set.seed(123456) # Reproducibility

# Create output directories

dirs_to_create <- c("output/figures", "output/tables", "output/models")

for(dir in dirs_to_create) {

if(!dir.exists(dir)) dir.create(dir, recursive = TRUE)

}

**2. Data Import and Preprocessing**

# Load main performance data

perf_data <- read.csv("data/raw/S4_Raw_performance_data.csv", stringsAsFactors = FALSE)

# Load raw 2x2 data (TP, FP, TN, FN)

raw_2x2 <- read.csv("data/raw/S17_Raw_Data_TP_FP.csv", stringsAsFactors = FALSE)

# Load QUADAS-2 assessment

quadas_data <- read.csv("data/raw/S3_Full_QUADAS-2_assessment.csv", stringsAsFactors = FALSE)

# Merge all data

merged_data <- perf_data %>%

left_join(raw_2x2, by = "Study_ID") %>%

left_join(quadas_data, by = "Study_ID")

# Data cleaning and validation

merged_data <- merged_data %>%

mutate(

Sens_recalc = TP / (TP + FN),

Spec_recalc = TN / (TN + FP),

Data_Inconsistency = ifelse(abs(Sensitivity - Sens_recalc) > 0.05 |

abs(Specificity - Spec_recalc) > 0.05, "Yes", "No"),

Region_Group = case_when(

Region %in% c("Middle East", "North Africa") ~ "Middle East/North Africa",

Region == "Sub-Saharan Africa" ~ "Sub-Saharan Africa",

Region %in% c("South Asia", "Southeast Asia") ~ "Asia",

Region %in% c("Europe", "Americas") ~ "Europe/Americas",

TRUE ~ "Other"

)

)

# Save processed data

write.csv(merged_data, "data/processed/merged_analysis_data.csv", row.names = FALSE)

**3. Primary Meta-Analysis (Bivariate Model)**

# Fit bivariate random-effects model using Reitsma method

fit_bivariate <- reitsma(merged_data[, c("TP", "FP", "FN", "TN")],

method = "reml",

correction = 0.5)

# Extract results

results <- list(

sensitivity = c(

estimate = plogis(fit_bivariate$coefficients["mu1"]) * 100,

ci_lower = plogis(fit_bivariate$coefficients["mu1"] - 1.96 * sqrt(fit_bivariate$vcov[1,1])) * 100,

ci_upper = plogis(fit_bivariate$coefficients["mu1"] + 1.96 * sqrt(fit_bivariate$vcov[1,1])) * 100

),

specificity = c(

estimate = plogis(fit_bivariate$coefficients["mu2"]) * 100,

ci_lower = plogis(fit_bivariate$coefficients["mu2"] - 1.96 * sqrt(fit_bivariate$vcov[2,2])) * 100,

ci_upper = plogis(fit_bivariate$coefficients["mu2"] + 1.96 * sqrt(fit_bivariate$vcov[2,2])) * 100

),

auc = auc(fit_bivariate)

)

# Create summary table

summary_table <- data.frame(

Metric = c("Sensitivity", "Specificity", "AUC"),

Estimate = c(

round(results$sensitivity["estimate"], 1),

round(results$specificity["estimate"], 1),

round(results$auc[1], 3)

),

CI_95 = c(

sprintf("%.1f–%.1f", results$sensitivity["ci_lower"], results$sensitivity["ci_upper"]),

sprintf("%.1f–%.1f", results$specificity["ci_lower"], results$specificity["ci_upper"]),

sprintf("%.3f–%.3f", results$auc[2], results$auc[3])

)

)

# Save results

write.csv(summary_table, "output/tables/primary_meta_analysis_results.csv", row.names = FALSE)

**4. Subgroup Analyses**

# Subgroup by AI Model Type

model_results <- list()

models <- unique(merged_data$AI_Model_Group)

for(model in models) {

subset_data <- merged_data[merged_data$AI_Model_Group == model, ]

if(nrow(subset_data) >= 3) {

fit <- reitsma(subset_data[, c("TP", "FP", "FN", "TN")], method = "reml")

model_results[[model]] <- list(

n = nrow(subset_data),

sensitivity = plogis(fit$coefficients["mu1"]) * 100,

specificity = plogis(fit$coefficients["mu2"]) * 100

)

}

}

# Subgroup by Geographic Region

region_results <- list()

regions <- unique(merged_data$Region_Group)

for(region in regions) {

subset_data <- merged_data[merged_data$Region_Group == region, ]

if(nrow(subset_data) >= 3) {

fit <- reitsma(subset_data[, c("TP", "FP", "FN", "TN")], method = "reml")

region_results[[region]] <- list(

n = nrow(subset_data),

sensitivity = plogis(fit$coefficients["mu1"]) * 100,

specificity = plogis(fit$coefficients["mu2"]) * 100

)

}

}

# Save subgroup results

subgroup_table <- data.frame(

Subgroup = rep(c("AI Model", "Region"), each = 4),

Level = c(names(model_results), names(region_results)),

Sensitivity = c(sapply(model_results, function(x) x$sensitivity),

sapply(region_results, function(x) x$sensitivity)),

Specificity = c(sapply(model_results, function(x) x$specificity),

sapply(region_results, function(x) x$specificity))

)

write.csv(subgroup_table, "output/tables/subgroup_analysis_results.csv", row.names = FALSE)

**5. Sensitivity Analyses**

# Excluding community-based studies

community_studies <- merged_data$Study_ID[grepl("Yemen|Somalia|Sudan", merged_data$Country)]

if(length(community_studies) > 0) {

subset_data <- merged_data[!merged_data$Study_ID %in% community_studies, ]

fit <- reitsma(subset_data[, c("TP", "FP", "FN", "TN")], method = "reml")

sensitivity_results_exclude_community <- list(

n_excluded = length(community_studies),

sensitivity = plogis(fit$coefficients["mu1"]) * 100,

specificity = plogis(fit$coefficients["mu2"]) * 100

)

}

# African studies only

african_studies <- merged_data[merged_data$Region_Group == "Sub-Saharan Africa", ]

if(nrow(african_studies) >= 3) {

fit <- reitsma(african_studies[, c("TP", "FP", "FN", "TN")], method = "reml")

sensitivity_results_africa_only <- list(

n_studies = nrow(african_studies),

sensitivity = plogis(fit$coefficients["mu1"]) * 100,

specificity = plogis(fit$coefficients["mu2"]) * 100

)

}

# Save sensitivity results

sensitivity_table <- data.frame(

Analysis = c("Excluding community studies", "African studies only"),

N_Studies = c(nrow(subset_data), nrow(african_studies)),

Sensitivity = c(sensitivity_results_exclude_community$sensitivity,

sensitivity_results_africa_only$sensitivity),

Specificity = c(sensitivity_results_exclude_community$specificity,

sensitivity_results_africa_only$specificity)

)

write.csv(sensitivity_table, "output/tables/sensitivity_analysis_results.csv", row.names = FALSE)

**6. Publication Bias Assessment**

# Deeks' funnel plot asymmetry test

deeks_data <- data.frame(

log_dor = log((merged_data$TP * merged_data$TN) / (merged_data$FP * merged_data$FN)),

var_log_dor = 1/merged_data$TP + 1/merged_data$FN + 1/merged_data$TN + 1/merged_data$FP,

ess = (4 * (merged_data$TP + merged_data$FN) * (merged_data$TN + merged_data$FP)) /

(merged_data$TP + merged_data$FN + merged_data$TN + merged_data$FP)

)

deeks_test <- rma(yi = log_dor, vi = var_log_dor, mods = ~ 1/ess,

data = deeks_data, method = "REML")

# Funnel plot

library(ggplot2)

funnel_plot <- ggplot(deeks_data, aes(x = log_dor, y = 1/sqrt(ess))) +

geom_point(alpha = 0.6) +

geom_vline(xintercept = mean(deeks_data$log_dor, na.rm = TRUE),

linetype = "dashed", color = "red") +

labs(x = "Log Diagnostic Odds Ratio",

y = "Inverse Square Root of Effective Sample Size",

title = "Funnel Plot for Publication Bias Assessment",

subtitle = sprintf("Deeks' test p-value = %.3f", deeks_test$pval[2])) +

theme_minimal()

ggsave("output/figures/funnel_plot.png", funnel_plot, width = 8, height = 6, dpi = 300)

**7. Visualization**

# Forest plot

forest_data <- data.frame(

study = merged_data$Study_ID,

country = merged_data$Country,

sensitivity = merged_data$Sensitivity * 100,

lower = merged_data$Sensitivity * 100 - 1.96 *

sqrt(merged_data$Sensitivity * (1 - merged_data$Sensitivity) / merged_data$Sample_Size) * 100,

upper = merged_data$Sensitivity * 100 + 1.96 *

sqrt(merged_data$Sensitivity * (1 - merged_data$Sensitivity) / merged_data$Sample_Size) * 100

)

forest_plot <- ggplot(forest_data, aes(x = sensitivity, y = reorder(study, sensitivity))) +

geom_point() +

geom_errorbarh(aes(xmin = lower, xmax = upper), height = 0.2) +

geom_vline(xintercept = results$sensitivity["estimate"],

linetype = "dashed", color = "red") +

labs(x = "Sensitivity (%)", y = "Study",

title = "Forest Plot of Sensitivity Estimates") +

theme_minimal()

ggsave("output/figures/forest_plot.png", forest_plot, width = 10, height = 12, dpi = 300)

# SROC curve

sroc_curve <- plot(sroc(fit_bivariate),

main = "Summary ROC Curve",

xlab = "False Positive Rate (1 - Specificity)",

ylab = "True Positive Rate (Sensitivity)")

png("output/figures/sroc_curve.png", width = 800, height = 600)

plot(sroc(fit_bivariate),

main = "SROC Curve for AI-Augmented HHA Screening",

xlab = "1 - Specificity",

ylab = "Sensitivity")

dev.off()

**8. Session Information**

# Save session info

sink("output/session_info.txt")

cat("META-ANALYSIS SESSION INFORMATION\n")

cat("=================================\n\n")

cat("Analysis Date:", Sys.Date(), "\n")

cat("R Version:", R.version$version.string, "\n\n")

cat("Packages Used:\n")

print(sessionInfo()$otherPkgs)

sink()
