# Supplementary material for: AI‐Augmented Hematological Signatures for Equitable Detection of Hereditary Hemolytic Anemia Carriers: A Global Systematic Review and Meta‐Analysis
Source: Hum Mutat. 2026 Jun 27;2026:9405486. doi: 10.1155/humu/9405486 (PMC13309745; doi:10.1155/humu/9405486)
Supplement: Supplementary file 7 — Supporting Information 7 File S6: Sensitivity analysis for protocol amendment. [file HUMU-2026-9405486-s017.docx]

**File S6: Sensitivity Analysis for Protocol Amendment**

| Analysis Cohort | n Studies | Sensitivity (95% CI) | Specificity (95% CI) | AUC (95% CI) | Cost Savings/Person (95% CI) |
| --- | --- | --- | --- | --- | --- |
| Original Protocol | 82 | 92.8% (91.3-94.1) | 91.5% (89.7-93.0) | 0.93 (0.91-0.95) | $8.50 ($5.20-12.30) |
| + Community Studies | 85 | 92.6% (91.1-93.9) | 91.2% (89.4-92.8) | 0.93 (0.91-0.95) | $8.20 ($4.90-11.80) |
| Difference (95% CI) | - | -0.2% (-0.7-0.3) | -0.3% (-0.9-0.3) | 0.00 (-0.01-0.01) | -$0.30 (-$0.80-0.20) |

**Key Findings Statement:** “Inclusion of community-based studies did not significantly alter diagnostic accuracy(Δsensitivity: -0.2% [95% CI: -0.7-0.3], p=0.41; Δspecificity: -0.3% [-0.9-0.3], p=0.38) but reduced cost savings by $0.30/person (95% CI: -$0.80-0.20) due to higher point-of-care implementation expenses in resource-constrained settings.”
