# Supplementary material for: AI‐Augmented Hematological Signatures for Equitable Detection of Hereditary Hemolytic Anemia Carriers: A Global Systematic Review and Meta‐Analysis
Source: Hum Mutat. 2026 Jun 27;2026:9405486. doi: 10.1155/humu/9405486 (PMC13309745; doi:10.1155/humu/9405486)
Supplement: Supplementary file 8 — Supporting Information 8 File S7: Algorithmic bias dataset. [file HUMU-2026-9405486-s018.docx]

**File S7: Algorithmic Bias Analysis (African Studies Performance)**

| Study_ID | Region | Genetic_Variant | Sensitivity(%) | Specificity(%) |
| --- | --- | --- | --- | --- |
| Study7 | Africa | HbSS | 89.1 | 90.2 |
| Study11 | Africa | HbSC | 80.5 | 87.3 |
| Study16 | Africa | HbSβ | 83.7 | 85.9 |
| Study29 | Africa | HbCC | 84.9 | 82.1 |
| Study36 | Africa | HbSβ | 81.3 | 80.5 |
| Study41 | Africa | HbSS | 82.1 | 83.7 |
| Study51 | Africa | HbSC | 80.2 | 84.6 |
| Study54 | Africa | HbSS | 77.8 | 86.3 |
| Study56 | Africa | HbCC | 83.1 | 80.9 |
| Study58 | Africa | HbSβ | 84.7 | 85.1 |
| Study59 | Africa | HbSS | 82.6 | 81.4 |
| Study69 | Africa | HbSC | 79.8 | 83.2 |
| Study74 | Africa | HbCC | 85.3 | 82.7 |

**Summary of Performance Disparities:**

Overall African Sensitivity: 82.4% (95% CI: 79.8-84.7)

Overall African Specificity: 84.1% (95% CI: 81.6-86.3)

Compared to Global Pooled: ΔSensitivity: -10.4% (p<0.001), ΔSpecificity: -7.4% (p=0.008)

Worst Performing Variant: HbSC (Sensitivity: 80.2%, Specificity: 84.6%)

Best Performing Variant: HbCC (Sensitivity: 85.3%, Specificity: 82.7%)
