# Supplementary material for: AI‐Augmented Hematological Signatures for Equitable Detection of Hereditary Hemolytic Anemia Carriers: A Global Systematic Review and Meta‐Analysis
Source: Hum Mutat. 2026 Jun 27;2026:9405486. doi: 10.1155/humu/9405486 (PMC13309745; doi:10.1155/humu/9405486)
Supplement: Supplementary file 9 — Supporting Information 9 File S8: Classification of AI model types. [file HUMU-2026-9405486-s019.docx]

**File S8: Classification of AI Model Types**

**1. Deep Learning (DL) Models**

Algorithms: Convolutional Neural Networks (CNN), Recurrent Neural Networks (RNN), Transformers, Autoencoders

Primary Use:Image-based analysis of blood smears, morphological pattern recognition

Training Data Requirements:≥1,000 annotated images per class

Hardware Requirements:GPU recommended (≥8GB VRAM)

Examples in Review:

CNN for blood smear classification (n=38 studies)

RNN for sequential hematological parameter analysis (n=12)

Vision Transformers for multi-resolution analysis (n=7)

**2. Explainable AI (XAI) Models**

Algorithms: SHAP (SHapley Additive exPlanations), LIME (Local Interpretable Model-agnostic Explanations), Decision Trees, Rule-based systems

Primary Use:Clinically interpretable decisions, regulatory compliance

Transparency Score:High (feature importance visualization)

Examples in Review:

SHAP-based thalassemia prediction (n=15)

LIME-interpretable decision trees (n=11)

Integrated Gradients for CNN explanations (n=8)

**3. Federated Learning (FL) Models**

Framework: PySyft, TensorFlow Federated, Flower, OpenFL

Primary Use:Multi-institutional collaboration without data sharing

Privacy Features:Differential privacy, secure aggregation

Examples in Review:

Multi-country FL for HHA detection (n=9)

Hospital network FL with privacy preservation (n=6)

**4. Edge AI Models**

Hardware: Mobile devices, Raspberry Pi, NVIDIA Jetson, Google Coral

Primary Use:Point-of-care screening in resource-limited settings

Optimization:Model quantization, pruning, TensorFlow Lite

Examples in Review:

Smartphone-based microscopy AI (n=14)

Solar-powered edge devices (n=7)

Offline-capable screening tools (n=9)

**5. Ensemble Methods**

Techniques: Stacking, Voting, Bagging, Boosting

Primary Use:Improved generalization, reduced variance

Components:Typically 3-7 base models

Examples in Review:

Random Forest + XGBoost ensemble (n=18)

Deep learning ensemble with multiple architectures (n=12)

**6. Hybrid Models**

Combinations: DL + XAI, FL + Edge AI, Traditional ML + DL

Primary Use:Balancing performance with practical constraints

Examples in Review:

CNN with SHAP explanations (n=10)

Federated edge learning (n=5)
