# Supplementary material for: AI‐Augmented Hematological Signatures for Equitable Detection of Hereditary Hemolytic Anemia Carriers: A Global Systematic Review and Meta‐Analysis
Source: Hum Mutat. 2026 Jun 27;2026:9405486. doi: 10.1155/humu/9405486 (PMC13309745; doi:10.1155/humu/9405486)
Supplement: Supplementary file 10 — Supporting Information 10 File S9: Regional cost breakdown. [file HUMU-2026-9405486-s020.docx]

**File S9: Regional Cost Breakdown**

| Region | Device_Cost_Min_USD | Device_Cost_Max_USD | Training_Cost_USD | Avg_Savings_USD | Key Barriers |
| --- | --- | --- | --- | --- | --- |
| Sub-Saharan Africa | 120 | 300 | 50-100 | 5.20 | Power outages (76%), internet instability, technician shortage |
| Middle East | 80 | 200 | 30-80 | 9.80 | Technician training (35%), regulatory approval, cultural adaptation |
| Europe/Americas | 200 | 500 | 100-200 | 12.30 | Regulatory compliance (60%), integration costs, reimbursement policies |
| South Asia | 90 | 250 | 40-90 | 7.10 | Infrastructure gaps (55%), maintenance costs, user training |

**Cost-Effectiveness Analysis:**

Break-even Screening Volume:

Africa: 1,250 individuals (at $5.20 savings/person)

Middle East: 850 individuals (at $9.80 savings/person)

Europe/Americas: 700 individuals (at $12.30 savings/person)

ROI Timeline: 18-24 months for high-volume settings (>2,000 screens/year)

Most Cost-Effective Configuration: CBC + Mobile AI ($3.50/test, 92.3% sensitivity)
