# Supplementary material for: AI‐Augmented Hematological Signatures for Equitable Detection of Hereditary Hemolytic Anemia Carriers: A Global Systematic Review and Meta‐Analysis
Source: Hum Mutat. 2026 Jun 27;2026:9405486. doi: 10.1155/humu/9405486 (PMC13309745; doi:10.1155/humu/9405486)
Supplement: Supplementary file 11 — Supporting Information 11 File S10: Algorithmic bias audit and fairness certification report for HbS/HbC variants. [file HUMU-2026-9405486-s021.docx]

# File S10: Algorithmic Bias Audit & Fairness Certification Report for HbS/HbC Variants

## EXECUTIVE SUMMARY

- **Report ID**: AI-BIAS-AUDIT-2025-HHA-001
- **Date**: 15 January 2025
- **Audit Period**: Studies published 2010-2025
- **Total Studies Audited**: 85 studies (133,498 participants)
- **Primary Focus**: HbS and HbC variant performance disparities
- **Audit Team**: Independent AI Ethics Review Board

## 1. OVERVIEW OF BIAS AUDIT

### 1.1 Audit Objectives

1. Quantify performance disparities for African genetic variants (HbS/HbC)
2. Identify sources of algorithmic bias in HHA screening AI
3. Provide certification of fairness for regulatory approval
4. Recommend bias mitigation strategies

### 1.2 Audit Standards

- EU AI Act (High-risk medical device requirements)
- FDA AI/ML Action Plan (Bias monitoring requirements)
- ISO/IEC 24027:2021 (Bias in AI systems)
- WHO Ethical Guidelines for AI in Health

## 2. DATA REPRESENTATION ANALYSIS

### 2.1 Geographic Distribution in Training Data

| Region | Studies | Participants | % of Total | Global Burden |
| --- | --- | --- | --- | --- |
| Middle East | 29 | 61,230 | 45.9% | 25% |
| South Asia | 25 | 42,180 | 31.6% | 30% |
| Europe/Americas | 18 | 14,858 | 11.1% | 5% |
| Sub-Saharan Africa | 13 | 15,230 | 11.4% | 40% |

**Finding**: Severe underrepresentation - Africa contributes 40% of global HHA burden but only 11.4% of training data.

### 2.2 Genetic Variant Distribution

| Variant | Studies | Samples | Performance Data Available |
| --- | --- | --- | --- |
| HbAA (Normal) | 85 | 113,101 | Complete |
| β-thalassemia | 78 | 8,420 | Complete |
| HbS (African) | 23 | 1,850 | Partial (67% studies) |
| HbC (African) | 17 | 920 | Limited (43% studies) |
| HbE (Asian) | 31 | 3,240 | Complete |
| Other variants | 42 | 1,967 | Variable |

**Critical Gap**: HbS/HbC variants have limited performance reporting despite high prevalence.

## 3. PERFORMANCE DISPARITIES QUANTIFICATION

### 3.1 Overall Performance by Variant

VARIANT SENSITIVITY SPECIFICITY AUC Δ vs Overall
--------------- ------------ ------------ -------- ------------
Overall 92.8% 91.5% 0.93 Reference
β-thalassemia 94.2% 92.1% 0.95 +1.4%
HbE 93.5% 91.8% 0.94 +0.7%
HbS 86.5% 89.2% 0.87 -6.3%
HbC 84.8% 88.7% 0.86 -7.0%
Mixed variants 90.1% 90.5% 0.90 -2.7%

### 3.2 Geographic Performance Disparities

| Region | Sensitivity | Specificity | AUC | HbS/HbC Samples |
| --- | --- | --- | --- | --- |
| Middle East | 94.8% | 93.2% | 0.95 | 420 (0.7%) |
| South Asia | 92.3% | 90.7% | 0.93 | 310 (0.7%) |
| Europe/Americas | 93.5% | 92.8% | 0.94 | 280 (1.9%) |
| Sub-Saharan Africa | 86.5% | 89.2% | 0.87 | 1,740 (11.4%) |

**Key Finding**: Inverse relationship - regions with most HbS/HbC cases have lowest performance.

### 3.3 Worst-Performing Subgroups

RANK SUBGROUP SENSITIVITY STUDY ID COUNTRY
---- ----------------------- ------------ --------- --------
1 HbSS (Homozygous) 77.8% Study57 Mali
2 HbSC (Compound) 79.2% Study54 Yemen
3 HbS/β-thal 81.3% Study13 Sudan
4 HbCC 82.1% Study36 Chad
5 HbAS (Trait) 86.5% Study7 Nigeria

## 4. SOURCE ANALYSIS OF BIAS

### 4.1 Data-Level Biases

| Bias Type | Severity | Impact | Evidence |
| --- | --- | --- | --- |
| Sample Imbalance | High | 8.2% performance drop | 12% African data vs 40% burden |
| Annotation Quality | Medium | 3-5% variance | Inter-rater variability κ=0.65-0.78 |
| Data Augmentation | Low | Minimal | Limited augmentation for rare variants |
| Label Noise | Medium | 2-4% error | Confirmatory testing inconsistencies |

### 4.2 Algorithm-Level Biases

| Algorithm Type | HbS Sensitivity | Bias Score (1-10) | Primary Issue |
| --- | --- | --- | --- |
| Deep Learning | 85.2% | 7.2 | Overfitting to majority variants |
| Random Forest | 87.1% | 6.8 | Feature importance skewed |
| XAI Models | 88.3% | 6.1 | Better but still biased |
| Federated Learning | 89.7% | 5.4 | Best for diverse data |
| Edge AI | 82.4% | 8.1 | Limited model capacity |

### 4.3 Feature Importance Disparities

FEATURE IMPORTANCE (Overall) IMPORTANCE (HbS) Δ
---------------- --------------------- ----------------- -----
MCV 35.2% 28.7% -6.5%
MCH 28.8% 31.2% +2.4%
RDW 15.7% 22.5% +6.8%
Hb Concentration 12.3% 10.1% -2.2%
RBC Morphology 8.0% 7.5% -0.5%

**Finding**: HbS detection relies more on RDW and less on MCV compared to other variants.

## 5. FAIRNESS METRICS CERTIFICATION

### 5.1 Demographic Parity

| Metric | Target | Achieved | Status |
| --- | --- | --- | --- |
| Sensitivity Gap | <5% | 8.2% | ❌ FAIL |
| Specificity Gap | <3% | 2.3% | ✅ PASS |
| AUC Gap | <0.05 | 0.06 | ❌ FAIL |
| FPR Parity | <2% | 3.1% | ❌ FAIL |

### 5.2 Equalized Odds

| Subgroup | TPR Parity | FPR Parity | Status |
| --- | --- | --- | --- |
| HbS vs Overall | -6.3% | +1.8% | ❌ FAIL |
| African vs Non-African | -5.6% | +1.2% | ❌ FAIL |
| Low-resource vs High-resource | -4.8% | +2.1% | ❌ FAIL |

### 5.3 Predictive Parity

| Threshold | PPV Gap | NPV Gap | Status |
| --- | --- | --- | --- |
| 0.5 | -8.7% | -1.2% | ❌ FAIL |
| 0.7 | -6.3% | -0.8% | ❌ FAIL |
| 0.9 | -3.2% | -0.3% | ⚠️ MARGINAL |

## 6. MITIGATION STRATEGIES IMPLEMENTED

### 6.1 Data-Level Interventions

| Strategy | Studies | Impact on HbS Sensitivity |
| --- | --- | --- |
| Oversampling | 8 | +3.2% |
| Synthetic Data | 5 | +2.1% |
| Transfer Learning | 12 | +4.7% |
| Federated Learning | 9 | +6.8% |

### 6.2 Algorithm-Level Interventions

| Intervention | Effectiveness | Implementation Cost |
| --- | --- | --- |
| Fairness-aware Loss | +4.2% | Medium |
| Adversarial Debiasing | +5.1% | High |
| Post-processing | +2.8% | Low |
| Ensemble with Fair Models | +3.9% | Medium |

### 6.3 Best Practice Example: Study 26 (Cameroon)

- **Approach**: Federated learning across 5 African sites
- **HbS Sensitivity**: 91.2% (+12.5% improvement)
- **Key Features**: Local data retention, regular model updates
- **Certification**: ISO 24027 compliant

## 7. CERTIFICATION RECOMMENDATIONS

### 7.1 Conditional Certification

Based on audit findings, we recommend **CONDITIONAL CERTIFICATION** with the following requirements:

1. **Mandatory Bias Monitoring**: Quarterly performance reports by variant
2. **African Data Quota**: Minimum 30% African samples in training data
3. **Fairness Threshold**: HbS sensitivity ≥90% within 12 months
4. **Transparency**: Public model cards with subgroup performance

### 7.2 Certification Levels

| Level | Requirements | Current Status |
| --- | --- | --- |
| Bronze | Awareness of bias | ✅ ACHIEVED |
| Silver | Mitigation plans | ⚠️ PARTIAL |
| Gold | Equity in deployment | ❌ NOT ACHIEVED |

**Current Overall Certification**: SILVER (Conditional)

## 8. ACTIONABLE RECOMMENDATIONS

### 8.1 Immediate Actions (0-3 Months)

1. **African Data Collection Initiative**: Target 10,000 new African samples
2. **Bias Dashboard**: Real-time monitoring of subgroup performance
3. **Algorithm Retraining**: Prioritize HbS/HbC variant performance

### 8.2 Medium-Term Actions (3-12 Months)

1. **Federated Learning Hubs**: Establish in Nigeria, Ghana, Kenya
2. **Fairness Certification Program**: ISO 24027 implementation
3. **Regulatory Engagement**: FDA/CE bias audit requirements

### 8.3 Long-Term Actions (12-24 Months)

1. **Global Equity Standard**: WHO guidelines for AI in genetic screening
2. **Open Benchmark Dataset**: Curated diverse HHA dataset
3. **Continuous Monitoring**: Automated bias detection systems

## 9. REGULATORY COMPLIANCE CHECKLIST

### 9.1 EU AI Act (High-Risk Medical Device)

- [✅] Risk management system
- [✅] Data quality management
- [⚠️] Bias monitoring and mitigation
- [❌] Performance across subgroups
- [✅] Human oversight provisions
- [⚠️] Transparency requirements

### 9.2 FDA AI/ML Action Plan

- [✅] Predetermined change control plan
- [⚠️] Bias identification and mitigation
- [✅] Real-world performance monitoring
- [❌] Subgroup analysis reporting
- [✅] Device modification protocols

## 10. CONCLUSION AND CERTIFICATION

### 10.1 Summary Findings

1. **Significant Bias Exists**: 8.2% sensitivity gap for African variants
2. **Data Representation Crisis**: Only 12% African data vs 40% burden
3. **Mitigation Possible**: Federated learning shows +12.5% improvement potential
4. **Regulatory Action Needed**: Current models fail fairness criteria

### 10.2 Final Certification

- **CERTIFICATION STATUS**: CONDITIONALLY CERTIFIED
- **VALID UNTIL**: 30 June 2026
- **RENEWAL REQUIREMENTS**: Demonstrate HbS sensitivity ≥90%

### 10.3 Contact for Recertification

- **AI Ethics Review Board**
- **Email**: ethics@ai-hha-review.org
- **Audit Reference**: AI-BIAS-AUDIT-2025-HHA-001

## APPENDICES

- Appendix A: Detailed performance metrics by study
- Appendix B: Bias mitigation implementation guide
- Appendix C: Regulatory compliance templates
- Appendix D: Federated learning protocol specifications
