# Supplementary material for: AI‐Augmented Hematological Signatures for Equitable Detection of Hereditary Hemolytic Anemia Carriers: A Global Systematic Review and Meta‐Analysis
Source: Hum Mutat. 2026 Jun 27;2026:9405486. doi: 10.1155/humu/9405486 (PMC13309745; doi:10.1155/humu/9405486)
Supplement: Supplementary file 12 — Supporting Information 12 File S11: Sensitivity analysis of African studies. [file HUMU-2026-9405486-s005.docx]

**File S11: African Studies Sensitivity Analysis**

| Analysis Cohort | n Studies | Sensitivity(%) | Specificity(%) | AUC | Key Implications |
| --- | --- | --- | --- | --- | --- |
| Full Analysis (All 85 studies) | 85 | 92.8 | 91.5 | 0.93 | Global estimate, includes diverse populations |
| Excluding Non-African Studies | 13 | 89.7 | 88.3 | 0.89 | Africa-specific performance, reveals representation gap |
| Difference | - | -3.1% | -3.2% | -0.04 | Statistically significant (p=0.008) |

**Regional Breakdown within Africa:**

| African Sub-region | Studies | Sensitivity(%) | Specificity(%) | Key Challenges |
| --- | --- | --- | --- | --- |
| West Africa | 5 | 87.2 | 86.5 | Infrastructure, power stability |
| East Africa | 4 | 88.1 | 87.3 | Technician training, maintenance |
| North Africa | 3 | 91.4 | 90.2 | Better infrastructure, higher costs |
| Central Africa | 1 | 84.7 | 85.1 | Severe resource constraints |

**Impact on Public Health:**

Missed Carriers: 3.1% increase in Africa-specific analysis

Annual Impact (per million screened): ~31,000 additional missed carriers

Potential Affected Births: ~7,750 annually (Hardy-Weinberg assumptions)

Economic Impact: $3.10 reduced savings per person screened
