# Supplementary material for: AI‐Augmented Hematological Signatures for Equitable Detection of Hereditary Hemolytic Anemia Carriers: A Global Systematic Review and Meta‐Analysis
Source: Hum Mutat. 2026 Jun 27;2026:9405486. doi: 10.1155/humu/9405486 (PMC13309745; doi:10.1155/humu/9405486)
Supplement: Supplementary file 13 — Supporting Information 13 File S12: FAIR data and ethical compliance checklists. [file HUMU-2026-9405486-s007.docx]

**File S12: FAIR Data and Ethical Compliance Checklists**

**1. FAIR Data Compliance Checklist**

| Principle | Check | Status | Evidence |
| --- | --- | --- | --- |
| **Findable** | Unique persistent identifier | ✅ | DOI 10.17605/OSF.IO/C8FHW |
|  | Rich metadata | ✅ | OSF repository description |
|  | Clear data license | ✅ | CC-BY 4.0 (OSF default) |
| **Accessible** | Standard communication protocol | ✅ | HTTPS/API access |
|  | Authentication not required | ✅ | Public repository |
|  | Metadata persists after data deletion | ✅ | OSF preservation policy |
| **Interoperable** | Machine-readable format | ✅ | CSV/JSON/R/Python files |
|  | Standard vocabularies | ✅ | MeSH terms in manuscript |
|  | Qualified references | ✅ | Linked publications in OSF |
| **Reusable** | Detailed data provenance | ✅ | README in OSF with processing history |
|  | Domain-relevant standards | ✅ | PRISMA/STARD/GRADE compliance |
|  | Clear usage license | ✅ | CC-BY 4.0 in OSF |

**FAIR Implementation Details:**

Data Repository: Open Science Framework (OSF)

Access URL: https://osf.io/c8fhw/

License: Creative Commons Attribution 4.0 International

Format Standards: CSV for data, R/Python for code, DOCX/PDF for documentation

Metadata Schema: Dublin Core + domain-specific extensions

Version Control: Git integration with OSF

**2. Ethical Compliance Checklist**

| Area | Requirement | Status | Location |
| --- | --- | --- | --- |
| **Data Privacy** | De-identified participant data | ✅ | Methods 2.4 (No personal IDs) |
|  | Secure repository | ✅ | OSF encrypted storage |
| **Consent** | Cultural adaptation described | ✅ | Discussion 4.4 + S10 |
|  | IRB approval statement | ✅ | Declarations: “Not required” |
| **Algorithmic Bias** | Bias audits performed | ✅ | Results 3.5 + Supplementary S7 |
|  | Mitigation strategies proposed | ✅ | Discussion 4.2 + Recommendations |
| **Conflict of Interest** | Commercial interests disclosed | ✅ | Declarations: “None declared” |
| **Access Equity** | Solutions for resource-limited settings | ✅ | Discussion 4.2 + Table 4 |

**Ethical Framework Implementation:**

Informed Consent: Tiered approach (written/verbal/witnessed) based on literacy

Data Protection: Federated learning for privacy, encryption for storage/transmission

Bias Management: Regular audits, oversampling of underrepresented groups

Benefit Sharing: Open access publication, code/data sharing, capacity building

Accountability: Clear responsibility chain, audit trail, grievance mechanisms
