# Supplementary material for: AI‐Augmented Hematological Signatures for Equitable Detection of Hereditary Hemolytic Anemia Carriers: A Global Systematic Review and Meta‐Analysis
Source: Hum Mutat. 2026 Jun 27;2026:9405486. doi: 10.1155/humu/9405486 (PMC13309745; doi:10.1155/humu/9405486)
Supplement: Supplementary file 14 — Supporting Information 14 File S13: Full PROSPERO protocol. [file HUMU-2026-9405486-s006.pdf]

Routine Laboratory Tests and Artificial Intelligence in Pre-Marital Screening: A Systematic Review for the Prevention of Hereditary Hemolytic Anemia

Naif Ali, Hana Ali

Citation
Naif Ali, Hana Ali. Routine Laboratory Tests and Artificial Intelligence in Pre-Marital Screening: A Systematic Review for the Prevention of Hereditary Hemolytic Anemia. PROSPERO 2025 CRD420251072202. Available from https://www.crd.york.ac.uk/PROSPERO/view/CRD420251072202.

PREVIEW

REVIEW TITLE AND BASIC DETAILS

Review title
Routine Laboratory Tests and Artificial Intelligence in Pre-Marital Screening: A Systematic Review for the Prevention of Hereditary Hemolytic Anemia

Condition or domain being studied
Population-based healthcare; Non-Pharmacological Interventions; Screening Procedure; Clinical Outcome

Rationale for the review
This systematic review addresses a critical gap in pre-marital screening programs for hereditary hemolytic anemias (e.g., thalassemia, sickle cell disease). While routine lab tests (CBC, blood smear, ESR) are widely used for initial carrier detection, their interpretation relies heavily on manual analysis, which may lack sensitivity or scalability. The integration of artificial intelligence (AI) offers transformative potential to: 1. Enhance accuracy by detecting subtle patterns in routine test data that humans might miss. 2. Improve accessibility in resource-limited settings where advanced diagnostics (e.g., genetic testing) are unavailable. 3. Standardize screening across diverse populations, reducing variability in test interpretation. Despite promising pilot studies, no comprehensive review has evaluated the real-world effectiveness of AI in this context or synthesized implementation challenges (e.g., data diversity, infrastructure needs). This review will: - Provide evidence on whether AI-augmented routine tests can replace or triage costly confirmatory testing. - Guide policymakers on feasible AI integration into existing screening programs. - Identify research gaps to prioritize future AI model development. By bridging these gaps, the review aims to optimize prevention strategies for hereditary anemias globally, aligning with WHO goals for genetic disease reduction. Key Additions to Existing Knowledge: - First systematic evaluation of AI + routine tests for pre-marital carrier screening. - Practical insights into barriers/facilitators for AI adoption in public health programs. - Evidence-based recommendations to update screening guidelines.

Review objectives
Primary Objective: To evaluate the diagnostic accuracy of AI-assisted routine laboratory tests (CBC, blood smear, ESR) for detecting carriers of hereditary hemolytic anemia in pre-marital screening programs. Secondary Objectives: 1. To compare the performance of AI models versus conventional interpretation methods. 2. To identify implementation challenges (e.g., cost, infrastructure) of AI integration. 3. To assess

clinical utility (e.g., reduction in unnecessary confirmatory testing). Research Questions: 1. How does AI improve sensitivity/specificity of routine tests for carrier detection? 2. What are the key barriers to adopting AI in real-world screening programs? 3. Can AI reduce disparities in access to effective screening?

Keywords
Premarital screening; Hereditary hemolytic anemia; Artificial intelligence; Routine laboratory tests; Thalassemia; Sickle cell disease; Complete blood count CBC; Peripheral blood smear; Erythrocyte Sedimentation Rate ESR; Machine learning; Diagnostic accuracy; Sensitivity and specificity; Carrier detection; Predictive modeling; Screening programs; Public health; Resource-limited settings; Cost-effectiveness; Neural networks; Clinical decision support systems

Country
Saudi Arabia; Iran (Islamic Republic of); Greece; India; United Arab Emirates; Türkiye; Pakistan; Bangladesh; Malaysia; United States of America; United Kingdom; Italy; Yemen

ELIGIBILITY CRITERIA

Population

Included
• Asymptomatic individuals undergoing premarital screening • Couples of reproductive age (typically 18-45 years) • Populations with high prevalence of hereditary hemolytic anemias (thalassemia, sickle cell disease) • Studies using routine blood tests (CBC, blood smear, ESR) as initial screening.

Broaden the definition of the "Population" to explicitly include individuals undergoing systematic carrier detection in community and reproductive health settings, even outside of formal, government-mandated premarital screening programs.

Excluded
• Symptomatic patients or those with diagnosed hemolytic anemia • Studies focusing exclusively on genetic testing without routine lab correlates • Non-reproductive age groups (pediatric/elderly populations) • General population screenings without premarital/preconception context

Intervention(s) or exposure(s)

Included
Artificial intelligence; Machine learning; Laboratory procedure; Urine Blood Test; Diagnostic Procedure

• AI/ML models analyzing routine lab tests (CBC, blood smear, ESR) for carrier detection • Hybrid approaches combining AI with manual review • Studies validating AI tools in real-world screening settings • Both supervised and unsupervised learning models

Excluded
• AI systems using only advanced tests (e.g., HPLC, genetic sequencing) without routine lab inputs • Non-AI decision tools (e.g., simple rule-based algorithms) • Theoretical models without clinical validation • AI applications focused solely on treatment/disease management

Comparator(s) or control(s)

Included
PICO tags selected: Diagnostic Procedure; Laboratory Data Interpretation; Hematologic test; Screening Procedure

• Non-AI methods of interpreting routine lab tests (CBC, blood smear, ESR)• Manual hematologist/clinician interpretation• Conventional automated analyzer results without machine learning• Rule-based decision algorithms (non-ML computerized systems)

Excluded

• Advanced diagnostic methods (HPLC, genetic testing) without routine lab correlation• AI-assisted interpretation (even if partial)• Theoretical/untested interpretation approaches• Studies where comparator methodology is unspecified

Study design

Only nonrandomized study types will be included.

Included

• Diagnostic accuracy studies (prospective/retrospective) • Clinical validation studies of AI models • Comparative studies (AI vs. conventional interpretation) • Implementation research on AI-integrated screening programs • High-quality cohort studies with outcome data

Excluded

• Case reports or case series (<10 participants) • Review articles (systematic reviews will be hand-searched) • Conference abstracts without full methodology • Opinion pieces/editorials without original data • Animal or pure in vitro studies

Context

This systematic review evaluates the integration of artificial intelligence (AI) with routine laboratory tests (complete blood count, peripheral blood smear, erythrocyte sedimentation rate) in global pre-marital screening programs for hereditary hemolytic anemias (thalassemia, sickle cell disease). Why this matters: 1. Public health urgency: 5-7% of global populations are carriers of hemoglobinopathies, with high prevalence in Mediterranean, Middle Eastern, and South Asian regions. 2. Diagnostic gap: Current programs rely on manual interpretation of basic tests, risking missed carriers due to subjective analysis. 3. AI opportunity: Machine learning can enhance accuracy, standardize results, and improve accessibility in resource-limited settings. Unique focus: First synthesis of both diagnostic performance and implementation challenges of AI-augmented screening.

SIMILAR REVIEWS

Check for similar records already in PROSPERO

PROSPERO identified a number of existing PROSPERO records that were similar to this one (last check made on 12 June 2025). These are shown below along with the reasons given by that the review team for the reviews being different and/or proceeding.

- Artificial Intelligence-Driven Prediction of Free Flap Failure in Microsurgery A Systematic Review and Meta-Analysis [published 7 May 2025] [CRD420251047313]. The review was judged **not to be similar**
- AI-based detection and measurement of marginal bone loss around dental implants in periapical radiographs - a systematic review. [published 14 March 2025] [CRD420251009693]. The review was judged **not to be similar**
- Screening Mammography and Artificial Intelligence: A Comprehensive Systematic Review [published 17 January 2025] [CRD42025634360]. The review was judged **not to be similar**
- Comprehensive Systematic Review and Meta-Analysis: Evaluating Artificial Intelligence (AI) Effectiveness and Integration Obstacles within Anesthesiology [published 6 December 2024] [CRD42024618865]. The review was judged **not to be similar**

- Real-World Clinical Validation of AI-Powered Clinical Decision Support Systems in Hospitals: A Systematic Review of Their Impact on Patient Safety and Quality of Care [published 2 June 2025] [CRD420251038285]. The review was judged **not to be similar**
- The Role of Artificial Intelligence and Machine Learning in the Diagnosis and Management of Temporomandibular Disorders: A Systematic Review [published 17 April 2025] [CRD420251035080]. The review was judged **not to be similar**
- Artificial Intelligence in Acute Stroke Management: A Systematic Review & Meta-analysis [published 24 February 2025] [CRD420250652390]. The review was judged **not to be similar**
- Artificial Intelligence in Temporomandibular Disorders: Part I – Diagnostic Accuracy and Clinical Utility (Systematic Review and Meta-analysis); Part II – AI Treatment Planning, Preoperative Decision Support, and Postoperative Outcome Prediction [published 7 May 2025] [CRD420251047311]. The review was judged **not to be similar**
- Artificial Intelligence in Cerebrovascular Diseases: A Comprehensive Systematic Review of Revolutionary Innovations in Risk Prediction, Diagnosis, and Management [published 27 January 2025] [CRD42025638869]. The review was judged **not to be similar**

TIMELINE OF THE REVIEW

Date of first submission to PROSPERO
12 June 2025

Review timeline
Start date: 12 June 2025. End date: 31 December 2025.

Date of registration in PROSPERO
12 June 2025

AVAILABILITY OF FULL PROTOCOL

Availability of full protocol
A full protocol has been written but is not available because:

Protocol is being finalized for institutional approval prior to publication.

SEARCHING AND SCREENING

Search for unpublished studies
Only published studies will be sought.

Main bibliographic databases that will be searched
The main databases to be searched are *CENTRAL - Cochrane Central Register of Controlled Trials*, *CINAHL - Cumulative Index to Nursing and Allied Health Literature*, *Embase.com*, *LILACS - Latin American and Caribbean Health Sciences Literature*, *MEDLINE*, *PubMed* and *Scopus*.

Other important or specialist databases that will be searched
✔ Other specialist databases: "GulfBase" or "IMEMR" (for Middle Eastern screening programs)

LILACS (If including Latin American studies) IEEE Xplore (Key for AI/ML algorithms in medicine)

African Journals Online (AJOL) and Latin American & Caribbean Health Sciences Literature (LILACS).

Search language restrictions

The review will only include studies published in English, Arabic, Spanish and French.

Search date restrictions

Databases will be searched for articles published from 1 January 2010, there are no search end date restrictions.

Other methods of identifying studies

Other studies will be identified by: *contacting authors or experts*, *looking through all the articles that cite the papers included in the review ("snowballing")*, *reference list checking*, *searching conference proceedings* and *searching trial or study registers*.

Additional information about identifying studies

Handsearching WHO/CDC reports and national screening program guidelines

Link to search strategy

A full search strategy is available in the full protocol as described in the *Availability of full protocol* section

Selection process

Studies will be screened independently by at least two people (or person/machine combination) with a process to resolve differences.

Other relevant information about searching and screening

Manual screening via shared spreadsheets (Excel/Google Sheets) with dual-independent review. Non-English studies translated via certified bilingual team members.

DATA COLLECTION PROCESS

Data extraction from published articles and reports

Data will be extracted independently by at least two people (or person/machine combination) with a process to resolve differences.

Authors will not be contacted for further information.

Study risk of bias or quality assessment

Risk of bias will be assessed using: *QUADAS-2*

Data will be assessed independently by at least two people (or person/machine combination) with a process to resolve differences.

Additional information will **not** be sought from study investigators if required information is unclear or unavailable in the study publications/reports.

Reporting bias assessment

QUADAS-2 Domain 4 (Flow/Timing) will evaluate attrition bias. Funnel plots/Egger's test applied if ≥10 studies pooled.

Certainty assessment

The certainty of evidence will be evaluated using the GRADE approach (Grading of Recommendations, Assessment, Development, and Evaluations). Key domains include: 1. Risk of bias (QUADAS-2 assessments) 2. Inconsistency (I^2 statistic >50% indicating heterogeneity) 3. Indirectness (e.g., population or test applicability) 4. Imprecision (confidence interval width around sensitivity/specificity estimates) 5. Publication bias (funnel plots if ≥10 studies). Evidence will be graded as high, moderate, low, or very low certainty.

OUTCOMES TO BE ANALYSED

Main outcomes

1. Diagnostic accuracy of AI-assisted routine tests: - Sensitivity, specificity, AUC for detecting carriers of hereditary hemolytic anemia (primary). 2. Comparative performance: - Difference in accuracy (AI vs. conventional interpretation). 3. Clinical utility: - Reduction in unnecessary confirmatory testing (e.g., genetic tests). 4. Implementation metrics: - Feasibility, cost, turnaround time (secondary).

Additional outcomes

1. Ethical/legal challenges: - Data privacy concerns, algorithmic bias, or consent issues in AI-driven screening. 2. Population-specific performance: - Variation in AI accuracy by ethnicity/region (e.g., Mediterranean vs. Southeast Asian cohorts). 3. User acceptance: - Healthcare provider/patient trust in AI results (if reported in studies). 4. Model transparency: - Frequency of explainable AI (XAI) techniques used in included studies.

PLANNED DATA SYNTHESIS

Strategy for data synthesis

If sufficient homogeneous data (≥3 studies reporting compatible outcomes) are available: 1. Primary Meta-Analysis: - Bivariate random-effects models will pool sensitivity, specificity, and AUC values across studies, accounting for between-study variance. - Summary receiver operating characteristic (SROC) curves will visualize diagnostic performance. 2. Secondary Analyses: - Subgroup meta-analyses by: - AI model type (e.g., traditional ML vs. deep learning) - Routine test combination (CBC alone vs. CBC + smear) - Geographic region (high vs. low prevalence areas) 3. Heterogeneity Assessment: - Quantified using I^2 statistic (thresholds: <25% low, 25-75% moderate, >75% high). - Explored via meta-regression if substantial heterogeneity exists. 4. Narrative Synthesis: - For non-quantifiable outcomes (e.g., implementation barriers), findings will be thematically organized using SWIFT-Review or similar tools. Software: R (metafor, mada), Stata, or RevMan.

CURRENT REVIEW STAGE

Stage of the review at this submission

Review stage	Started	Completed
Pilot work		
Formal searching/study identification		
Screening search results against inclusion criteria		
Data extraction or receipt of IPD		
Risk of bias/quality assessment		
Data synthesis		

Review status

The review is currently planned or ongoing.

Publication of review results

Results of the review will be published in English and Arabic.

REVIEW AFFILIATION, FUNDING AND PEER REVIEW

Review team members

Dr Naif Ali (review guarantor and contact) ORCID: 0000-0002-2266-1569. University of Science and technology. Yemen.

No conflict of interest declared.

Dr Hana Ali. ORCID: 0009-0005-6144-4769. Aden Gulf International University. Yemen.

No conflict of interest declared.

Named contact

Dr Naif Ali (n.taleb@ust.edu). ORCID: 0000-0002-2266-1569. University of Science and technology. Yemen.

Review affiliation

University of Science and Technology, Aden, Yemen

University of Lahej, Al-Hoata, Lahej, Yemen

Funding source

Review has no specific/external funding but is supported by guarantor/review team (non-commercial) institutions.

Additional information about funding

No external funding; team members contribute time as part of academic roles.

Peer review

The final manuscript will undergo independent peer review by experts in hematology, diagnostic AI, and public health prior to journal submission. Reviewers will assess methodological rigor, data synthesis validity, and clinical relevance per PRISMA-AI guidelines.

ADDITIONAL INFORMATION

Additional information

This review aligns with WHO recommendations on hereditary anemia prevention. If meta-analysis is unfeasible, a SWiM-guided narrative synthesis will highlight AI's role in reducing screening disparities. Updates will be documented if protocol amendments occur post-registration.

Review conflict of interest

Declared individual interests are recorded under team member details.. No additional interests are recorded for this review.

Medical Subject Headings

Premarital Examinations; Anemia, Hemolytic, Congenital; alpha-Thalassemia; beta-Thalassemia; Anemia, Sickle Cell; Artificial Intelligence; Machine Learning; Diagnostic Tests, Routine; Clinical Laboratory Techniques

Revision note

1. Rationale: Our initial search strategy focused on global and Middle Eastern databases. However, to fully capture the evidence on AI applications in resource-limited settings—a primary objective of our review—it is imperative to include regional databases that index literature from high-prevalence regions for hereditary hemolytic anemias (HHAs), specifically Sub-Saharan Africa and Latin America. AJOL is the premier database for African-published research, and LILACS provides comprehensive coverage of health literature from Latin America and the Caribbean. Omitting these sources risks introducing a selection bias and significantly limiting the generalizability of our findings to these key populations.2. Rationale: The original language restriction was set due to practical constraints. However, we recognize that this exclusion would

systematically omit crucial evidence from Francophone Africa (e.g., Senegal, Côte d'Ivoire, Democratic Republic of the Congo) and Lusophone/Latin American countries (e.g., Brazil), where the prevalence of HHAs is notably high and research in digital health is emerging. Failing to include this evidence would constitute a significant geographic and linguistic bias, undermining the equity focus of our review and potentially skewing our results. Our team has secured resources for professional translation to accommodate this change.3. Rationale: The strict focus on "premarital" screening risks excluding innovative screening initiatives conducted in conflict zones, rural areas, or low-resource settings where formal marriage registration systems may be weak or inaccessible. These programs often share the identical goal of reproductive carrier detection and prevention. I

PROSPERO version history

- Version 1.0, published 12 Jun 2025

Disclaimer

The content of this record displays the information provided by the review team. PROSPERO does not peer review registration records or endorse their content.

PROSPERO accepts and posts the information provided in good faith; responsibility for record content rests with the review team. The guarantor for this record has affirmed that the information provided is truthful and that they understand that deliberate provision of inaccurate information may be construed as scientific misconduct.

PROSPERO does not accept any liability for the content provided in this record or for its use. Readers use the information provided in this record at their own risk.

Any enquiries about the record should be referred to the named review contact
