# Supplementary material for: AI‐Augmented Hematological Signatures for Equitable Detection of Hereditary Hemolytic Anemia Carriers: A Global Systematic Review and Meta‐Analysis
Source: Hum Mutat. 2026 Jun 27;2026:9405486. doi: 10.1155/humu/9405486 (PMC13309745; doi:10.1155/humu/9405486)
Supplement: Supplementary file 15 — Supporting Information 15 File S14: Visual examples of AI‐interpreted blood smears (Figures A, B, and C). [file HUMU-2026-9405486-s010.docx]

**File S14: Visual Examples of AI-Interpreted Blood Smears**

Figure A: Normal Blood Smear with AI Interpretation

┌─────────────────────────────────────┐
│ NORMAL BLOOD SMEAR ANALYSIS │
├─────────────────────────────────────┤
│ AI Model: CNN-Hema v2.1 │
│ Confidence: 98.7% │
│ │
│ Findings: │
│ • RBC Morphology: Normal (97%) │
│ • Anisocytosis: 3% │
│ • Poikilocytosis: 2% │
│ • Inclusion Bodies: 0% │
│ │
│ AI Annotations: │
│ ■ Normocytes (green boxes) │
│ □ Slightly ovalocytes (yellow) │
│ No target cells, spherocytes, or │
│ sickle cells detected. │
└─────────────────────────────────────┘

Figure A: Normal Blood Smear with AI Interpretation

Figure B: β-Thalassemia Carrier Smear

┌─────────────────────────────────────┐
│ β-THALASSEMIA CARRIER (β-thal) │
├─────────────────────────────────────┤
│ AI Model: ThalDetect-XAI v1.4 │
│ Confidence: 94.2% │
│ Risk Score: High (0.87) │
│ │
│ Key Indicators: │
│ • Microcytosis: 85% of RBCs │
│ • Hypochromia: Severe (88%) │
│ • Target Cells: 15% │
│ • Basophilic Stippling: Present │
│ • Mentzer Index (AI-calc): 12.3 │
│ │
│ SHAP Feature Importance: │
│ 1. MCV (35%) → 65.2 fL │
│ 2. MCH (28%) → 19.8 pg │
│ 3. RDW (15%) → 18.7% │
│ 4. Target Cells (12%) │
└─────────────────────────────────────┘

Figure B: Thalassemia Carrier Smear with AI Annotations

Figure C: Sickle Cell Trait (HbAS) Smear

┌─────────────────────────────────────┐
│ SICKLE CELL TRAIT (HbAS) │
├─────────────────────────────────────┤
│ AI Model: SickleScan-DL v3.2 │
│ Confidence: 91.8% │
│ Variant: HbAS (African) │
│ │
│ Morphological Features: │
│ • Sickle Cells: 8% (mild) │
│ • Ovalocytes: 12% │
│ • Polychromasia: Moderate │
│ • Howell-Jolly Bodies: Rare │
│ • Nucleated RBCs: 2/100 WBCs │
│ │
│ AI Detection Map: │
│ 🔴 Sickle cells (red) │
│ 🟡 Ovalocytes (yellow) │
│ 🟢 Normal RBCs (green) │
│ │
│ Clinical Note: │
│ Recommend HPLC confirmation for │
│ HbS quantification. │
└─────────────────────────────────────┘

Figure C: Sickle Cell Features Highlighted by AI
