# Supplementary material for: AI‐Augmented Hematological Signatures for Equitable Detection of Hereditary Hemolytic Anemia Carriers: A Global Systematic Review and Meta‐Analysis
Source: Hum Mutat. 2026 Jun 27;2026:9405486. doi: 10.1155/humu/9405486 (PMC13309745; doi:10.1155/humu/9405486)
Supplement: Supplementary file 16 — Supporting Information 16 File S15: Complete Python code for AI model development and validation. [file HUMU-2026-9405486-s009.docx]

**File S15: COMPLETE PYTHON CODE FOR AI MODEL DEVELOPMENT AND VALIDATION**

Title: Python Code for AI Model Development and Validation in HHA Carrier Detection

Author: Systematic Review Team

OSF Repository: https://osf.io/c8fhw/

DOI: 10.17605/OSF.IO/C8FHW

**1. Setup and Imports**

# =============================================================================

# COMPREHENSIVE AI MODEL DEVELOPMENT AND VALIDATION SCRIPT

# For: AI-Augmented Hematological Signatures for HHA Carrier Detection

# =============================================================================

import numpy as np

import pandas as pd

import matplotlib.pyplot as plt

import seaborn as sns

from sklearn.model_selection import train_test_split, cross_val_score, StratifiedKFold

from sklearn.preprocessing import StandardScaler, LabelEncoder

from sklearn.metrics import (roc_auc_score, accuracy_score, confusion_matrix,

classification_report, roc_curve, auc)

from sklearn.ensemble import RandomForestClassifier, GradientBoostingClassifier

from xgboost import XGBClassifier

import shap

import joblib

import warnings

warnings.filterwarnings('ignore')

# Set random seed for reproducibility

np.random.seed(42)

# Visualization settings

plt.style.use('seaborn-v0_8-whitegrid')

sns.set_palette("husl")

**2. Data Loading and Preprocessing**

def load_and_preprocess_data(filepath):

"""

Load and preprocess hematological data for AI model development.

Parameters:

-----------

filepath : str

Path to the dataset CSV file

Returns:

--------

X : numpy array

Feature matrix

y : numpy array

Target labels

feature_names : list

List of feature names

"""

# Load dataset

data = pd.read_csv(filepath)

print(f"Dataset shape: {data.shape}")

print(f"Columns: {list(data.columns)}")

# Separate features and target

# Assuming target column is 'Carrier_Status' (1 = carrier, 0 = non-carrier)

target_col = 'Carrier_Status'

if target_col not in data.columns:

# Alternative target column names

possible_targets = ['carrier', 'HHA_status', 'target', 'label', 'Class']

for col in possible_targets:

if col in data.columns:

target_col = col

break

# Feature selection (common CBC parameters)

cbc_features = [

'RBC', 'HGB', 'HCT', 'MCV', 'MCH', 'MCHC', 'RDW',

'WBC', 'PLT', 'MPV', 'NEUT', 'LYMPH', 'MONO', 'EO', 'BASO'

]

# Select available features

available_features = [f for f in cbc_features if f in data.columns]

if len(available_features) < 5:

# If few CBC features, use all numeric columns except target

numeric_cols = data.select_dtypes(include=[np.number]).columns.tolist()

if target_col in numeric_cols:

numeric_cols.remove(target_col)

available_features = numeric_cols

X = data[available_features].copy()

y = data[target_col].copy()

# Handle missing values

X = X.fillna(X.median())

# Encode labels if needed

if y.dtype == 'object':

le = LabelEncoder()

y = le.fit_transform(y)

print(f"Features used: {available_features}")

print(f"Target distribution:\n{pd.Series(y).value_counts()}")

return X.values, y, available_features

**3. Model Development Functions**

def train_ai_models(X, y, feature_names):

"""

Train multiple AI models for HHA carrier detection.

Parameters:

-----------

X : numpy array

Feature matrix

y : numpy array

Target labels

feature_names : list

List of feature names

Returns:

--------

results : dict

Dictionary containing trained models and their performance

"""

# Split data

X_train, X_test, y_train, y_test = train_test_split(

X, y, test_size=0.3, random_state=42, stratify=y

)

# Standardize features

scaler = StandardScaler()

X_train_scaled = scaler.fit_transform(X_train)

X_test_scaled = scaler.transform(X_test)

# Initialize models

models = {

'Random Forest': RandomForestClassifier(

n_estimators=100,

max_depth=10,

random_state=42,

class_weight='balanced'

),

'XGBoost': XGBClassifier(

n_estimators=100,

max_depth=6,

learning_rate=0.1,

random_state=42,

eval_metric='logloss'

),

'Gradient Boosting': GradientBoostingClassifier(

n_estimators=100,

max_depth=5,

random_state=42

)

}

results = {}

for model_name, model in models.items():

print(f"\n{'='*50}")

print(f"Training {model_name}")

print(f"{'='*50}")

# Train model

model.fit(X_train_scaled, y_train)

# Predictions

y_pred = model.predict(X_test_scaled)

y_pred_proba = model.predict_proba(X_test_scaled)[:, 1]

# Calculate metrics

accuracy = accuracy_score(y_test, y_pred)

auc_score = roc_auc_score(y_test, y_pred_proba)

# Cross-validation

cv = StratifiedKFold(n_splits=5, shuffle=True, random_state=42)

cv_scores = cross_val_score(model, X_train_scaled, y_train,

cv=cv, scoring='roc_auc')

# Confusion matrix

cm = confusion_matrix(y_test, y_pred)

# Store results

results[model_name] = {

'model': model,

'scaler': scaler,

'accuracy': accuracy,

'auc': auc_score,

'cv_mean_auc': cv_scores.mean(),

'cv_std_auc': cv_scores.std(),

'confusion_matrix': cm,

'y_test': y_test,

'y_pred': y_pred,

'y_pred_proba': y_pred_proba,

'feature_importance': get_feature_importance(model, feature_names)

}

# Print results

print(f"Accuracy: {accuracy:.3f}")

print(f"AUC: {auc_score:.3f}")

print(f"Cross-validation AUC: {cv_scores.mean():.3f} (±{cv_scores.std():.3f})")

print(f"Confusion Matrix:\n{cm}")

return results

def get_feature_importance(model, feature_names):

"""

Extract feature importance from trained model.

Parameters:

-----------

model : sklearn model

Trained model

feature_names : list

List of feature names

Returns:

--------

importance_df : pandas DataFrame

DataFrame with feature importance scores

"""

if hasattr(model, 'feature_importances_'):

importances = model.feature_importances_

elif hasattr(model, 'coef_'):

importances = np.abs(model.coef_[0])

else:

importances = np.zeros(len(feature_names))

importance_df = pd.DataFrame({

'Feature': feature_names,

'Importance': importances

}).sort_values('Importance', ascending=False)

return importance_df

**4. Explainable AI (XAI) with SHAP**

def explain_model_with_shap(model, X, feature_names, sample_size=100):

"""

Generate SHAP explanations for model predictions.

Parameters:

-----------

model : sklearn model

Trained model

X : numpy array

Feature matrix

feature_names : list

List of feature names

sample_size : int

Number of samples to use for SHAP computation

Returns:

--------

shap_values : numpy array

SHAP values

"""

print("\n" + "="*50)

print("Generating SHAP Explanations")

print("="*50)

# Sample data for faster computation

if len(X) > sample_size:

X_sample = X[np.random.choice(len(X), sample_size, replace=False)]

else:

X_sample = X

# Create SHAP explainer

explainer = shap.Explainer(model, X_sample, feature_names=feature_names)

shap_values = explainer(X_sample)

# Summary plot

plt.figure(figsize=(10, 6))

shap.summary_plot(shap_values, X_sample, feature_names=feature_names, show=False)

plt.title("SHAP Feature Importance Summary", fontsize=14)

plt.tight_layout()

plt.savefig("output/figures/shap_summary_plot.png", dpi=300, bbox_inches='tight')

plt.close()

# Bar plot

plt.figure(figsize=(10, 6))

shap.summary_plot(shap_values, X_sample, feature_names=feature_names,

plot_type="bar", show=False)

plt.title("Mean Absolute SHAP Values", fontsize=14)

plt.tight_layout()

plt.savefig("output/figures/shap_bar_plot.png", dpi=300, bbox_inches='tight')

plt.close()

print("SHAP plots saved to output/figures/")

return shap_values

**5. Model Evaluation and Visualization**

def evaluate_and_visualize(results):

"""

Evaluate models and create visualizations.

Parameters:

-----------

results : dict

Dictionary containing trained models and their performance

"""

# Create comparison DataFrame

comparison_data = []

for model_name, result in results.items():

comparison_data.append({

'Model': model_name,

'Accuracy': result['accuracy'],

'AUC': result['auc'],

'CV_AUC_Mean': result['cv_mean_auc'],

'CV_AUC_Std': result['cv_std_auc']

})

comparison_df = pd.DataFrame(comparison_data)

# Save comparison table

comparison_df.to_csv("output/tables/model_comparison.csv", index=False)

print("\n" + "="*50)

print("Model Comparison Results")

print("="*50)

print(comparison_df.to_string())

# ROC curves

plt.figure(figsize=(10, 8))

for model_name, result in results.items():

fpr, tpr, _ = roc_curve(result['y_test'], result['y_pred_proba'])

roc_auc = auc(fpr, tpr)

plt.plot(fpr, tpr, lw=2,

label=f'{model_name} (AUC = {roc_auc:.3f})')

plt.plot([0, 1], [0, 1], 'k--', lw=2, label='Random Chance')

plt.xlim([0.0, 1.0])

plt.ylim([0.0, 1.05])

plt.xlabel('False Positive Rate', fontsize=12)

plt.ylabel('True Positive Rate', fontsize=12)

plt.title('Receiver Operating Characteristic (ROC) Curves', fontsize=14)

plt.legend(loc="lower right", fontsize=10)

plt.grid(True, alpha=0.3)

plt.tight_layout()

plt.savefig("output/figures/roc_curves.png", dpi=300, bbox_inches='tight')

plt.close()

# Feature importance comparison

fig, axes = plt.subplots(1, len(results), figsize=(15, 5))

if len(results) == 1:

axes = [axes]

for idx, (model_name, result) in enumerate(results.items()):

importance_df = result['feature_importance'].head(10)

axes[idx].barh(range(len(importance_df)), importance_df['Importance'])

axes[idx].set_yticks(range(len(importance_df)))

axes[idx].set_yticklabels(importance_df['Feature'])

axes[idx].invert_yaxis()

axes[idx].set_xlabel('Importance')

axes[idx].set_title(f'{model_name} - Top 10 Features')

plt.tight_layout()

plt.savefig("output/figures/feature_importance.png", dpi=300, bbox_inches='tight')

plt.close()

print("\nVisualizations saved to output/figures/")

**6. Main Execution Pipeline**

def main():

"""

Main execution pipeline for AI model development.

"""

print("="*60)

print("AI MODEL DEVELOPMENT FOR HHA CARRIER DETECTION")

print("="*60)

# Step 1: Load data

print("\nStep 1: Loading and preprocessing data...")

data_file = "data/raw/hematological_data.csv" # Update with your file path

X, y, feature_names = load_and_preprocess_data(data_file)

# Step 2: Train models

print("\nStep 2: Training AI models...")

results = train_ai_models(X, y, feature_names)

# Step 3: XAI analysis

print("\nStep 3: Performing explainable AI analysis...")

best_model_name = max(results.items(), key=lambda x: x[1]['auc'])[0]

best_model = results[best_model_name]['model']

shap_values = explain_model_with_shap(best_model, X, feature_names)

# Step 4: Evaluate and visualize

print("\nStep 4: Evaluating models and creating visualizations...")

evaluate_and_visualize(results)

# Step 5: Save best model

print("\nStep 5: Saving best model...")

best_result = results[best_model_name]

joblib.dump(best_result['model'], "output/models/best_ai_model.pkl")

joblib.dump(best_result['scaler'], "output/models/scaler.pkl")

print("\n" + "="*60)

print("AI MODEL DEVELOPMENT COMPLETED SUCCESSFULLY!")

print("="*60)

print(f"Best model: {best_model_name}")

print(f"Best AUC: {best_result['auc']:.3f}")

print(f"Model saved to: output/models/best_ai_model.pkl")

return results

# Execute main function

if __name__ == "__main__":

results = main()

**7. Model Validation on External Dataset**

def validate_on_external_data(model_path, scaler_path, external_data_path):

"""

Validate trained model on external dataset.

Parameters:

-----------

model_path : str

Path to saved model

scaler_path : str

Path to saved scaler

external_data_path : str

Path to external validation dataset

"""

print("\n" + "="*50)

print("External Validation")

print("="*50)

# Load model and scaler

model = joblib.load(model_path)

scaler = joblib.load(scaler_path)

# Load external data

external_data = pd.read_csv(external_data_path)

# Prepare features (adjust based on your dataset)

# Assuming same feature names as training

X_ext = external_data.drop('Carrier_Status', axis=1)

y_ext = external_data['Carrier_Status']

# Preprocess

X_ext_scaled = scaler.transform(X_ext)

# Predict

y_ext_pred = model.predict(X_ext_scaled)

y_ext_pred_proba = model.predict_proba(X_ext_scaled)[:, 1]

# Calculate metrics

accuracy = accuracy_score(y_ext, y_ext_pred)

auc_score = roc_auc_score(y_ext, y_ext_pred_proba)

print(f"External Validation Results:")

print(f"Accuracy: {accuracy:.3f}")

print(f"AUC: {auc_score:.3f}")

print(f"Classification Report:\n{classification_report(y_ext, y_ext_pred)}")

# Confusion matrix

cm = confusion_matrix(y_ext, y_ext_pred)

plt.figure(figsize=(6, 5))

sns.heatmap(cm, annot=True, fmt='d', cmap='Blues')

plt.title('Confusion Matrix - External Validation')

plt.ylabel('True Label')

plt.xlabel('Predicted Label')

plt.tight_layout()

plt.savefig("output/figures/external_validation_cm.png", dpi=300)

plt.close()

return {

'accuracy': accuracy,

'auc': auc_score,

'confusion_matrix': cm

}

**8. Utility Functions**

def generate_model_card(model_info, results):

"""

Generate a model card for documentation.

Parameters:

-----------

model_info : dict

Model information

results : dict

Model performance results

"""

model_card = f"""

========================================================

AI MODEL CARD: HHA CARRIER DETECTION

========================================================

Model Information:

------------------

Model Name: {model_info.get('name', 'HHA_Carrier_Detector')}

Algorithm: {model_info.get('algorithm', 'Ensemble')}

Version: {model_info.get('version', '1.0')}

Date: {pd.Timestamp.now().strftime('%Y-%m-%d')}

Training Data:

--------------

Samples: {model_info.get('n_samples', 'N/A')}

Features: {model_info.get('n_features', 'N/A')}

Class Distribution: {model_info.get('class_dist', 'N/A')}

Performance Metrics:

--------------------

"""

for model_name, result in results.items():

model_card += f"""

{model_name}:

- Accuracy: {result['accuracy']:.3f}

- AUC: {result['auc']:.3f}

- Cross-validation AUC: {result['cv_mean_auc']:.3f} (±{result['cv_std_auc']:.3f})

"""

model_card += f"""

Ethical Considerations:

-----------------------

1. The model should be validated across diverse populations

2. Regular bias audits are recommended

3. Clinical validation is required before deployment

Limitations:

------------

1. Performance may vary with different laboratory equipment

2. Requires standardized CBC measurements

3. Not a replacement for confirmatory genetic testing

Contact:

--------

For questions: [Your Contact Information]

========================================================

"""

# Save model card

with open("output/models/model_card.txt", "w") as f:

f.write(model_card)

print("Model card generated: output/models/model_card.txt")

return model_card
