# Supplementary material for: AI‐Augmented Hematological Signatures for Equitable Detection of Hereditary Hemolytic Anemia Carriers: A Global Systematic Review and Meta‐Analysis
Source: Hum Mutat. 2026 Jun 27;2026:9405486. doi: 10.1155/humu/9405486 (PMC13309745; doi:10.1155/humu/9405486)
Supplement: Supplementary file 17 — Supporting Information 17 File S16: GRADE assessment details. [file HUMU-2026-9405486-s008.docx]

Supplementary File S16: Detailed GRADE Assessment

**GRADE (Grading of Recommendations, Assessment, Development, and Evaluations) Approach for Diagnostic Accuracy Studies**

**Application to Systematic Review of AI-Augmented HHA Carrier Detection**

*Reference: Schünemann HJ, et al. GRADE guidelines: 21 part 1—study design, risk of bias, and indirectness in rating the certainty of evidence. J Clin Epidemiol. 2020;122:129-141.*

1. GRADE Methodology for Diagnostic Accuracy Studies

1.1 Domains for Rating Certainty of Evidence

We applied the GRADE framework adapted for diagnostic accuracy studies across five domains:

1. **Risk of Bias:** Using QUADAS-2 tool results (Supplementary File S3)
2. **Inconsistency:** Statistical heterogeneity (I² statistic > 50% considered substantial)
3. **Indirectness:** Population, intervention, comparator, or outcome mismatches
4. **Imprecision:** Width of confidence intervals around sensitivity/specificity estimates
5. **Publication Bias:** Deeks' funnel plot asymmetry test (p < 0.05 significant)

1.2 Rating Scheme

- **High Certainty:** We are very confident that the true effect lies close to that of the estimate.
- **Moderate Certainty:** We are moderately confident in the effect estimate; the true effect is likely to be close to the estimate.
- **Low Certainty:** Our confidence in the effect estimate is limited; the true effect may be substantially different.
- **Very Low Certainty:** We have very little confidence in the effect estimate; the true effect is likely to be substantially different.

1.3 Decision Rules for Downgrading

| Domain | Criteria for Downgrading | Decision Rule |
| --- | --- | --- |
| **Risk of Bias** | High risk in ≥2 QUADAS-2 domains | Downgrade by 1 level |
|  | High risk in ≥3 QUADAS-2 domains | Downgrade by 2 levels |
| **Inconsistency** | I² > 50% with unexplained heterogeneity | Downgrade by 1 level |
|  | I² > 75% with conflicting subgroup results | Downgrade by 2 levels |
| **Indirectness** | Population differs substantially from target | Downgrade by 1 level |
|  | Intervention differs (e.g., research vs. clinical samples) | Downgrade by 1 level |
|  | Comparator differs from standard care | Downgrade by 1 level |
| **Imprecision** | 95% CI for sensitivity includes <85% or >95% | Downgrade by 1 level |
|  | 95% CI for specificity includes <85% or >95% | Downgrade by 1 level |
|  | Optimal Information Size (OIS) not met* | Downgrade by 1 level |
| **Publication Bias** | Deeks' test p < 0.05 | Downgrade by 1 level |
|  | Funnel plot asymmetry evident | Downgrade by 1 level |

*OIS for diagnostic accuracy: ≥100 cases and ≥100 non-cases for precise estimates.*

2. Study-Level GRADE Assessments

2.1 Assessment Template Applied to Each Study

For each of the 85 included studies, we applied the following assessment:

Study_ID, Risk_of_Bias_Score, Inconsistency_Score, Indirectness_Score, Imprecision_Score, Publication_Bias_Score, Overall_Certainty, Justification

2.2 Detailed Study Assessments

**Table 1. GRADE Assessments for All Included Studies**

| Study ID | Risk of Bias | Inconsistency | Indirectness | Imprecision | Publication Bias | Overall Certainty | Key Justifications |
| --- | --- | --- | --- | --- | --- | --- | --- |
| Study1 | Not serious | Not serious | Not serious | Not serious | Undetected | High | Low QUADAS-2 risk, precise estimates, direct population |
| Study2 | Not serious | Not serious | Not serious | Not serious | Undetected | High | Well-conducted validation, adequate sample size |
| Study3 | Serious | Not serious | Not serious | Not serious | Undetected | Moderate | High risk in patient selection (convenience sampling) |
| Study4 | Not serious | Not serious | Not serious | Not serious | Undetected | High | Prospective design, consecutive sampling |
| Study5 | Serious | Not serious | Not serious | Not serious | Undetected | Moderate | Unclear blinding of index test interpreters |
| Study6 | Not serious | Not serious | Not serious | Not serious | Undetected | High | Community-based sampling, good methodology |
| Study7 | Serious | Not serious | Serious | Not serious | Undetected | Low | High risk in patient selection + research-grade samples |
| Study8 | Not serious | Not serious | Not serious | Not serious | Undetected | High | Low risk across all QUADAS-2 domains |
| Study9 | Not serious | Not serious | Not serious | Not serious | Undetected | High | XAI model with full transparency |
| Study10 | Not serious | Not serious | Not serious | Not serious | Undetected | High | Multicenter validation, robust design |
| Study11 | Serious | Not serious | Serious | Serious | Undetected | Very Low | Multiple domains: small sample, indirect population, high bias |
| Study12 | Not serious | Not serious | Not serious | Not serious | Undetected | High | Large sample, appropriate reference standard |
| Study13 | Serious | Serious | Serious | Serious | Undetected | Very Low | Conflict zone setting, multiple methodological issues |
| Study14 | Not serious | Not serious | Not serious | Not serious | Undetected | High | Standardized protocol, adequate power |
| Study15 | Serious | Not serious | Not serious | Not serious | Undetected | Moderate | Retrospective design with potential selection bias |
| Study54 (Yemen) | Serious | Serious | Serious | Serious | Undetected | Very Low | Conflict zone, unstable conditions, small sample relative to prevalence |
| Study81 (Turkey) | Not serious | Not serious | Not serious | Not serious | Undetected | High | Exceptionally large sample (n=5410), precise estimates |

Complete assessments for all 85 studies available in machine-readable format in Section 4.

3. Outcome-Level GRADE Assessments

3.1 Primary Outcome: Diagnostic Accuracy of AI-Augmented Screening

| Outcome | Certainty | Reasons for Rating | Implications |
| --- | --- | --- | --- |
| Pooled Sensitivity (92.8%) | ⭐⭐⭐⭐ High | - 68% of studies low risk of bias in index test<br>- Consistent direction across subgroups<br>- Precise estimate (95% CI: 91.3–94.1%)<br>- Large total sample (n=133,498) | Strong evidence that AI improves sensitivity |
| Pooled Specificity (91.5%) | ⭐⭐⭐ Moderate | - Downgraded for inconsistency (I²=72%)<br>- Variation across AI model types<br>- Otherwise precise and direct evidence | Good evidence but some variability in specificity |
| Pooled AUC (0.93) | ⭐⭐⭐⭐ High | - Consistent across sensitivity analyses<br>- Robust to exclusion of high-bias studies<br>- Clinically meaningful threshold (>0.90) | Strong evidence of overall diagnostic accuracy |

3.2 Secondary Outcomes

| Outcome | Certainty | Reasons for Rating | Implications |
| --- | --- | --- | --- |
| AI vs. Conventional (Δ +12.3%) | ⭐⭐⭐ Moderate | - Direct comparisons in subset of studies<br>- Some risk of bias in conventional arm assessments<br>- Consistent direction of effect | Good evidence of AI superiority |
| Geographic Disparities | ⭐⭐⭐ Moderate | - Clear pattern but limited African studies (n=13)<br>- Confounding by infrastructure differences<br>- Consistent across multiple African countries | Evidence of disparity but mechanisms unclear |
| Cost Savings ($8.50/person) | ⭐⭐ Low | - Based on modeling, not direct measurement<br>- Wide variation by region (95% CI: $5.20–12.30)<br>- Limited real-world implementation studies | Suggestive evidence, needs validation |
| Algorithmic Bias (Δ -8.2% Africa) | ⭐⭐⭐ Moderate | - Consistent finding across African studies<br>- Biological plausibility (different Hb variants)<br>- Limited by small number of African studies | Good evidence of bias needing addressing |

3.3 Subgroup Analyses Certainty

| Subgroup Analysis | Certainty | Key Findings | Evidence Strength |
| --- | --- | --- | --- |
| By AI Model Type | ⭐⭐⭐⭐ High | DL: 95.1% sens, XAI: 94.3% spec | Multiple studies per category, consistent patterns |
| By Test Combination | ⭐⭐⭐⭐ High | CBC+Smear: +5.5% spec (p<0.001) | Direct comparisons within studies |
| By Geographic Region | ⭐⭐⭐ Moderate | Africa: 86.5% vs ME: 94.8% sens | Limited African studies but consistent findings |
| By Reference Standard | ⭐⭐⭐⭐ High | Genetic vs HPLC: minimal differences | Adequate studies with each reference standard |

4. Machine-Readable GRADE Data

4.1 Complete Dataset (CSV Format)

Study_ID,Risk_of_Bias,Inconsistency,Indirectness,Imprecision,Publication_Bias,Overall_Certainty,QUADAS2_Score,*Sample_Size,*Prevalence,Region,AI_Model_Type

Study1,Not serious,Not serious,Not serious,Not serious,Undetected,High,8/10,*2500,*0.051,Middle East,Deep Learning

Study2,Not serious,Not serious,Not serious,Not serious,Undetected,High,9/10,*1820,*0.068,Southeast Asia,Federated Learning

Study3,Serious,Not serious,Not serious,Not serious,Undetected,Moderate,6/10,*3150,*0.082,Middle East,Random Forest

Study4,Not serious,Not serious,Not serious,Not serious,Undetected,High,9/10,*1500,*0.075,Africa,Deep Learning

Study5,Serious,Not serious,Not serious,Not serious,Undetected,Moderate,7/10,*780,*0.028,Europe,Ensemble

Study6,Not serious,Not serious,Not serious,Not serious,Undetected,High,8/10,*1100,*0.087,South Asia,Ensemble

Study7,Serious,Not serious,Serious,Not serious,Undetected,Low,5/10,*680,*0.128,Africa,Random Forest

Study8,Not serious,Not serious,Not serious,Not serious,Undetected,High,9/10,*1250,*0.042,Middle East,Deep Learning

Study9,Not serious,Not serious,Not serious,Not serious,Undetected,High,9/10,*950,*0.073,South Asia,XAI

Study10,Not serious,Not serious,Not serious,Not serious,Undetected,High,8/10,*820,*0.037,Americas,Ensemble

Study11,Serious,Not serious,Serious,Serious,Undetected,Very Low,3/10,*420,*0.197,Middle East,Federated Learning

Study12,Not serious,Not serious,Not serious,Not serious,Undetected,High,8/10,*1300,*0.117,South Asia,Deep Learning

Study13,Serious,Not serious,Not serious,Not serious,Undetected,Moderate,6/10,*380,*0.149,Africa,Random Forest

Study14,Not serious,Not serious,Not serious,Not serious,Undetected,High,9/10,*760,*0.062,Europe,XAI

Study15,Serious,Not serious,Not serious,Not serious,Undetected,Moderate,7/10,*1050,*0.141,South Asia,Ensemble

Study54,Serious,Serious,Serious,Serious,Undetected,Very Low,2/10,*1980,*0.197,Middle East,Edge AI

Study81,Not serious,Not serious,Not serious,Not serious,Undetected,High,10/10,*5410,*0.091,Middle East,Random Forest

Note: Full dataset for 85 studies available in accompanying CSV file.

4.2 QUADAS-2 to GRADE Mapping

**Scoring System:**

- Each QUADAS-2 domain scored 0-2 (0=high risk, 1=unclear, 2=low risk)
- Total score: 0-10
- ≥8/10: Low risk of bias → Not serious
- 5-7/10: Some concerns → Serious
- ≤4/10: High risk → Very serious

5. Summary of Findings (SoF) Table

**Table 2. GRADE Summary of Findings**

| Outcome | Anticipated Absolute Effects* (95% CI) | № of Participants (Studies) | Certainty of Evidence (GRADE) | What Happens |
| --- | --- | --- | --- | --- |
| AI-augmented screening accuracy | Sensitivity: 92.8% (91.3–94.1)<br>Specificity: 91.5% (89.7–93.0)<br>AUC: 0.93 (0.91–0.95) | 133,498 (85 studies) | ⭐⭐⭐⭐ HIGH for sensitivity & AUC<br>⭐⭐⭐ MODERATE for specificity | AI significantly improves carrier detection accuracy |
| Compared to conventional methods | ΔSensitivity: +12.3% (10.8–13.8)<br>ΔSpecificity: +8.7% (7.1–10.3) | 89,450 (62 studies with comparator) | ⭐⭐⭐ MODERATE | AI outperforms manual interpretation |
| Performance in Sub-Saharan Africa | Sensitivity: 86.5% (84.2–88.7)<br>Specificity: 89.2% (86.8–91.3) | 15,230 (13 studies) | ⭐⭐⭐ MODERATE | Lower performance in African populations |
| Cost savings per person screened | $8.50 ($5.20–12.30) | Modeling based on 85 studies | ⭐⭐ LOW | Potential economic benefit but needs validation |
| Reduction in confirmatory testing | 23.7% (19.5–27.8) | Modeling based on 45 studies | ⭐⭐⭐ MODERATE | AI can reduce need for expensive confirmatory tests |

The risk in the intervention group (and its 95% confidence interval) is based on the assumed risk in the comparison group and the relative effect of the intervention (and its 95% CI).

6. Clinical and Research Implications

6.1 High-Certainty Evidence (Recommendations Strongly Supported)

1. AI improves diagnostic accuracy for HHA carrier detection (High certainty)
2. Blood smear integration enhances specificity (High certainty)
3. Deep Learning models achieve highest sensitivity (High certainty)

6.2 Moderate-Certainty Evidence (Recommendations Supported)

1. Implement AI in premarital screening programs (Moderate certainty)
2. Address algorithmic bias against African Hb variants (Moderate certainty)
3. Use XAI models for clinical trust and transparency (Moderate certainty)

6.3 Low-Certainty Evidence (Recommendations Conditional)

1. Cost-effectiveness of AI implementation (Low certainty – needs economic evaluations)

6. Clinical and Research Implications

6.1 High-Certainty Evidence (Recommendations Strongly Supported)

1. AI improves diagnostic accuracy for HHA carrier detection (High certainty)

2. Blood smear integration enhances specificity (High certainty)

3. Deep Learning models achieve highest sensitivity (High certainty)

6.2 Moderate-Certainty Evidence (Recommendations Supported)

1. Implement AI in premarital screening programs (Moderate certainty)

2. Address algorithmic bias against African Hb variants (Moderate certainty)

3. Use XAI models for clinical trust and transparency (Moderate certainty)

6.3 Low-Certainty Evidence (Recommendations Conditional)

1. Cost-effectiveness of AI implementation (Low certainty – needs economic evaluations)

2. Optimal implementation strategies for resource-limited settings (Low certainty – needs implementation research)

6.4 Research Gaps Identified

1. High-quality African studies needed to improve certainty for regional applications

2. Real-world implementation studies to validate cost savings and workflow integration

3. Long-term performance monitoring of AI systems in clinical use

4. Standardized XAI reporting to improve transparency assessment

---

7. Limitations of GRADE Application

7.1 Methodological Challenges

1. Heterogeneity in AI studies: Different AI models, training data, validation methods

2. Evolution of AI technology: Rapid advancements may make some studies quickly outdated

3. Publication bias in AI research: Positive results more likely published

4. Industry sponsorship: 18% of studies had industry funding (potential conflict of interest)

7.2 Adaptation for AI Diagnostics

We adapted GRADE for AI diagnostics by:

· Considering algorithm transparency as part of risk of bias

· Evaluating training data quality under indirectness

· Assessing computational reproducibility under inconsistency

· Including ethical implementation considerations

7.3 Future Directions for GRADE in AI Reviews

1. Develop AI-specific risk of bias tools (complementing QUADAS-2)

2. Establish minimum reporting standards for AI diagnostic studies

3. Create GRADE guidance specifically for AI/ML interventions

4. Incorporate algorithmic fairness assessments into certainty evaluations

---

8. Verification and Transparency

8.1 Assessment Process

· Two independent reviewers conducted GRADE assessments

· Discrepancies resolved through consensus (third reviewer if needed)

· All assessments documented with justifications

· Process audited by senior methodology expert

8.2 Data Availability

· Complete GRADE assessments: Available in CSV format

· Decision rules: Documented in this file (Section 1.3)

· Raw data: Available in OSF repository (10.17605/OSF.IO/C8FHW)

· Analysis code: Available in Supplementary File S5 (R code)

8.3 Updates and Corrections

This GRADE assessment will be updated if:

· New studies meeting inclusion criteria are published

· Methodological advances in GRADE for AI diagnostics occur

· Errors in assessment are identified and verified
