# Supplementary material for: AI‐Augmented Hematological Signatures for Equitable Detection of Hereditary Hemolytic Anemia Carriers: A Global Systematic Review and Meta‐Analysis
Source: Hum Mutat. 2026 Jun 27;2026:9405486. doi: 10.1155/humu/9405486 (PMC13309745; doi:10.1155/humu/9405486)
Supplement: Supplementary file 19 — Supporting Information 19 File S18: Ethics statement for studies in conflict zones. [file HUMU-2026-9405486-s003.docx]

**File S18: Ethics Approvals by Region**

| Region/Country | Ethics Committee | Approval Reference | Consent Procedures | Cultural Adaptations |
| --- | --- | --- | --- | --- |
| Yemen | Yemeni Ministry of Health Ethics Committee | YMOH-ERC-2024-087 | Written + verbal explanation in Arabic | Community health worker mediation, family involvement |
| Somalia | Somali Health Research Institute | SHRI-IRB-2023-154 | Witnessed verbal consent | Clan leader approval, group discussions |
| Sudan | University of Khartoum Ethics Board | UoK-IRB-2024-203 | Written consent (Arabic/English) | Gender-matched interviewers, female-only spaces |
| Nigeria | National Health Research Ethics Committee | NHREC-2024-045 | Written informed consent | Local language pamphlets, radio announcements |
| Mali | Mali Ministry of Health Ethics Committee | MMOH-ERC-2023-112 | Verbal consent with thumbprint | Community elder participation, traditional healers |
| Conflict Zones | General WHO Emergency Ethics Review Panel | WHO-EERP-2024-028 | Simplified consent process | Mobile clinics, community-based approaches |
| European Studies | Local institutional review boards | Various | Standard written consent | Multicultural adaptations, interpreter services |

**Ethical Considerations Addressed:**

Autonomy: Informed consent in appropriate language/format

Beneficence: Clear explanation of benefits/risks

Non-maleficence: Privacy protection, stigma avoidance

Justice: Equitable access, no exclusion based on literacy

Cultural Respect: Local customs, gender considerations, traditional practices

**Conflict Zone Specific Protocols:**

Safety First: Screening suspended during active conflict

Community Protection: No identifying information recorded

Flexible Implementation: Mobile clinics, temporary locations

Continuity Planning: Data backup, alternative sites
