# Supplementary material for: AI‐Augmented Hematological Signatures for Equitable Detection of Hereditary Hemolytic Anemia Carriers: A Global Systematic Review and Meta‐Analysis
Source: Hum Mutat. 2026 Jun 27;2026:9405486. doi: 10.1155/humu/9405486 (PMC13309745; doi:10.1155/humu/9405486)
Supplement: Supplementary file 20 — Supporting Information 20 File S19: Executive summary for health policymakers. [file HUMU-2026-9405486-s029.docx]

**File S19: Executive Summary for Policymakers**

| Priority Area | Key Finding | Evidence Level | Implementation Timeline | Cost-Benefit | Action Required |
| --- | --- | --- | --- | --- | --- |
| Diagnostic Accuracy | AI improves sensitivity by 12.3% vs conventional methods | High certainty (GRADE) | Short-term (0-6 months) | $8.50 savings/person | Adopt AI-augmented CBC+smear |
| Geographic Equity | African performance 8.2% lower due to data gaps | Moderate certainty | Medium-term (6-18 months) | Requires initial investment | Establish African federated learning hubs |
| Infrastructure | 76% of African clinics report power/internet issues | High certainty | Short-medium term | Solar+backup: $200-500/device | Deploy edge AI with offline capability |
| Algorithmic Bias | 30% models underperform for African Hb variants | Moderate certainty | Ongoing | Audit costs: minimal | Mandate bias audits in regulatory approval |
| Cost-Effectiveness | Break-even at 700-1,250 screens depending on region | High certainty | Short-term ROI: 18-24 months | Scale screening programs >2,000/year |  |
| Implementation | Tiered approach needed based on infrastructure | High certainty | Phased | Basic: $120/device, Advanced: $500/device | Match technology to setting capacity |

**Immediate Actions (0-6 months):**

Pilot Programs: 5 countries (Nigeria, Ghana, Saudi Arabia, India, Bangladesh)

Device Procurement: 100 edge AI devices for field testing

Training: 200 healthcare workers in AI-assisted screening

Policy Development: National guidelines for AI in screening

**Medium-term Goals (6-18 months):**

Scale-up: 20 high-burden countries

Integration: Health system integration in 10 countries

Monitoring: Real-time performance tracking system

Capacity Building: Regional training centers

**Long-term Vision (18-36 months):**

Universal Access: AI-assisted screening in all high-burden regions

Continuous Improvement: Federated learning updates every 6 months

Sustainability: Local manufacturing/maintenance capacity

Expansion: Apply to other genetic disorders
