# Supplementary material for: AI‐Augmented Hematological Signatures for Equitable Detection of Hereditary Hemolytic Anemia Carriers: A Global Systematic Review and Meta‐Analysis
Source: Hum Mutat. 2026 Jun 27;2026:9405486. doi: 10.1155/humu/9405486 (PMC13309745; doi:10.1155/humu/9405486)
Supplement: Supplementary file 21 — Supporting Information 21 File S20: Impact statement. [file HUMU-2026-9405486-s023.docx]

# File S20: Impact Statement

# AI-Augmented Hematological Signatures for Equitable Detection of Hereditary Hemolytic Anemia Carriers

## Executive Impact Summary

### Global Health Significance

This systematic review and meta-analysis establishes AI-augmented routine hematological tests (CBC, blood smear) as a transformative, equitable screening solution for hereditary hemolytic anemia (HHA) carriers, addressing a critical gap in global reproductive health. With 330 million carriers worldwide and hemoglobinopathies contributing to 3.4% of under-five mortality in endemic regions, our findings demonstrate that AI can increase detection sensitivity by 12.3% while reducing costs by $8.50 per person screened.

### Three Paradigm Shifts Demonstrated

1. Diagnostic Democratization

- AI transforms $2 routine tests into 92.8% accurate screening tools
- Reduces dependency on $50-100 confirmatory tests inaccessible to 89% of low-income populations
- Enables screening scalability in resource-limited settings previously excluded from genetic services

1. 2. Variant-to-Biomarker Translation

- Validates hematological signatures as functional biomarkers for HBB variants
- Provides low-cost "hematological omics" alternative to expensive multi-omics profiling
- Bridges genotype-phenotype gap through accessible laboratory proxies

1. 3. Equity-by-Design Implementation

- Documents and addresses 8.2% algorithmic bias against African Hb variants
- Proposes federated learning solutions for data sovereignty in underrepresented regions
- Provides tiered implementation framework matching technology to infrastructure capacity

## Evidence-Based Impact Projections

### Clinical Impact (5-Year Horizon)

- Additional carriers detected: 1.23 million per 100 million screened
- Affected births prevented: 307,500 (Hardy-Weinberg equilibrium assumptions)
- DALYs averted: 6.15 million per 100 million screened
- False positive reduction: 6.7 million unnecessary confirmatory tests avoided

### Economic Impact

- Direct savings: $850 million per 100 million screens
- Indirect savings (disease burden): $2.4 billion (prevented complications, disability, mortality)
- Implementation cost: $500 million (devices, training, infrastructure)
- Net benefit: $2.75 billion (5.5:1 return on investment)

### Equity Impact

- Coverage expansion: 40 million additional people reached in Sub-Saharan Africa
- Representation correction: Federated learning hubs targeting 30% African data inclusion (vs. current 12%)
- Infrastructure adaptation: Solar-powered edge AI devices for 76% of African clinics with power instability

## Policy Translation Framework

### Immediate Actions (0-6 Months)

1. Pilot Programs: 5-country implementation (Nigeria, Ghana, Saudi Arabia, India, Bangladesh)

2. Device Standardization: WHO certification of ≤$200 edge AI devices with 48-hour battery

3. Training Protocols: 200 healthcare workers in AI-assisted screening techniques

4. Guideline Development: National policies for AI integration in premarital screening

### Medium-Term Goals (6-18 Months)

1. Scale-up: 20 high-burden countries with >10% prevalence

2. Health System Integration: Electronic health record compatibility in 10 countries

3. Monitoring Systems: Real-time performance tracking with bias detection

4. Capacity Building: Regional training centers in Africa, Middle East, South Asia

### Long-Term Vision (18-36 Months)

1. Universal Access: AI-assisted screening in all regions with >5% HHA prevalence

2. Continuous Improvement: Federated learning updates every 6 months

3. Sustainability: Local manufacturing and maintenance capacity

4. Expansion: Framework application to other genetic disorders (G6PD deficiency, hemoglobin E)

## Research Impact Pathways

### Methodological Contributions

1. First comprehensive synthesis of AI for HHA carrier detection across 23 countries

2. Novel integration of diagnostic accuracy, implementation science, and health equity

3. Standard-setting protocols for data verification and bias assessment in AI diagnostics

4. Reproducibility framework with complete data/code sharing (OSF repository)

### Clinical Practice Transformation

1. Evidence-based test combinations: CBC + blood smear optimization (94.1% specificity)

2. Model selection guidance: Deep Learning for sensitivity (95.1%), XAI for interpretability

3. Implementation tiers: Context-appropriate technology matching

4. Quality assurance: Real-time monitoring and fallback protocols

### Regulatory and Ethical Framework

1. Bias mitigation requirements: Mandatory performance audits across genetic variants

2. Transparency standards: XAI compliance for all screening algorithms

3. Consent adaptation: Tiered approaches for diverse literacy/cultural contexts

4. Data sovereignty: Federated learning preserving local data ownership

## Sustainability and Scalability

### Technical Sustainability

- Device lifespan: 5-year operational capability with regular maintenance
- Model updates: Federated learning ensuring continuous improvement
- Interoperability: Standards-compliant integration with existing health systems
- Backward compatibility: Functionality maintained during infrastructure disruptions

### Financial Sustainability

- Cost recovery: Break-even at 700-1,250 screens depending on region
- Revenue models: Public-private partnerships, insurance reimbursement, cross-subsidization
- Local economies: Job creation in device maintenance, data annotation, program management
- Efficiency gains: 23.7% reduction in confirmatory testing costs

### Health System Sustainability

- Task shifting: General healthcare workers replacing specialist hematologists
- Infrastructure leverage: Utilizing existing laboratory networks and mobile health platforms
- Policy alignment: Integration with national reproductive health and universal health coverage goals
- Monitoring integration: National health information system compatibility

## Risk Mitigation and Contingency Planning

### Technical Risks

- Failure rate: <5% with redundant systems and regular maintenance
- Power instability: Solar + battery backup (48-hour runtime)
- Connectivity loss: Offline-capable edge computing
- Model drift: Quarterly performance monitoring and recalibration

### Implementation Risks

- User adoption: Simplified interfaces, comprehensive training, local adaptation
- Regulatory barriers: Early engagement with national regulatory agencies
- Cultural acceptance: Community leader involvement, culturally appropriate communication
- Sustainability: Phased funding, government commitment, measurable outcomes

### Equity Risks

- Algorithmic bias: Continuous monitoring across demographic subgroups
- Access disparities: Tiered pricing, public sector deployment, rural prioritization
- Digital divide: Low-literacy interfaces, offline functionality, community health worker deployment
- Data exploitation: Clear data governance, local ownership, benefit-sharing agreements

## Knowledge Translation Strategy

### Target Audiences and Messages

1. National Health Ministries:

- Message: "AI screening reduces program costs by 68% while increasing detection by 12.3%"
- Action: Integrate into national premarital screening policies

2. International Agencies (WHO, UNICEF):

- Message: "Equitable AI implementation can prevent 307,500 affected births per 100 million screened"
- Action: Develop global standards and certification pathways

3. Research Community:

- Message: "Federated learning addresses the 12% African data representation crisis"
- Action: Establish multi-center collaboration for diverse data collection

4. Technology Developers:

- Message: "Market of 100+ million annual screens in high-prevalence regions"
- Action: Develop affordable, offline-capable edge AI solutions

### Dissemination Channels

- Scientific: Open-access publication, conference presentations, webinars
- Policy: Policy briefs, ministerial briefings, WHO technical consultations
- Public: Multilingual infographics, community radio programs, social media campaigns
- Training: Online courses, hands-on workshops, train-the-trainer programs

## Conclusion and Call to Action

This review provides not just evidence of AI's diagnostic accuracy, but a comprehensive roadmap for equitable implementation. The 8.2% performance gap for African variants is not a technological limitation but a representation crisis requiring urgent correction through federated learning and targeted investment.

We call for:

1. Immediate investment in African federated learning hubs

2. WHO certification of affordable edge AI devices

3. Mandatory XAI standards for screening algorithms

4. Phased national implementation starting with 5 high-burden countries

The potential is clear: AI can transform HHA screening from an inaccessible luxury to an equitable right, preventing thousands of affected births while respecting local contexts and resource constraints. This represents not just technological innovation, but a fundamental reimagining of how global health equity can be achieved through appropriate, ethical AI deployment.

---

Citation: Ali NT, Abdullah RS, Mehdi MAH, et al. AI-Augmented Hematological Signatures for Equitable Detection of Hereditary Hemolytic Anemia Carriers: A Global Systematic Review and Meta-Analysis. [Journal]. 2025.

Data Availability: https://osf.io/c8fhw/ (DOI: 10.17605/OSF.IO/C8FHW)

Contact: n.taleb@ust.edu
