# Supplementary material for: AI‐Augmented Hematological Signatures for Equitable Detection of Hereditary Hemolytic Anemia Carriers: A Global Systematic Review and Meta‐Analysis
Source: Hum Mutat. 2026 Jun 27;2026:9405486. doi: 10.1155/humu/9405486 (PMC13309745; doi:10.1155/humu/9405486)
Supplement: Supplementary file 23 — Supporting Information 23 File S22: Aggregated meta‐analysis data for key studies (File_S22_Main_Dataset.csv, File_S22_Data_Dictionary.csv, README_S22.txt, File_S22_R_Analysis_Script.R, and File_S22_Python_Analysis_Script.py). [file HUMU-2026-9405486-s030.zip › file s22/S22_1_Data Dictionary.docx]

SUPPLEMENTARY FILE S1: COMPLETE DATA DICTIONARY

**Manuscript:** AI-Augmented Hematological Signatures for Equitable Detection of Hereditary Hemolytic Anemia Carriers: A Global Systematic Review and Meta-Analysis

**Version:** 2.0
**Date:** December 2025
**Total Variables:** 25

Data Dictionary

| Variable Name | Description | Data Type | Allowed Values | Notes/Calculation |
| --- | --- | --- | --- | --- |
| **STUDY CHARACTERISTICS** |  |  |  |  |
| Study_ID | Unique study identifier | Integer | 1-85 | Sequential numbering |
| Authors | Primary authors of the study | String | Text | Format: LastName et al. |
| Year | Publication year | Integer | 2010-2025 | Inclusive range |
| Country | Study location | String | Country names | As reported in study |
| Sample_Size | Number of participants | Integer | ≥100 | Minimum inclusion criteria |
| Prevalence | Carrier prevalence in sample | Decimal | 0.0-1.0 | Proportion of carriers |
| **PERFORMANCE METRICS** |  |  |  |  |
| AI_Model | Type of AI algorithm | String | Deep Learning, Random Forest, XAI, Ensemble, Federated Learning, Edge AI, Mobile CNN | Primary model category |
| Test_Combination | Laboratory tests used | String | CBC only, CBC+Smear, CBC+RDW, CBC+HPLC, Fingerprick, Blood smear, CBC+HbElectro | Test inputs to AI |
| Sensitivity | True positive rate | Decimal | 0.0-1.0 | TP/(TP+FN) |
| Specificity | True negative rate | Decimal | 0.0-1.0 | TN/(TN+FP) |
| AUC | Area under ROC curve | Decimal | 0.0-1.0 | Overall diagnostic accuracy |
| TP | True positives | Integer | ≥0 | Correctly identified carriers |
| FP | False positives | Integer | ≥0 | Non-carriers incorrectly flagged |
| TN | True negatives | Integer | ≥0 | Correctly identified non-carriers |
| FN | False negatives | Integer | ≥0 | Carriers missed by AI |
| **QUALITY ASSESSMENT** |  |  |  |  |
| QUADAS2_Score | Methodological quality score | String | 0/10 to 10/10 | QUADAS-2 tool score |
| GRADE_Certainty | Certainty of evidence | String | High, Moderate, Low, Very Low | GRADE assessment |
| **CONTEXTUAL FACTORS** |  |  |  |  |
| Region | Geographic region | String | Middle East, South Asia, Europe, Americas, Africa | Broad classification |
| Conflict_Zone | Study in conflict area | String | Yes, No | Active conflict during study |
| Low_Resource | Resource-limited setting | String | Yes, No | Based on WHO classification |
| Genomic_Variants | Primary variants studied | String | Beta-thalassemia, HbSS/HbSC, HbE, Mixed | Dominant hemoglobin variants |
| **TECHNICAL DETAILS** |  |  |  |  |
| Device_Cost_USD | Approximate device cost | Integer | 100-500 | US Dollars |
| Training_Time_Hours | Model training time | Numeric | 1-48 | Hours required |
| Inference_Time_Seconds | Time per prediction | Numeric | 0.1-6.0 | Seconds per sample |
| **DERIVED METRICS** |  |  |  |  |
| PPV | Positive Predictive Value | Decimal | Calculated | TP/(TP+FP) |
| NPV | Negative Predictive Value | Decimal | Calculated | TN/(TN+FN) |
| Accuracy | Overall accuracy | Decimal | Calculated | (TP+TN)/(TP+TN+FP+FN) |
