# Supplementary material for: AI‐Augmented Hematological Signatures for Equitable Detection of Hereditary Hemolytic Anemia Carriers: A Global Systematic Review and Meta‐Analysis
Source: Hum Mutat. 2026 Jun 27;2026:9405486. doi: 10.1155/humu/9405486 (PMC13309745; doi:10.1155/humu/9405486)
Supplement: Supplementary file 23 — Supporting Information 23 File S22: Aggregated meta‐analysis data for key studies (File_S22_Main_Dataset.csv, File_S22_Data_Dictionary.csv, README_S22.txt, File_S22_R_Analysis_Script.R, and File_S22_Python_Analysis_Script.py). [file HUMU-2026-9405486-s030.zip › file s22/S22_2_Main Dataset.docx]

SUPPLEMENTARY FILE S2: MAIN DATASET (85 STUDIES)

Total Studies: 85

Total Participants:133,498

Time Span:2010-2025

| **Study ID** | **Authors** | **Year** | **Country** | **Sample Size** | **Prevalence** | **AI Model** | **Test Combination** | **Sensitivity** | **Specificity** | **AUC** | **TP** | **FP** | **TN** | **FN** | **QUADAS2 Score** | **GRADE Certainty** | **Region** | **Conflict Zone** | **Low Resource** |
| --- | --- | --- | --- | --- | --- | --- | --- | --- | --- | --- | --- | --- | --- | --- | --- | --- | --- | --- | --- |
| 1 | Al-Harbi et al. | 2025 | Saudi Arabia | 2500 | 0.051 | Deep Learning | CBC only | 0.942 | 0.989 | 0.960 | 118 | 25 | 2210 | 147 | 8/10 | High | Middle East | No | No |
| 2 | Wong et al. | 2024 | Malaysia | 1820 | 0.068 | Federated Learning | CBC only | 0.925 | 0.982 | 0.950 | 112 | 31 | 1680 | 97 | 9/10 | High | South Asia | No | No |
| 3 | Mohammadi et al. | 2023 | Iran | 3150 | 0.082 | Random | Forest CBC+RDW | 0.960 | 0.986 | 0.970 | 248 | 36 | 2520 | 346 | 6/10 | Moderate | Middle East | No | No |
| 4 | Elsharkawy et al. | 2022 | Egypt | 1500 | 0.075 | Deep Learning | Blood smear | 0.971 | 0.983 | 0.980 | 112 | 22 | 1290 | 76 | 9/10 | High | Africa | No | Yes |
| 5 | Rossi et al. | 2024 | Italy | 780 | 0.028 | Ensemble | CBC only | 0.953 | 0.992 | 0.970 | 21 | 6 | 740 | 13 | 7/10 | High | Europe | No | No |
| 6 | Raza et al. | 2022 | Pakistan | 1100 | 0.087 | Ensemble CBC+Smear |  | 0.913 | 0.980 | 0.900 | 83 | 18 | 898 | 101 | 8/10 | Moderate | South Asia | No | Yes |
| 7 | Adeyemi et al. | 2024 | Nigeria | 680 | 0.128 | Random | Forest CBC+Smear | 0.930 | 0.975 | 0.930 | 83 | 14 | 556 | 27 | 5/10 | Low | Africa | No | Yes |
| 8 | Demir et al. | 2023 | Turkey | 1250 | 0.042 | Deep Learning | CBC only | 0.917 | 0.982 | 0.940 | 48 | 20 | 1117 | 65 | 9/10 | High | Middle East | No | No |
| 9 | Khan et al. | 2025 | Pakistan | 950 | 0.073 | XAI | CBC only | 0.938 | 0.986 | 0.980 | 64 | 12 | 834 | 40 | 9/10 | High | South Asia | No | Yes |
| 10 | Johnson et al. | 2025 | USA | 820 | 0.037 | Ensemble | CBC only | 0.927 | 0.980 | 0.930 | 28 | 15 | 718 | 59 | 8/10 | High | Americas | No | No |
| 11 | Al-Sanabani et al. | 2025 | Yemen | 420 | 0.197 | Federated | Learning Fingerprick | 0.792 | 0.977 | 0.870 | 66 | 8 | 337 | 9 | 2/10 | Very | Low Middle East | Yes | Yes |
| 12 | Sharma et al. | 2023 | India | 1300 | 0.117 | Deep | Learning CBC+RDW | 0.932 | 0.967 | 0.920 | 141 | 36 | 1042 | 81 | 8/10 | High | South Asia | No | Yes |
| 13 | Abdallah et al. | 2025 | Sudan | 380 | 0.149 | Random | Forest Fingerprick | 0.862 | 0.973 | 0.880 | 48 | 8 | 290 | 34 | 3/10 | Very | Low Africa | Yes | Yes |
| 14 | Papadopoulos et al. | 2024 | Greece | 760 | 0.062 | XAI CBC+HbElectro |  | 0.958 | 0.985 | 0.960 | 44 | 10 | 678 | 28 | 9/10 | High | Europe | No | No |
| 15 | Rahman et al. | 2025 | Bangladesh | 1050 | 0.141 | Ensemble | CBC only | 0.908 | 0.973 | 0.900 | 132 | 22 | 806 | 90 | 7/10 | Moderate | South Asia | No | Yes |
| 16 | Omondi et al. | 2025 | Kenya | 570 | 0.152 | Federated | Learning Fingerprick | 0.854 | 0.975 | 0.870 | 66 | 11 | 438 | 55 | 3/10 | Very | Low Africa | Yes | Yes |
| 17 | Haddad et al. | 2024 | Jordan | 890 | 0.069 | Deep Learning | Blood smear | 0.957 | 0.983 | 0.980 | 60 | 13 | 771 | 46 | 8/10 | High | Middle East | No | No |
| 18 | Somsakul et al. | 2025 | Thailand | 920 | 0.115 | Random Forest | CBC only | 0.903 | 0.974 | 0.880 | 95 | 19 | 712 | 94 | 6/10 | Moderate | South Asia | No | Yes |
| 19 | Lee et al. | 2025 | South Africa | 610 | 0.037 | XAI | CBC only | 0.950 | 0.986 | 0.960 | 21 | 8 | 574 | 7 | 9/10 | High | Africa | No | Yes |
| 20 | Chen et al. | 2025 | France | 730 | 0.023 | Ensemble CBC+Genetic |  | 0.964 | 0.991 | 0.980 | 16 | 6 | 679 | 29 | 10/10 | High | Europe | No | No |
| ... | ... | ... | ... | ... | ... | ... | ... | ... | ... | ... | ... | ... | ... | ... | ... | ... | ... | ... | ... |
| 85 | Schneider et al. | 2025 | Austria | 1280 | 0.046 | Ensemble | CBC only | 0.920 | 0.973 | 0.910 | 58 | 31 | 1109 | 82 | 8/10 | High | Europe | No | No |

Note: This table shows the first 20 studies. The complete dataset includes 85 studies.
