# Supplementary material for: AI‐Augmented Hematological Signatures for Equitable Detection of Hereditary Hemolytic Anemia Carriers: A Global Systematic Review and Meta‐Analysis
Source: Hum Mutat. 2026 Jun 27;2026:9405486. doi: 10.1155/humu/9405486 (PMC13309745; doi:10.1155/humu/9405486)
Supplement: Supplementary file 23 — Supporting Information 23 File S22: Aggregated meta‐analysis data for key studies (File_S22_Main_Dataset.csv, File_S22_Data_Dictionary.csv, README_S22.txt, File_S22_R_Analysis_Script.R, and File_S22_Python_Analysis_Script.py). [file HUMU-2026-9405486-s030.zip › file s22/S22_3_README.docx]

SUPPLEMENTARY FILE S3: README & INSTRUCTIONS

# FILE S22: COMPLETE META-ANALYSIS PACKAGE

===============================================================================

## DESCRIPTION:

This package contains complete data, code, and documentation for reproducing all analyses in the manuscript "AI-Augmented Hematological Signatures for Equitable Detection of Hereditary Hemolytic Anemia Carriers: A Global Systematic Review and Meta-Analysis."

## CONTENTS:

- 1. S1_Data_Dictionary.docx - Variable definitions and metadata
- 2. S2_Main_Dataset.docx - Complete dataset (85 studies, 133,498 participants)
- 3. S4_Python_Analysis_Script.docx - Full Python analysis code
- 4. S5_R_Analysis_Script.docx - Full R analysis code
- 5. S6_Excel_Formulas.docx - Excel formulas for analysis

## HOW TO USE:

### A. QUICK START (PYTHON):

1. Install Python 3.8+ and required packages:

pip install pandas numpy matplotlib seaborn scipy statsmodels

2. Copy code from S4_Python_Analysis_Script.docx to a .py file

3. Run: python analysis_script.py

4. Results will be saved in the current directory

### B. QUICK START (R):

1. Install R 4.0+ and required packages:

install.packages(c("meta", "metafor", "ggplot2", "dplyr", "forestplot"))

2. Copy code from S5_R_Analysis_Script.docx to a .R file

3. Run: source("analysis_script.R")

4. Results will be generated automatically

### C. USING EXCEL:

1. Create a new Excel workbook

2. Copy data from S2_Main_Dataset.docx into Sheet1

3. Apply formulas from S6_Excel_Formulas.docx

4. Create pivot tables and charts as needed

## ANALYSES INCLUDED:

- 1. Descriptive statistics of all variables
- 2. Meta-analysis of sensitivity and specificity
- 3. Subgroup analysis by region and AI model
- 4. Meta-regression analysis
- 5. Publication bias assessment
- 6. Sensitivity analyses
- 7. Temporal trend analysis (2010-2025)
- 8. Cost-effectiveness analysis
- 9. Comprehensive visualizations

## OUTPUT FILES GENERATED:

· Statistical_results.csv

· Forest_plot.png

· Funnel_plot.png

· Subgroup_analysis.csv

· Performance_summary.txt

## REPRODUCIBILITY:

· Random seed: 42 (for both Python and R)

· Complete code documentation

· Step-by-step comments

· Error handling included

## CITATION:

When using this dataset, please cite:
Ali NT, Abdullah RS, Mehdi MAH, et al. Aggregated meta-analysis data for AI-augmented HHA carrier detection: A comprehensive dataset from 85 studies (2010-2025). 2025. DOI: 10.17605/OSF.IO/C8FHW

## CONTACT:

For questions: n.taleb@ust.edu

Data manager: r.saleh@ust.edu

VERSION: 2.0

DATE: 15 December 2025
