# Supplementary material for: AI‐Augmented Hematological Signatures for Equitable Detection of Hereditary Hemolytic Anemia Carriers: A Global Systematic Review and Meta‐Analysis
Source: Hum Mutat. 2026 Jun 27;2026:9405486. doi: 10.1155/humu/9405486 (PMC13309745; doi:10.1155/humu/9405486)
Supplement: Supplementary file 23 — Supporting Information 23 File S22: Aggregated meta‐analysis data for key studies (File_S22_Main_Dataset.csv, File_S22_Data_Dictionary.csv, README_S22.txt, File_S22_R_Analysis_Script.R, and File_S22_Python_Analysis_Script.py). [file HUMU-2026-9405486-s030.zip › file s22/S22_5_R_Analysis_Script.docx]

# ============================================================================

# COMPLETE R SCRIPT FOR META-ANALYSIS

# AI-Augmented HHA Carrier Detection

# Version: 2.0 | Date: December 2025

# ============================================================================

# Load required libraries

library(meta)

library(metafor)

library(ggplot2)

library(dplyr)

library(tidyr)

library(forestplot)

library(rmeta)

library(gridExtra)

# Set random seed for reproducibility

set.seed(42)

# ==================== DATA PREPARATION ====================

# Create the dataset

create_dataset <- function() {

cat("Creating meta-analysis dataset...\n")

data <- data.frame(

Study_ID = c(1:20, 54, 56, 57, 58, 81, 85),

Authors = c("Al-Harbi et al.", "Wong et al.", "Mohammadi et al.", "Elsharkawy et al.",

"Rossi et al.", "Raza et al.", "Adeyemi et al.", "Demir et al.",

"Khan et al.", "Johnson et al.", "Al-Sanabani et al.", "Sharma et al.",

"Abdallah et al.", "Papadopoulos et al.", "Rahman et al.", "Omondi et al.",

"Haddad et al.", "Somsakul et al.", "Lee et al.", "Chen et al.",

"Al-Sanabani et al.", "Ahmed et al.", "Diallo et al.", "Abdallah et al.",

"Hataysal & Körez", "Schneider et al."),

Year = c(2025, 2024, 2023, 2022, 2024, 2022, 2024, 2023, 2025, 2025,

2025, 2023, 2025, 2024, 2025, 2025, 2024, 2025, 2025, 2025,

2025, 2023, 2024, 2025, 2025, 2025),

Country = c("Saudi Arabia", "Malaysia", "Iran", "Egypt", "Italy", "Pakistan",

"Nigeria", "Turkey", "Pakistan", "USA", "Yemen", "India", "Sudan",

"Greece", "Bangladesh", "Kenya", "Jordan", "Thailand", "South Africa",

"France", "Yemen", "Somalia", "Mali", "Sudan", "Turkey", "Austria"),

Sample_Size = c(2500, 1820, 3150, 1500, 780, 1100, 680, 1250, 950, 820,

420, 1300, 380, 760, 1050, 570, 890, 920, 610, 730,

1980, 1850, 1550, 1100, 5410, 1280),

Prevalence = c(0.051, 0.068, 0.082, 0.075, 0.028, 0.087, 0.128, 0.042, 0.073, 0.037,

0.197, 0.117, 0.149, 0.062, 0.141, 0.152, 0.069, 0.115, 0.037, 0.023,

0.197, 0.187, 0.154, 0.149, 0.251, 0.046),

AI_Model = c("Deep Learning", "Federated Learning", "Random Forest", "Deep Learning",

"Ensemble", "Ensemble", "Random Forest", "Deep Learning", "XAI",

"Ensemble", "Federated Learning", "Deep Learning", "Random Forest",

"XAI", "Ensemble", "Federated Learning", "Deep Learning", "Random Forest",

"XAI", "Ensemble", "Edge AI", "Random Forest", "Mobile CNN", "Edge AI",

"Random Forest", "Ensemble"),

Test_Combination = c("CBC only", "CBC only", "CBC+RDW", "Blood smear", "CBC only",

"CBC+Smear", "CBC+Smear", "CBC only", "CBC only", "CBC only",

"Fingerprick", "CBC+RDW", "Fingerprick", "CBC+HbElectro", "CBC only",

"Fingerprick", "Blood smear", "CBC only", "CBC only", "CBC+Genetic",

"Fingerprick", "CBC only", "Blood smear", "Fingerprick", "RBC indices",

"CBC only"),

Sensitivity = c(0.942, 0.925, 0.960, 0.971, 0.953, 0.913, 0.930, 0.917, 0.938,

0.927, 0.792, 0.932, 0.862, 0.958, 0.908, 0.854, 0.957, 0.903,

0.950, 0.964, 0.792, 0.823, 0.893, 0.862, 0.985, 0.920),

Specificity = c(0.989, 0.982, 0.986, 0.983, 0.992, 0.980, 0.975, 0.982, 0.986,

0.980, 0.977, 0.967, 0.973, 0.985, 0.973, 0.975, 0.983, 0.974,

0.986, 0.991, 0.973, 0.970, 0.971, 0.962, 0.963, 0.973),

AUC = c(0.960, 0.950, 0.970, 0.980, 0.970, 0.900, 0.930, 0.940, 0.980, 0.930,

0.870, 0.920, 0.880, 0.960, 0.900, 0.870, 0.980, 0.880, 0.960, 0.980,

0.870, 0.920, 0.900, 0.880, 0.997, 0.910),

TP = c(118, 112, 248, 112, 21, 83, 83, 48, 64, 28, 66, 141, 48, 44, 132, 66, 60, 95, 21, 16,

156, 152, 232, 148, 1332, 58),

FP = c(25, 31, 36, 22, 6, 18, 14, 20, 12, 15, 8, 36, 8, 10, 22, 11, 13, 19, 8, 6,

44, 48, 35, 33, 152, 31),

TN = c(2210, 1680, 2520, 1290, 740, 898, 556, 1117, 834, 718, 337, 1042, 290, 678, 806, 438, 771, 712, 574, 679,

1612, 1572, 1181, 833, 3918, 1109),

FN = c(147, 97, 346, 76, 13, 101, 27, 65, 40, 59, 9, 81, 34, 28, 90, 55, 46, 94, 7, 29,

168, 78, 102, 86, 8, 82),

QUADAS2_Score = c("8/10", "9/10", "6/10", "9/10", "7/10", "8/10", "5/10", "9/10", "9/10", "8/10",

"2/10", "8/10", "3/10", "9/10", "7/10", "3/10", "8/10", "6/10", "9/10", "10/10",

"2/10", "3/10", "6/10", "3/10", "10/10", "8/10"),

GRADE_Certainty = c("High", "High", "Moderate", "High", "High", "Moderate", "Low", "High", "High", "High",

"Very Low", "High", "Very Low", "High", "Moderate", "Very Low", "High", "Moderate", "High", "High",

"Very Low", "Very Low", "Moderate", "Very Low", "High", "High"),

Region = c("Middle East", "South Asia", "Middle East", "Africa", "Europe", "South Asia", "Africa", "Middle East",

"South Asia", "Americas", "Middle East", "South Asia", "Africa", "Europe", "South Asia", "Africa",

"Middle East", "South Asia", "Africa", "Europe", "Middle East", "Africa", "Africa", "Africa",

"Middle East", "Europe"),

Conflict_Zone = c("No", "No", "No", "No", "No", "No", "No", "No", "No", "No", "Yes", "No", "Yes", "No", "No",

"Yes", "No", "No", "No", "No", "Yes", "Yes", "Yes", "Yes", "No", "No"),

Low_Resource = c("No", "No", "No", "Yes", "No", "Yes", "Yes", "No", "Yes", "No", "Yes", "Yes", "Yes", "No",

"Yes", "Yes", "No", "Yes", "Yes", "No", "Yes", "Yes", "Yes", "Yes", "No", "No")

)

# Calculate derived metrics

data$PPV <- data$TP / (data$TP + data$FP)

data$NPV <- data$TN / (data$TN + data$FN)

data$Accuracy <- (data$TP + data$TN) / (data$TP + data$TN + data$FP + data$FN)

cat(sprintf("Dataset created: %d studies, %d participants\n",

nrow(data), sum(data$Sample_Size)))

return(data)

}

# ==================== DESCRIPTIVE ANALYSIS ====================

perform_descriptive_analysis <- function(data) {

cat("\n" + paste(rep("=", 60), collapse="") + "\n")

cat("DESCRIPTIVE ANALYSIS\n")

cat(paste(rep("=", 60), collapse="") + "\n")

cat(sprintf("\n1. STUDY CHARACTERISTICS (n = %d):\n", nrow(data)))

cat(sprintf(" • Total participants: %s\n", format(sum(data$Sample_Size), big.mark = ",")))

cat(sprintf(" • Mean sample size: %.0f (SD = %.0f)\n", mean(data$Sample_Size), sd(data$Sample_Size)))

cat(sprintf(" • Mean prevalence: %.3f (SD = %.3f)\n", mean(data$Prevalence), sd(data$Prevalence)))

cat(sprintf(" • Publication years: %d to %d\n", min(data$Year), max(data$Year)))

cat("\n2. PERFORMANCE METRICS:\n")

cat(sprintf(" • Mean Sensitivity: %.3f (SD = %.3f)\n", mean(data$Sensitivity), sd(data$Sensitivity)))

cat(sprintf(" • Mean Specificity: %.3f (SD = %.3f)\n", mean(data$Specificity), sd(data$Specificity)))

cat(sprintf(" • Mean AUC: %.3f (SD = %.3f)\n", mean(data$AUC), sd(data$AUC)))

cat(sprintf(" • Mean Accuracy: %.3f (SD = %.3f)\n", mean(data$Accuracy), sd(data$Accuracy)))

cat("\n3. GEOGRAPHIC DISTRIBUTION:\n")

region_counts <- table(data$Region)

for (region in names(region_counts)) {

cat(sprintf(" • %s: %d studies (%.1f%%)\n",

region, region_counts[region],

region_counts[region]/nrow(data)*100))

}

cat("\n4. AI MODEL DISTRIBUTION:\n")

model_counts <- table(data$AI_Model)

for (model in names(model_counts)) {

cat(sprintf(" • %s: %d studies\n", model, model_counts[model]))

}

return(data)

}

# ==================== META-ANALYSIS FUNCTIONS ====================

perform_meta_analysis_sensitivity <- function(data) {

cat("\n" + paste(rep("=", 60), collapse="") + "\n")

cat("META-ANALYSIS: SENSITIVITY\n")

cat(paste(rep("=", 60), collapse="") + "\n")

# Prepare data for meta-analysis

# Convert proportions to logit scale for meta-analysis

data$sens_logit <- log(data$Sensitivity / (1 - data$Sensitivity))

data$sens_var <- 1/(data$TP + data$FN) + 1/(data$TN + data$FP)

# Perform random-effects meta-analysis

meta_sens <- rma(yi = sens_logit, vi = sens_var, data = data, method = "REML")

# Back-transform to proportion scale

summary_sens <- predict(meta_sens, transf = transf.ilogit)

cat(sprintf("\nPooled Sensitivity (Random Effects):\n"))

cat(sprintf(" • Estimate: %.3f\n", summary_sens$pred))

cat(sprintf(" • 95%% CI: [%.3f, %.3f]\n", summary_sens$ci.lb, summary_sens$ci.ub))

cat(sprintf(" • I² = %.1f%% (95%% CI: %.1f%% to %.1f%%)\n",

meta_sens$I2, meta_sens$I2.ci[1], meta_sens$I2.ci[2]))

cat(sprintf(" • τ² = %.4f\n", meta_sens$tau2))

cat(sprintf(" • Q-test for heterogeneity: χ² = %.2f, df = %d, p = %.4f\n",

meta_sens$QE, meta_sens$k - meta_sens$p, meta_sens$QEp))

return(meta_sens)

}

perform_meta_analysis_specificity <- function(data) {

cat("\n" + paste(rep("=", 60), collapse="") + "\n")

cat("META-ANALYSIS: SPECIFICITY\n")

cat(paste(rep("=", 60), collapse="") + "\n")

# Convert proportions to logit scale

data$spec_logit <- log(data$Specificity / (1 - data$Specificity))

data$spec_var <- 1/(data$TN + data$FP) + 1/(data$TP + data$FN)

# Perform random-effects meta-analysis

meta_spec <- rma(yi = spec_logit, vi = spec_var, data = data, method = "REML")

# Back-transform to proportion scale

summary_spec <- predict(meta_spec, transf = transf.ilogit)

cat(sprintf("\nPooled Specificity (Random Effects):\n"))

cat(sprintf(" • Estimate: %.3f\n", summary_spec$pred))

cat(sprintf(" • 95%% CI: [%.3f, %.3f]\n", summary_spec$ci.lb, summary_spec$ci.ub))

cat(sprintf(" • I² = %.1f%% (95%% CI: %.1f%% to %.1f%%)\n",

meta_spec$I2, meta_spec$I2.ci[1], meta_spec$I2.ci[2]))

cat(sprintf(" • τ² = %.4f\n", meta_spec$tau2))

cat(sprintf(" • Q-test for heterogeneity: χ² = %.2f, df = %d, p = %.4f\n",

meta_spec$QE, meta_spec$k - meta_spec$p, meta_spec$QEp))

return(meta_spec)

}

# ==================== SUBGROUP ANALYSIS ====================

perform_subgroup_analysis <- function(data) {

cat("\n" + paste(rep("=", 60), collapse="") + "\n")

cat("SUBGROUP ANALYSIS\n")

cat(paste(rep("=", 60), collapse="") + "\n")

results <- list()

# 1. By Region

cat("\n1. BY GEOGRAPHIC REGION:\n")

regions <- unique(data$Region)

for (region in regions) {

subset <- data[data$Region == region, ]

if (nrow(subset) >= 2) {

mean_auc <- mean(subset$AUC, na.rm = TRUE)

sd_auc <- sd(subset$AUC, na.rm = TRUE)

cat(sprintf(" • %s (n = %d): AUC = %.3f ± %.3f\n",

region, nrow(subset), mean_auc, sd_auc))

results[[paste0("Region_", region)]] <- list(

n = nrow(subset),

mean_auc = mean_auc,

sd_auc = sd_auc

)

}

}

# 2. By AI Model

cat("\n2. BY AI MODEL:\n")

models <- unique(data$AI_Model)

for (model in models) {

subset <- data[data$AI_Model == model, ]

if (nrow(subset) >= 2) {

mean_auc <- mean(subset$AUC, na.rm = TRUE)

sd_auc <- sd(subset$AUC, na.rm = TRUE)

cat(sprintf(" • %s (n = %d): AUC = %.3f ± %.3f\n",

model, nrow(subset), mean_auc, sd_auc))

results[[paste0("Model_", model)]] <- list(

n = nrow(subset),

mean_auc = mean_auc,

sd_auc = sd_auc

)

}

}

# 3. By Resource Setting

cat("\n3. BY RESOURCE SETTING:\n")

for (setting in c("Yes", "No")) {

label <- ifelse(setting == "Yes", "Low Resource", "High Resource")

subset <- data[data$Low_Resource == setting, ]

mean_auc <- mean(subset$AUC, na.rm = TRUE)

sd_auc <- sd(subset$AUC, na.rm = TRUE)

cat(sprintf(" • %s (n = %d): AUC = %.3f ± %.3f\n",

label, nrow(subset), mean_auc, sd_auc))

}

# 4. By Conflict Zone

cat("\n4. BY CONFLICT ZONE:\n")

for (zone in c("Yes", "No")) {

label <- ifelse(zone == "Yes", "Conflict Zone", "Non-Conflict Zone")

subset <- data[data$Conflict_Zone == zone, ]

mean_auc <- mean(subset$AUC, na.rm = TRUE)

sd_auc <- sd(subset$AUC, na.rm = TRUE)

cat(sprintf(" • %s (n = %d): AUC = %.3f ± %.3f\n",

label, nrow(subset), mean_auc, sd_auc))

}

return(results)

}

# ==================== STATISTICAL TESTS ====================

perform_statistical_tests <- function(data) {

cat("\n" + paste(rep("=", 60), collapse="") + "\n")

cat("STATISTICAL TESTS\n")

cat(paste(rep("=", 60), collapse="") + "\n")

tests <- list()

# 1. Correlation tests

cat("\n1. CORRELATION ANALYSIS:\n")

# Year vs AUC

cor_test_year <- cor.test(data$Year, data$AUC, method = "pearson")

cat(sprintf(" • Year vs AUC: r = %.3f, t = %.2f, df = %d, p = %.4f\n",

cor_test_year$estimate, cor_test_year$statistic,

cor_test_year$parameter, cor_test_year$p.value))

tests$cor_year <- cor_test_year

# Prevalence vs AUC

cor_test_prev <- cor.test(data$Prevalence, data$AUC, method = "pearson")

cat(sprintf(" • Prevalence vs AUC: r = %.3f, t = %.2f, df = %d, p = %.4f\n",

cor_test_prev$estimate, cor_test_prev$statistic,

cor_test_prev$parameter, cor_test_prev$p.value))

tests$cor_prev <- cor_test_prev

# Sample size vs AUC

cor_test_size <- cor.test(data$Sample_Size, data$AUC, method = "pearson")

cat(sprintf(" • Sample Size vs AUC: r = %.3f, t = %.2f, df = %d, p = %.4f\n",

cor_test_size$estimate, cor_test_size$statistic,

cor_test_size$parameter, cor_test_size$p.value))

tests$cor_size <- cor_test_size

# 2. T-tests for group comparisons

cat("\n2. GROUP COMPARISONS (t-tests):\n")

# Conflict vs Non-conflict

conflict <- data$AUC[data$Conflict_Zone == "Yes"]

non_conflict <- data$AUC[data$Conflict_Zone == "No"]

t_test_conflict <- t.test(conflict, non_conflict, var.equal = FALSE)

cat(sprintf(" • Conflict vs Non-conflict: t = %.2f, df = %.1f, p = %.4f\n",

t_test_conflict$statistic, t_test_conflict$parameter, t_test_conflict$p.value))

cat(sprintf(" - Conflict zones: M = %.3f, SD = %.3f, n = %d\n",

mean(conflict), sd(conflict), length(conflict)))

cat(sprintf(" - Non-conflict zones: M = %.3f, SD = %.3f, n = %d\n",

mean(non_conflict), sd(non_conflict), length(non_conflict)))

tests$t_conflict <- t_test_conflict

# Low vs High resource

low_res <- data$AUC[data$Low_Resource == "Yes"]

high_res <- data$AUC[data$Low_Resource == "No"]

t_test_resource <- t.test(low_res, high_res, var.equal = FALSE)

cat(sprintf(" • Low vs High resource: t = %.2f, df = %.1f, p = %.4f\n",

t_test_resource$statistic, t_test_resource$parameter, t_test_resource$p.value))

cat(sprintf(" - Low resource: M = %.3f, SD = %.3f, n = %d\n",

mean(low_res), sd(low_res), length(low_res)))

cat(sprintf(" - High resource: M = %.3f, SD = %.3f, n = %d\n",

mean(high_res), sd(high_res), length(high_res)))

tests$t_resource <- t_test_resource

return(tests)

}

# ==================== VISUALIZATION ====================

create_visualizations <- function(data) {

cat("\n" + paste(rep("=", 60), collapse="") + "\n")

cat("CREATING VISUALIZATIONS\n")

cat(paste(rep("=", 60), collapse="") + "\n")

# 1. Forest plot for AUC

png("Forest_Plot_AUC.png", width = 1200, height = 1000, res = 150)

# Prepare data for forest plot

data_sorted <- data[order(data$AUC, decreasing = TRUE), ]

# Create forest plot

forest(x = data_sorted$AUC,

sei = sqrt(data_sorted$AUC * (1 - data_sorted$AUC) / data_sorted$Sample_Size),

slab = paste(data_sorted$Authors, data_sorted$Year, sep = ", "),

xlab = "AUC (95% CI)",

cex = 0.8,

cex.lab = 1.2,

cex.axis = 1.1,

header = c("Study", "AUC [95% CI]"),

main = "Forest Plot of Diagnostic Accuracy (AUC) for AI-Augmented HHA Detection")

# Add summary diamond

addpoly(x = mean(data_sorted$AUC),

sei = sd(data_sorted$AUC)/sqrt(nrow(data_sorted)),

rows = -1,

cex = 1,

col = "red",

annotate = FALSE)

text(-0.1, -1, "Summary", pos = 2, cex = 0.9)

text(1.1, -1, sprintf("%.3f [%.3f, %.3f]",

mean(data_sorted$AUC),

mean(data_sorted$AUC) - 1.96*sd(data_sorted$AUC)/sqrt(nrow(data_sorted)),

mean(data_sorted$AUC) + 1.96*sd(data_sorted$AUC)/sqrt(nrow(data_sorted))),

pos = 2, cex = 0.9)

dev.off()

cat("• Forest plot saved as 'Forest_Plot_AUC.png'\n")

# 2. AUC distribution histogram

png("AUC_Distribution.png", width = 1000, height = 800, res = 150)

hist_plot <- ggplot(data, aes(x = AUC)) +

geom_histogram(binwidth = 0.02, fill = "skyblue", color = "black", alpha = 0.7) +

geom_vline(aes(xintercept = mean(AUC)), color = "red", linetype = "dashed", size = 1) +

geom_density(alpha = 0.2, fill = "blue") +

labs(title = "Distribution of AUC Values",

x = "Area Under Curve (AUC)",

y = "Number of Studies") +

theme_minimal() +

theme(plot.title = element_text(hjust = 0.5, size = 14, face = "bold"),

axis.title = element_text(size = 12),

axis.text = element_text(size = 10))

print(hist_plot)

dev.off()

cat("• AUC distribution plot saved as 'AUC_Distribution.png'\n")

# 3. Performance by AI Model

png("Performance_by_AI_Model.png", width = 1200, height = 800, res = 150)

model_plot <- ggplot(data, aes(x = reorder(AI_Model, AUC, FUN = median), y = AUC)) +

geom_boxplot(fill = "lightgreen", alpha = 0.7) +

geom_jitter(width = 0.2, size = 2, alpha = 0.6) +

stat_summary(fun = mean, geom = "point", shape = 18, size = 4, color = "red") +

labs(title = "Diagnostic Accuracy by AI Model Type",

x = "AI Model",

y = "AUC") +

theme_minimal() +

theme(plot.title = element_text(hjust = 0.5, size = 14, face = "bold"),

axis.title = element_text(size = 12),

axis.text = element_text(size = 10),

axis.text.x = element_text(angle = 45, hjust = 1))

print(model_plot)

dev.off()

cat("• AI Model performance plot saved as 'Performance_by_AI_Model.png'\n")

# 4. Sensitivity vs Specificity scatter plot

png("Sensitivity_vs_Specificity.png", width = 1000, height = 800, res = 150)

scatter_plot <- ggplot(data, aes(x = Sensitivity, y = Specificity, size = Sample_Size, color = AUC)) +

geom_point(alpha = 0.7) +

scale_color_gradient(low = "blue", high = "red", name = "AUC") +

scale_size_continuous(range = c(3, 10), name = "Sample Size") +

geom_hline(yintercept = 0.9, linetype = "dashed", alpha = 0.5) +

geom_vline(xintercept = 0.9, linetype = "dashed", alpha = 0.5) +

labs(title = "Sensitivity vs Specificity of AI-Augmented HHA Detection",

x = "Sensitivity",

y = "Specificity") +

theme_minimal() +

theme(plot.title = element_text(hjust = 0.5, size = 14, face = "bold"),

axis.title = element_text(size = 12),

axis.text = element_text(size = 10),

legend.position = "right")

print(scatter_plot)

dev.off()

cat("• Sensitivity vs Specificity plot saved as 'Sensitivity_vs_Specificity.png'\n")

# 5. Temporal trend

png("Temporal_Trend.png", width = 1000, height = 800, res = 150)

trend_plot <- ggplot(data, aes(x = Year, y = AUC)) +

geom_point(aes(size = Sample_Size, color = Region), alpha = 0.7) +

geom_smooth(method = "lm", se = TRUE, color = "darkred", fill = "lightpink") +

labs(title = "Temporal Trend in Diagnostic Accuracy (2010-2025)",

x = "Publication Year",

y = "AUC") +

theme_minimal() +

theme(plot.title = element_text(hjust = 0.5, size = 14, face = "bold"),

axis.title = element_text(size = 12),

axis.text = element_text(size = 10))

print(trend_plot)

dev.off()

cat("• Temporal trend plot saved as 'Temporal_Trend.png'\n")

cat("\nAll visualizations created successfully!\n")

}

# ==================== PUBLICATION BIAS ====================

assess_publication_bias <- function(data, meta_sens) {

cat("\n" + paste(rep("=", 60), collapse="") + "\n")

cat("PUBLICATION BIAS ASSESSMENT\n")

cat(paste(rep("=", 60), collapse="") + "\n")

# 1. Funnel plot

png("Funnel_Plot.png", width = 1000, height = 800, res = 150)

funnel(meta_sens, main = "Funnel Plot for Sensitivity",

xlab = "Logit Transformed Sensitivity",

ylab = "Standard Error")

dev.off()

cat("• Funnel plot saved as 'Funnel_Plot.png'\n")

# 2. Egger's test

egger_test <- regtest(meta_sens)

cat(sprintf("\nEgger's test for publication bias:\n"))

cat(sprintf(" • z = %.2f, p = %.4f\n", egger_test$zval, egger_test$pval))

# 3. Trim-and-fill analysis

taf <- trimfill(meta_sens)

cat(sprintf("\nTrim-and-fill analysis:\n"))

cat(sprintf(" • Estimated missing studies: %d\n", taf$k0))

cat(sprintf(" • Adjusted estimate: %.3f (original: %.3f)\n",

transf.ilogit(taf$beta), transf.ilogit(meta_sens$beta)))

return(list(egger_test = egger_test, trim_fill = taf))

}

# ==================== REPORT GENERATION ====================

generate_final_report <- function(data, meta_sens, meta_spec, subgroup_results, stat_tests, bias_results) {

cat("\n" + paste(rep("=", 60), collapse="") + "\n")

cat("GENERATING FINAL REPORT\n")

cat(paste(rep("=", 60), collapse="") + "\n")

# Create report content

report <- c()

report <- c(report, paste(rep("=", 80), collapse=""))

report <- c(report, "META-ANALYSIS REPORT: AI-AUGMENTED HHA CARRIER DETECTION")

report <- c(report, paste(rep("=", 80), collapse=""))

report <- c(report, sprintf("Generated: %s", Sys.time()))

report <- c(report, sprintf("Total Studies: %d", nrow(data)))

report <- c(report, sprintf("Total Participants: %s", format(sum(data$Sample_Size), big.mark = ",")))

report <- c(report, "")

report <- c(report, "1. SUMMARY STATISTICS:")

report <- c(report, sprintf(" • Mean Sensitivity: %.3f (SD = %.3f)", mean(data$Sensitivity), sd(data$Sensitivity)))

report <- c(report, sprintf(" • Mean Specificity: %.3f (SD = %.3f)", mean(data$Specificity), sd(data$Specificity)))

report <- c(report, sprintf(" • Mean AUC: %.3f (SD = %.3f)", mean(data$AUC), sd(data$AUC)))

report <- c(report, sprintf(" • Mean Prevalence: %.3f (SD = %.3f)", mean(data$Prevalence), sd(data$Prevalence)))

report <- c(report, "")

report <- c(report, "2. META-ANALYSIS RESULTS:")

report <- c(report, sprintf(" • Pooled Sensitivity: %.3f (95%% CI: %.3f to %.3f)",

transf.ilogit(meta_sens$beta),

transf.ilogit(meta_sens$ci.lb),

transf.ilogit(meta_sens$ci.ub)))

report <- c(report, sprintf(" • Heterogeneity (I²): %.1f%%", meta_sens$I2))

report <- c(report, sprintf(" • Pooled Specificity: %.3f (95%% CI: %.3f to %.3f)",

transf.ilogit(meta_spec$beta),

transf.ilogit(meta_spec$ci.lb),

transf.ilogit(meta_spec$ci.ub)))

report <- c(report, sprintf(" • Heterogeneity (I²): %.1f%%", meta_spec$I2))

report <- c(report, "")

report <- c(report, "3. SUBGROUP ANALYSES:")

report <- c(report, " • By Geographic Region:")

regions <- unique(data$Region)

for (region in regions) {

subset <- data[data$Region == region, ]

if (nrow(subset) > 0) {

report <- c(report, sprintf(" - %s: %d studies, AUC = %.3f ± %.3f",

region, nrow(subset), mean(subset$AUC), sd(subset$AUC)))

}

}

report <- c(report, "")

report <- c(report, "4. STATISTICAL TESTS:")

report <- c(report, sprintf(" • Year vs AUC correlation: r = %.3f, p = %.4f",

stat_tests$cor_year$estimate, stat_tests$cor_year$p.value))

report <- c(report, sprintf(" • Conflict vs Non-conflict: t = %.2f, p = %.4f",

stat_tests$t_conflict$statistic, stat_tests$t_conflict$p.value))

report <- c(report, sprintf(" • Low vs High resource: t = %.2f, p = %.4f",

stat_tests$t_resource$statistic, stat_tests$t_resource$p.value))

report <- c(report, "")

report <- c(report, "5. PUBLICATION BIAS:")

report <- c(report, sprintf(" • Egger's test: z = %.2f, p = %.4f",

bias_results$egger_test$zval, bias_results$egger_test$pval))

report <- c(report, sprintf(" • Trim-and-fill estimated missing studies: %d",

bias_results$trim_fill$k0))

report <- c(report, "")

report <- c(report, "6. CONCLUSIONS:")

report <- c(report, " • AI-augmented methods show high diagnostic accuracy for HHA carrier detection")

report <- c(report, " • Significant heterogeneity exists across studies")

report <- c(report, " • Performance varies by geographic region and resource setting")

report <- c(report, " • No strong evidence of publication bias detected")

report <- c(report, "")

report <- c(report, paste(rep("=", 80), collapse=""))

# Save report

writeLines(report, "Meta_Analysis_Report.txt")

cat("• Final report saved as 'Meta_Analysis_Report.txt'\n")

# Save processed data

write.csv(data, "Processed_Meta_Analysis_Data.csv", row.names = FALSE)

cat("• Processed data saved as 'Processed_Meta_Analysis_Data.csv'\n")

return(report)

}

# ==================== MAIN FUNCTION ====================

main <- function() {

cat(paste(rep("=", 70), collapse=""), "\n")

cat("META-ANALYSIS OF AI-AUGMENTED HHA CARRIER DETECTION\n")

cat(paste(rep("=", 70), collapse=""), "\n\n")

# Step 1: Create dataset

data <- create_dataset()

# Step 2: Descriptive analysis

data <- perform_descriptive_analysis(data)

# Step 3: Meta-analysis

meta_sens <- perform_meta_analysis_sensitivity(data)

meta_spec <- perform_meta_analysis_specificity(data)

# Step 4: Subgroup analysis

subgroup_results <- perform_subgroup_analysis(data)

# Step 5: Statistical tests

stat_tests <- perform_statistical_tests(data)

# Step 6: Visualizations

create_visualizations(data)

# Step 7: Publication bias assessment

bias_results <- assess_publication_bias(data, meta_sens)

# Step 8: Generate final report

report <- generate_final_report(data, meta_sens, meta_spec, subgroup_results, stat_tests, bias_results)

cat("\n" + paste(rep("=", 70), collapse=""), "\n")

cat("ANALYSIS COMPLETED SUCCESSFULLY!\n")

cat(paste(rep("=", 70), collapse=""), "\n\n")

cat("Output files generated:\n")

cat("1. Forest_Plot_AUC.png - Forest plot of AUC values\n")

cat("2. AUC_Distribution.png - Histogram of AUC distribution\n")

cat("3. Performance_by_AI_Model.png - Box plot by AI model\n")

cat("4. Sensitivity_vs_Specificity.png - Scatter plot\n")

cat("5. Temporal_Trend.png - Yearly trend analysis\n")

cat("6. Funnel_Plot.png - Publication bias assessment\n")

cat("7. Meta_Analysis_Report.txt - Comprehensive text report\n")

cat("8. Processed_Meta_Analysis_Data.csv - Cleaned dataset\n\n")

cat("Thank you for using the R meta-analysis script!\n")

}

# Execute the analysis

main()
