# Supplementary material for: AI‐Augmented Hematological Signatures for Equitable Detection of Hereditary Hemolytic Anemia Carriers: A Global Systematic Review and Meta‐Analysis
Source: Hum Mutat. 2026 Jun 27;2026:9405486. doi: 10.1155/humu/9405486 (PMC13309745; doi:10.1155/humu/9405486)
Supplement: Supplementary file 23 — Supporting Information 23 File S22: Aggregated meta‐analysis data for key studies (File_S22_Main_Dataset.csv, File_S22_Data_Dictionary.csv, README_S22.txt, File_S22_R_Analysis_Script.R, and File_S22_Python_Analysis_Script.py). [file HUMU-2026-9405486-s030.zip › file s22/S22_6_Excel Formulas.docx]

SUPPLEMENTARY FILE S6: EXCEL ANALYSIS FORMULAS

**COMPLETE EXCEL FORMULAS FOR META-ANALYSIS**

## Assumptions:

- Data starts at Row 2
- Headers are in Row 1
- Columns A-Z correspond to dataset variables

---

# 1. BASIC DESCRIPTIVE STATISTICS

=COUNT(A:A) # Number of studies
=SUM(E:E) # Total participants
=AVERAGE(E:E) # Mean sample size
=STDEV(E:E) # SD of sample size
=MEDIAN(E:E) # Median sample size

=AVERAGE(F:F) # Mean prevalence
=STDEV(F:F) # SD of prevalence
=MIN(F:F) # Minimum prevalence
=MAX(F:F) # Maximum prevalence

=AVERAGE(I:I) # Mean sensitivity
=STDEV(I:I) # SD of sensitivity
=AVERAGE(J:J) # Mean specificity
=STDEV(J:J) # SD of specificity
=AVERAGE(K:K) # Mean AUC
=STDEV(K:K) # SD of AUC

---

# 2. DERIVED METRICS CALCULATION

# In new columns (assuming data starts at row 2):

# Positive Predictive Value (PPV)
=M2/(M2+N2)

# Negative Predictive Value (NPV)
=O2/(O2+P2)

# Overall Accuracy
=(M2+O2)/(M2+N2+O2+P2)

# Likelihood Ratio Positive
=I2/(1-J2)

# Likelihood Ratio Negative
=(1-I2)/J2

# Diagnostic Odds Ratio
=(M2*O2)/(N2*P2)

# F1 Score
=2*(M2/(M2+N2))*(M2/(M2+P2))/((M2/(M2+N2))+(M2/(M2+P2)))

---

# 3. CONFIDENCE INTERVALS

# 95% CI for Sensitivity (Cell I2)
Lower CI: =I2-1.96*SQRT(I2*(1-I2)/E2)
Upper CI: =I2+1.96*SQRT(I2*(1-I2)/E2)

# 95% CI for Specificity (Cell J2)
Lower CI: =J2-1.96*SQRT(J2*(1-J2)/E2)
Upper CI: =J2+1.96*SQRT(J2*(1-J2)/E2)

# 95% CI for AUC (Cell K2)
Lower CI: =K2-1.96*SQRT(K2*(1-K2)/E2)
Upper CI: =K2+1.96*SQRT(K2*(1-K2)/E2)

---

# 4. SUBGROUP ANALYSIS FORMULAS

# Count studies by Region
=COUNTIF(Q:Q, "Middle East")
=COUNTIF(Q:Q, "South Asia")
=COUNTIF(Q:Q, "Europe")
=COUNTIF(Q:Q, "Americas")
=COUNTIF(Q:Q, "Africa")

# Mean AUC by Region
=AVERAGEIF(Q:Q, "Middle East", K:K)
=AVERAGEIF(Q:Q, "South Asia", K:K)
=AVERAGEIF(Q:Q, "Europe", K:K)
=AVERAGEIF(Q:Q, "Americas", K:K)
=AVERAGEIF(Q:Q, "Africa", K:K)

# Count studies by AI Model
=COUNTIF(G:G, "Deep Learning")
=COUNTIF(G:G, "Random Forest")
=COUNTIF(G:G, "Ensemble")
=COUNTIF(G:G, "XAI")
=COUNTIF(G:G, "Federated Learning")

# Mean AUC by AI Model
=AVERAGEIF(G:G, "Deep Learning", K:K)
=AVERAGEIF(G:G, "Random Forest", K:K)
=AVERAGEIF(G:G, "Ensemble", K:K)
=AVERAGEIF(G:G, "XAI", K:K)
=AVERAGEIF(G:G, "Federated Learning", K:K)

# Performance in Conflict Zones
=COUNTIF(S:S, "Yes") # Number of conflict zone studies
=AVERAGEIF(S:S, "Yes", K:K) # Mean AUC in conflict zones
=AVERAGEIF(S:S, "No", K:K) # Mean AUC in non-conflict zones

# Performance by Resource Setting
=COUNTIF(T:T, "Yes") # Number of low-resource studies
=AVERAGEIF(T:T, "Yes", K:K) # Mean AUC in low-resource
=AVERAGEIF(T:T, "No", K:K) # Mean AUC in high-resource

---

# 5. CORRELATION ANALYSIS

# Correlation between variables
=CORREL(F:F, K:K) # Prevalence vs AUC
=CORREL(E:E, K:K) # Sample Size vs AUC
=CORREL(C:C, K:K) # Year vs AUC
=CORREL(I:I, J:J) # Sensitivity vs Specificity

# Linear regression slope
=SLOPE(K:K, F:F) # Slope: AUC ~ Prevalence
=SLOPE(K:K, C:C) # Slope: AUC ~ Year
=SLOPE(K:K, E:E) # Slope: AUC ~ Sample Size

# R-squared values
=RSQ(K:K, F:F) # R²: AUC ~ Prevalence
=RSQ(K:K, C:C) # R²: AUC ~ Year
=RSQ(K:K, E:E) # R²: AUC ~ Sample Size

---

# 6. QUALITY ASSESSMENT METRICS

# QUADAS-2 Score analysis
=COUNTIF(P:P, "10/10") # Perfect scores
=COUNTIF(P:P, ">=8/10") # High quality (≥8)
=COUNTIF(P:P, "6/10") # Moderate quality
=COUNTIF(P:P, "<=5/10") # Low quality (≤5)

# Mean AUC by quality category
=AVERAGEIF(P:P, "10/10", K:K)
=AVERAGEIF(P:P, ">=8/10", K:K)
=AVERAGEIF(P:P, "6/10", K:K)
=AVERAGEIF(P:P, "<=5/10", K:K)

# GRADE certainty analysis
=COUNTIF(R:R, "High")
=COUNTIF(R:R, "Moderate")
=COUNTIF(R:R, "Low")
=COUNTIF(R:R, "Very Low")

# Mean AUC by GRADE category
=AVERAGEIF(R:R, "High", K:K)
=AVERAGEIF(R:R, "Moderate", K:K)
=AVERAGEIF(R:R, "Low", K:K)
=AVERAGEIF(R:R, "Very Low", K:K)

---

# 7. TIME TREND ANALYSIS

# Yearly averages
# Create a pivot table:
# Rows: Year (Column C)
# Values: AUC (Column K) → Average
# Values: Count of Study_ID

# Yearly performance improvement
=SLOPE(K:K, C:C) # Annual change in AUC

# Recent vs older studies (split at median year)
=MEDIAN(C:C) # Median publication year
=COUNTIF(C:C, ">"&MEDIAN(C:C)) # Recent studies
=AVERAGEIF(C:C, ">"&MEDIAN(C:C), K:K) # Mean AUC recent
=AVERAGEIF(C:C, "<="&MEDIAN(C:C), K:K) # Mean AUC older

---

# 8. PERFORMANCE THRESHOLD ANALYSIS

# Studies meeting quality thresholds
=COUNTIF(K:K, ">=0.90") # Excellent AUC (≥0.90)
=COUNTIF(K:K, ">=0.80") # Good AUC (≥0.80)
=COUNTIF(K:K, ">=0.70") # Acceptable AUC (≥0.70)

# High sensitivity studies
=COUNTIF(I:I, ">=0.90") # Sensitivity ≥90%
=COUNTIF(I:I, ">=0.95") # Sensitivity ≥95%

# High specificity studies
=COUNTIF(J:J, ">=0.90") # Specificity ≥90%
=COUNTIF(J:J, ">=0.95") # Specificity ≥95%

# Combined high performance
=COUNTIFS(I:I, ">=0.90", J:J, ">=0.90") # Both ≥90%
=COUNTIFS(I:I, ">=0.95", J:J, ">=0.95") # Both ≥95%

---

# 9. COST-EFFECTIVENESS ANALYSIS

# Assuming Device_Cost_USD is in Column V
=AVERAGE(V:V) # Mean device cost
=STDEV(V:V) # SD of device cost

# Cost per correct diagnosis
=V2/(I2*J2*E2) # Cost per accurate test

# Performance to cost ratio
=(I2+J2)/2/V2 # Mean accuracy per $100

# Group by cost categories
=COUNTIF(V:V, "<200") # Low cost devices
=AVERAGEIF(V:V, "<200", K:K) # Mean AUC for low cost
=COUNTIF(V:V, ">=200") # Higher cost devices
=AVERAGEIF(V:V, ">=200", K:K) # Mean AUC for higher cost

---

# 10. COMPREHENSIVE SUMMARY TABLE

# Create a summary table in a new sheet:

A1: "Metric" B1: "Value" C1: "Formula Used"

A2: "Total Studies" B2: =COUNTA(A:A)-1 C2: "=COUNTA(A:A)-1"
A3: "Total Participants" B3: =SUM(E:E) C3: "=SUM(E:E)"
A4: "Mean AUC" B4: =AVERAGE(K:K) C4: "=AVERAGE(K:K)"
A5: "Mean Sensitivity" B5: =AVERAGE(I:I) C5: "=AVERAGE(I:I)"
A6: "Mean Specificity" B6: =AVERAGE(J:J) C6: "=AVERAGE(J:J)"
A7: "Mean Prevalence" B7: =AVERAGE(F:F) C7: "=AVERAGE(F:F)"
A8: "Best AUC" B8: =MAX(K:K) C8: "=MAX(K:K)"
A9: "Worst AUC" B9: =MIN(K:K) C9: "=MIN(K:K)"
A10: "AUC Range" B10: =MAX(K:K)-MIN(K:K) C10: "=MAX(K:K)-MIN(K:K)"

---

# 11. PIVOT TABLE RECOMMENDATIONS

RECOMMENDED PIVOT TABLES:

1. Region × AI Model Performance:
 - Rows: Region
 - Columns: AI_Model
 - Values: AUC (Average)
 - Filters: GRADE_Certainty, Conflict_Zone

2. Yearly Trends:
 - Rows: Year
 - Values: AUC (Average), Sample_Size (Sum)
 - Show as: Line chart

3. Quality Assessment:
 - Rows: GRADE_Certainty
 - Columns: QUADAS2_Score categories
 - Values: Count of Study_ID, AUC (Average)

4. Test Combination Analysis:
 - Rows: Test_Combination
 - Values: AUC (Average), Sensitivity (Average), Specificity (Average)
 - Filters: AI_Model

5. Resource Setting Comparison:
 - Rows: Low_Resource
 - Columns: Conflict_Zone
 - Values: AUC (Average), Device_Cost_USD (Average)

---

# 12. CHART RECOMMENDATIONS

RECOMMENDED CHARTS:

1. Scatter Plot:
 - X: Sensitivity
 - Y: Specificity
 - Size: Sample_Size
 - Color: AUC

2. Box and Whisker:
 - Categories: AI_Model
 - Values: AUC
 - Show: Mean, Median, Outliers

3. Histogram:
 - Data: AUC
 - Bins: 10
 - Show: Normal distribution curve

4. Line Chart:
 - X: Year
 - Y: Average AUC (calculated)
 - Add: Trendline, R² value

5. Stacked Bar:
 - Categories: Region
 - Stacks: AI_Model distribution
 - Height: Study count

6. Heat Map:
 - Rows: Region
 - Columns: AI_Model
 - Color intensity: Average AUC

---

# 13. ADVANCED ANALYSIS (ARRAY FORMULAS)

# Weighted average by sample size (AUC)
=SUMPRODUCT(K:K, E:E)/SUM(E:E)

# Pooled sensitivity (inverse variance weighted)
=SUMPRODUCT(I:I, 1/(I:I*(1-I:I)/E2))/SUMPRODUCT(1/(I:I*(1-I:I)/E2))

# Heterogeneity calculation (Cochran's Q)
=SUM(E:E*(K:K-SUMPRODUCT(K:K, E:E)/SUM(E:E))^2)

# I² statistic for heterogeneity
=MAX(0, (Q-(COUNT(A:A)-1))/Q)*100
# Where Q is Cochran's Q from above

# Prediction interval for AUC
=SUMPRODUCT(K:K, E:E)/SUM(E:E) ± 1.96*SQRT(τ² + SE²)
# Where τ² is between-study variance

---

# 14. DATA VALIDATION FORMULAS

# Check for data integrity
=COUNTIF(I:I, "<0") + COUNTIF(I:I, ">1") # Invalid sensitivity values
=COUNTIF(J:J, "<0") + COUNTIF(J:J, ">1") # Invalid specificity values
=COUNTIF(K:K, "<0.5") + COUNTIF(K:K, ">1") # Invalid AUC values
=COUNTIF(F:F, "<0") + COUNTIF(F:F, ">1") # Invalid prevalence

# Check contingency table consistency
=SUM((M:N+O:P<>E:E)*1) # TP+FP+TN+FN ≠ Sample Size
=SUM((M:M/P:P<>I:I/(1-I:I))*(J:J/(1-J:J))*1) # Sensitivity/Specificity mismatch

# Missing data count
=COUNTBLANK(I:I) # Missing sensitivity
=COUNTBLANK(J:J) # Missing specificity
=COUNTBLANK(K:K) # Missing AUC

---

# 15. EXPORT AND REPORTING

# Create summary for copy-paste to Word

="Total studies: "&TEXT(COUNTA(A:A)-1,"#,##0")
="Total participants: "&TEXT(SUM(E:E),"#,##0")
="Mean AUC: "&TEXT(AVERAGE(K:K),"0.000")&" (±"&TEXT(STDEV(K:K),"0.000")&")"
="Mean Sensitivity: "&TEXT(AVERAGE(I:I),"0.000")&" (±"&TEXT(STDEV(I:I),"0.000")&")"
="Mean Specificity: "&TEXT(AVERAGE(J:J),"0.000")&" (±"&TEXT(STDEV(J:J),"0.000")&")"

# Correlation results
="Year vs AUC correlation: r = "&TEXT(CORREL(C:C, K:K),"0.000")
="Prevalence vs AUC correlation: r = "&TEXT(CORREL(F:F, K:K),"0.000")

# Top performing studies
="Best AUC: "&TEXT(MAX(K:K),"0.000")&" by "&INDEX(B:B, MATCH(MAX(K:K), K:K, 0))
="Best Sensitivity: "&TEXT(MAX(I:I),"0.000")&" by "&INDEX(B:B, MATCH(MAX(I:I), I:I, 0))

---

# NOTES FOR EXCEL USERS:

**1. Data Preparation:**

- Copy data from S2_Main_Dataset.docx into Sheet1
- Ensure headers are in Row 1
- Remove any blank rows

**2. Recommended Setup:**

- Sheet1: Raw Data
- Sheet2: Calculated Metrics (use formulas above)
- Sheet3: Summary Tables
- Sheet4: Pivot Tables
- Sheet5: Charts

**3. Tips:**

- Use Table format (Ctrl+T) for automatic range expansion
- Name ranges for easier formula reference
- Use Conditional Formatting for data visualization
- Create Dashboard sheet with key metrics

**4. Troubleshooting:**

- #DIV/0! errors: Check for zero denominators
- #N/A errors: Check data types and ranges
- #VALUE! errors: Check for text in numeric columns
- #REF! errors: Check for deleted cells/ranges

---

# SAVING AND SHARING:

**1. Final File Preparation:**

- Save as: Meta_Analysis_Excel_Tool.xlsx
- Enable macros if using VBA
- Protect formulas if sharing
- Create PDF snapshot for submission

**2. Submission Ready:**

- Ensure all formulas calculate correctly
- Check all charts are visible
- Verify data integrity
- Include instructions sheet

---

**VERSION: 2.0
LAST UPDATED: December 2025
COMPATIBILITY: Excel 2010 and later**
