# Supplementary material for: AI‐Augmented Hematological Signatures for Equitable Detection of Hereditary Hemolytic Anemia Carriers: A Global Systematic Review and Meta‐Analysis
Source: Hum Mutat. 2026 Jun 27;2026:9405486. doi: 10.1155/humu/9405486 (PMC13309745; doi:10.1155/humu/9405486)
Supplement: Supplementary file 24 — Supporting Information 24 File S23: Characteristics of included studies. [file HUMU-2026-9405486-s031.zip › S23/File S23_AI_Augmented_HHA_Carrier_Detection_Studies_Matrix.docx]

File S23: Comprehensive Study Characteristics Matrix

AI-Augmented HHA Carrier Detection Studies (n=85)

## Study Design Characteristics Matrix

### Geographic Distribution and Settings

| **Region** | **Studies** | **%** | **Settings (Primary)** | **Urban/Rural Mix** |
| --- | --- | --- | --- | --- |
| Middle East | 29 | 34.1% | Tertiary hospitals (65%), Community clinics (25%), Mobile units (10%) | 70%/30% |
| South Asia | 25 | 29.4% | District hospitals (45%), PHCs (35%), Research centers (20%) | 55%/45% |
| Europe/Americas | 18 | 21.2% | University hospitals (75%), Screening centers (15%), Private labs (10%) | 85%/15% |
| Sub-Saharan Africa | 13 | 15.3% | District hospitals (40%), PHCs (35%), Refugee camps (15%), Mobile (10%) | 30%/70% |

### Population Characteristics

| **Characteristic** | **Range** | **Median** | **Notes** |
| --- | --- | --- | --- |
| Age (years) | 18-45 | 28 | All reproductive age |
| Gender distribution | 45-55% female | 52% female | Slightly more females in premarital screening |
| Ethnic diversity | Low-High | Moderate | Highest in African, lowest in Middle Eastern studies |
| Consanguinity rate | 15-65% | 35% | Higher in Middle East/S Asia |
| Literacy rate | 45-99% | 78% | Impacts consent and understanding |

### AI Model Implementation Details

| **Model Type** | **Hardware Used** | **Software Stack** | **Training Time** | **Inference Time** |
| --- | --- | --- | --- | --- |
| Deep Learning | GPU servers (65%), Cloud (25%), Edge devices (10%) | TensorFlow (45%), PyTorch (35%), Custom (20%) | 4-48 hours | 0.5-5 seconds |
| Random Forest | CPU servers (80%), Desktop (20%) | scikit-learn (70%), R (20%), Weka (10%) | 0.5-2 hours | <1 second |
| XAI | Mixed (GPU/CPU) | SHAP (40%), LIME (30%), Integrated (30%) | 2-24 hours | 1-3 seconds |
| Ensemble | CPU servers (90%) | XGBoost (50%), Voting (30%), Stacking (20%) | 1-6 hours | 1-2 seconds |
| Federated Learning | Distributed nodes | PySyft (40%), TensorFlow Federated (35%), Custom (25%) | Variable | Variable |

### Data Collection and Processing Pipeline

| **Stage** | **Methods Used** | **Quality Control** | **Common Issues** |
| --- | --- | --- | --- |
| Sample collection | Venipuncture (85%), Fingerprick (15%) | Standard protocols (70%), Variable (30%) | Hemolysis (12%), Clotting (8%), Volume issues (5%) |
| Laboratory analysis | Automated analyzers (75%), Manual (25%) | Internal QC (85%), External QC (45%) | Calibration drift (15%), Reagent issues (10%) |
| Data preprocessing | Normalization (90%), Imputation (65%), Augmentation (40%) | Outlier detection (70%), Consistency checks (55%) | Missing values (20%), Extreme values (8%), Unit inconsistencies (5%) |
| Feature engineering | Manual (45%), Automated (35%), Hybrid (20%) | Correlation analysis (60%), Importance ranking (50%) | Overfitting risk (25%), Multicollinearity (15%) |
| Model validation | k-fold CV (55%), Hold-out (30%), Bootstrapping (15%) | Multiple metrics (80%), Statistical testing (60%) | Data leakage (12%), Optimism bias (8%) |

### Ethical and Regulatory Compliance

| **Aspect** | **Compliance Rate** | **Documentation** | **Common Gaps** |
| --- | --- | --- | --- |
| Ethics approval | 92% (78/85) | IRB numbers (80%), Statements (20%) | Community studies often lacking formal approval |
| Informed consent | 88% (75/85) | Written (65%), Verbal/witnessed (35%) | Low literacy adaptations in only 30% |
| Data privacy | 82% (70/85) | Anonymization (75%), Encryption (40%) | Data sharing agreements often vague |
| Algorithmic fairness | 35% (30/85) | Bias assessment (30%), Mitigation (15%) | Most studies don't assess across subgroups |
| Regulatory approval | 12% (10/85) | CE/FDA (8%), Local (4%) | Most are research prototypes |
| Conflict of interest | 95% (81/85) | Declarations (95%) | Usually "none declared" |

### Implementation Challenges Documentation

| **Challenge Category** | **Studies Reporting** | **%** | **Most Affected Regions** |
| --- | --- | --- | --- |
| Infrastructure limitations | 58 | 68.2% | Africa (100%), South Asia (72%) |
| Technical expertise gap | 47 | 55.3% | Africa (85%), South Asia (60%) |
| Data quality issues | 42 | 49.4% | All regions, especially community studies |
| Cultural/religious barriers | 28 | 32.9% | Middle East (45%), South Asia (35%) |
| Cost constraints | 52 | 61.2% | Africa (92%), South Asia (68%) |
| Regulatory hurdles | 32 | 37.6% | Europe (55%), Americas (50%) |
| Patient acceptance | 24 | 28.2% | All regions, variable |
| Integration with existing systems | 38 | 44.7% | Tertiary centers (65%) |

### Study Quality Indicators

| **QUADAS-2 Domain** | **Low Risk** | **Some Concerns** | **High Risk** | **Unclear** |
| --- | --- | --- | --- | --- |
| Patient Selection | 38 (44.7%) | 22 (25.9%) | 25 (29.4%) | 0 |
| Index Test | 85 (100%) | 0 | 0 | 0 |
| Reference Standard | 85 (100%) | 0 | 0 | 0 |
| Flow & Timing | 45 (52.9%) | 15 (17.6%) | 25 (29.4%) | 0 |

### Timeline and Evolution Patterns

| **Period** | **Characteristics** | **Technological Focus** | **Implementation Context** |
| --- | --- | --- | --- |
| 2020-2022 (n=18) | Proof-of-concept, single-center, algorithm development | Basic ML, traditional classifiers | Academic settings, controlled environments |
| 2023 (n=24) | Validation studies, multi-feature integration | Deep learning emergence, feature engineering | Hospital labs, some community pilots |
| 2024 (n=28) | Comparative studies, real-world testing | XAI, ensemble methods, mobile AI | Mixed settings, more real-world |
| 2025 (n=15) | Implementation research, equity focus | Federated learning, edge AI, blockchain | Diverse contexts including conflict zones |

## Special Population Considerations

### Conflict Zone Studies (n=3)

| **Study** | **Context** | **Adaptations** | **Challenges** | **Success Factors** |
| --- | --- | --- | --- | --- |
| Study 54 (Yemen) | Active conflict, mobile clinics | Fingerprick samples, offline AI, community health workers | Security risks, supply chain disruptions | Community trust, simplified protocols |
| Study 56 (Somalia) | Refugee camps, pastoral communities | Mobile microscopy, solar power, local language interfaces | Infrastructure absence, population mobility | Cultural adaptation, local leadership |
| Study 58 (Sudan) | Post-conflict, damaged infrastructure | Hybrid cloud-edge, battery backup, paper backup systems | Intermittent power, technician shortages | Resilience planning, task shifting |

### Low-Resource Settings (n=22)

| **Characteristic** | **Adaptation Strategies** | **Effectiveness** | **Scalability** |
| --- | --- | --- | --- |
| Power instability | Solar charging (45%), Battery backup (60%), Manual fallback (25%) | High (prevents 80% of disruptions) | Moderate (cost barrier) |
| Internet limitations | Offline models (70%), Periodic sync (40%), SMS-based (15%) | Good (maintains 90% functionality) | High (low cost) |
| Technician shortage | Task shifting (55%), Simplified interfaces (65%), Remote support (35%) | Moderate (requires training investment) | Variable |
| Cost constraints | Open-source software (75%), Recycled devices (30%), Public funding (45%) | Good (reduces costs 40-60%) | High with support |

### High-Consanguinity Populations (n=29)

| **Aspect** | **Prevalence Impact** | **Screening Approach** | **Ethical Considerations** |
| --- | --- | --- | --- |
| Carrier rate | 2-3x higher than general population | Family-based approaches, cascade testing | Privacy concerns, family dynamics |
| Variant diversity | Limited (founder effects) | Targeted variant panels, simplified algorithms | May miss rare variants |
| Acceptance | Higher due to community awareness | Community engagement, religious leader involvement | Stigma management crucial |
| Follow-up | Challenging (family networks) | Integrated genetic counseling, family sessions | Confidentiality complexities |

## Data Sharing and Reproducibility

### Availability Metrics

| **Resource Type** | **Available** | **Partially Available** | **Not Available** |
| --- | --- | --- | --- |
| Raw data | 35% (30/85) | 25% (21/85) | 40% (34/85) |
| Code/algorithm | 28% (24/85) | 20% (17/85) | 52% (44/85) |
| Model weights | 15% (13/85) | 10% (9/85) | 75% (63/85) |
| Preprocessing scripts | 22% (19/85) | 18% (15/85) | 60% (51/85) |
| Validation splits | 40% (34/85) | 25% (21/85) | 35% (30/85) |

### Common Reproducibility Barriers

1. Proprietary algorithms (commercial systems)
2. Data privacy restrictions (especially genetic data)
3. Incomplete documentation of preprocessing steps
4. Hardware/software dependencies
5. Lack of standardization in evaluation metrics

### Best Practice Examples

- - Study 81: Complete code, data, and containerized environment
- - Study 62: Federated learning implementation with privacy preservation
- - Study 45: Extensive documentation and validation protocols
- - Study 77: Multi-center reproducibility assessment

## Future Research Directions Identified

### Priority Gaps from Included Studies

| **Gap Category** | **Studies Mentioning** | **%** | **Recommended Approaches** |
| --- | --- | --- | --- |
| African representation | 42 | 49.4% | Federated learning hubs, targeted funding |
| Real-world validation | 38 | 44.7% | Pragmatic trials, implementation research |
| Long-term outcomes | 25 | 29.4% | Longitudinal studies, registry linkages |
| Cost-effectiveness | 32 | 37.6% | Economic evaluations, budget impact analysis |
| Ethical frameworks | 18 | 21.2% | Co-design with communities, equity assessments |
| Integration pathways | 35 | 41.2% | Health system research, policy analysis |

### Methodological Improvements Needed

1. Standardized reporting (STARD-AI compliance)
2. Bias assessment protocols across diverse populations
3. Data quality metrics for routine clinical samples
4. Implementation science frameworks for scale-up
5. Mixed-methods approaches combining quantitative and qualitative

**——————————————————————————————————————————————————**

*This matrix synthesizes characteristics across all 85 included studies, providing a comprehensive overview of the evidence base for AI-augmented HHA carrier detection.*
