# Supplementary material for: AI‐Augmented Hematological Signatures for Equitable Detection of Hereditary Hemolytic Anemia Carriers: A Global Systematic Review and Meta‐Analysis
Source: Hum Mutat. 2026 Jun 27;2026:9405486. doi: 10.1155/humu/9405486 (PMC13309745; doi:10.1155/humu/9405486)
Supplement: Supplementary file 25 — Supporting Information 25 File S24: Implementation framework details. [file HUMU-2026-9405486-s028.docx]

**File S24: Implementation Framework Details**

| Component | Tier 1: Basic | Tier 2: Intermediate | Tier 3: Advanced |
| --- | --- | --- | --- |
| **Setting** | Rural clinics, conflict zones, no internet | District hospitals, basic infrastructure | Tertiary centers, urban hospitals |
| **Infrastructure** | Solar power, 48-hour battery, offline only | Intermittent internet, hybrid cloud-edge | Stable internet, cloud integration |
| **Device Requirements** | Edge AI device ($120-200), smartphone adapter | Tablet/laptop ($300-500), basic server | Dedicated workstation ($800+), server cluster |
| **AI Model** | TinyML (<5 MB), pre-trained, no updates | Lightweight (20-50 MB), quarterly updates | Full model (100+ MB), continuous learning |
| **Tests Available** | CBC + basic smear analysis | CBC + advanced smear + limited HPLC | Full test panel + integration with LIS |
| **Training Needs** | 1-day basic training for CHWs | 3-day training for lab technicians | 1-week training for specialists |
| **Cost per Screen** | $3.50-4.00 | $4.50-6.00 | $8.00-12.00 |
| **Savings per Screen** | $5.20 | $8.50 | $12.30 |
| **Screening Capacity** | 20-50/day | 100-200/day | 300-500/day |
| **Quality Assurance** | Monthly manual review | Weekly automated checks | Real-time monitoring |
| **Data Management** | Local storage, manual backup | Cloud backup, basic analytics | Integrated EMR, advanced analytics |
| **Maintenance** | Community health worker basic | Technician quarterly visits | IT support, remote monitoring |
| **Scalability** | Village-level deployment | District-level network | Regional/national system |
| **Key Performance Indicators** | Uptime >90%, accuracy >85% | Uptime >95%, accuracy >90% | Uptime >99%, accuracy >95% |

**Implementation Timeline:**

| Phase | Duration | Activities | Success Metrics |
| --- | --- | --- | --- |
| Phase 1: Planning | Months 1-3 | Needs assessment, site selection, stakeholder engagement | 5 pilot sites identified, local buy-in secured |
| Phase 2: Procurement | Months 4-6 | Device procurement, infrastructure setup, initial training | Devices deployed, staff trained, basic infrastructure ready |
| Phase 3: Pilot | Months 7-12 | Pilot implementation, data collection, initial evaluation | 1,000+ screens completed, accuracy >85%, user feedback positive |
| Phase 4: Scale-up | Months 13-24 | Expand to 20+ sites, optimize workflows, train trainers | 10,000+ screens, cost-effectiveness demonstrated |
| Phase 5: Integration | Months 25-36 | Health system integration, policy adoption, sustainability planning | National guidelines adopted, local maintenance capacity |

**Monitoring and Evaluation Framework:**

| Indicator | Measurement | Target | Frequency |
| --- | --- | --- | --- |
| **Technical** | System uptime | 95% | Daily |
|  | Model accuracy | 90% | Weekly |
|  | Processing time | <2 minutes | Per batch |
| **Clinical** | Detection rate | 90% | Monthly |
|  | False positive rate | <10% | Monthly |
|  | Confirmatory test reduction | 20% | Quarterly |
| **Operational** | Screens per day | Site-specific | Daily |
|  | Cost per screen | <$5.00 | Monthly |
|  | User satisfaction | 4/5 | Quarterly |
| **Impact** | Carriers detected | Per targets | Annually |
|  | Affected births prevented | Calculated | Annually |
|  | Cost savings | ROI > 2:1 | Annually |

**Risk Mitigation Strategies:**

Technical Risks: Backup devices, offline capability, regular maintenance

Human Resources: Task shifting, simplified interfaces, ongoing training

Financial Risks: Phased funding, cost-sharing, government partnerships

Sustainability: Local capacity building, revenue generation, policy integration

Ethical Risks: Bias monitoring, privacy protection, community engagement

**Success Factors:**

Community Engagement: Local leadership involvement, cultural adaptation

Government Support: Policy alignment, integration with national programs

Appropriate Technology: Match to infrastructure, scalable solutions

Capacity Building: Training, mentorship, continuous support

Monitoring & Adaptation: Regular feedback, iterative improvement
