# Supplementary material for: AI‐Augmented Hematological Signatures for Equitable Detection of Hereditary Hemolytic Anemia Carriers: A Global Systematic Review and Meta‐Analysis
Source: Hum Mutat. 2026 Jun 27;2026:9405486. doi: 10.1155/humu/9405486 (PMC13309745; doi:10.1155/humu/9405486)
Supplement: Supplementary file 26 — Supporting Information 26 Table S1: QUADAS‐2 risk of bias summary across all studies. Table S2: Overview of characteristics of included studies. Table S3: AI model type classification. Table S4: AI model card: HHA screening framework. Table S5: Complete search strategies. Table S6: Key for visual examples of AI‐interpreted blood smears. Table S7: Summary of 2 × 2 contingency data across all studies (verified). Table S8: Metaregression analysis of sources of heterogeneity. Table S9: Comparative performance of AI models across settings. Table S10: Detailed cost–benefit analysis by implementation scenario. Table S11: Sensitivity analysis results with data verification. Table S12: Publication bias assessment results. Table S13: Subgroup analysis summary. Table S14: Heterogeneity analysis results. Table S15: Variables extracted for data management. Table S16: Subgroup analysis by geographic region. Table S17: Impact of infrastructure gaps on cost savings. Table S18: Diagnostic accuracy by disease category (HHA type). [file HUMU-2026-9405486-s027.docx]

# Supplementary Tables

**Table S1: QUADAS-2 Risk of Bias Summary Across All Studies**

| **Risk of Bias Domain** | **Low Risk** | **Some Concerns** | **High Risk** | **Unclear** | **Total** |
| --- | --- | --- | --- | --- | --- |
| **Patient Selection** | 38 (44.7%) | 22 (25.9%) | 25 (29.4%) | 0 (0%) | 85 |
| **Index Test (AI Model)** | 85 (100%) | 0 (0%) | 0 (0%) | 0 (0%) | 85 |
| **Reference Standard** | 85 (100%) | 0 (0%) | 0 (0%) | 0 (0%) | 85 |
| **Flow and Timing** | 45 (52.9%) | 15 (17.6%) | 25 (29.4%) | 0 (0%) | 85 |
| **Overall Risk** | 32 (37.6%) | 38 (44.7%) | 15 (17.6%) | 0 (0%) | 85 |

**Key Findings**

**Index Test** domain showed 100% low risk (all AI models properly validated).

**Patient Selection** had the highest high risk (29.4%), mainly due to convenience sampling.

**Flow and Timing** issues were noted in 29.4% of studies (interval between tests unclear).

African studies had a higher overall risk (46.2% high risk vs 12.5% in Europe).

**Regional Variations in Overall High Risk**

| **Region** | **High Risk Percentage** |
| --- | --- |
| Europe/Americas | 12.5% |
| Middle East | 17.2% |
| South Asia | 20.0% |
| Sub-Saharan Africa | 46.2% |

**Impact on Estimates**

**High risk studies:** Sensitivity 91.2%, Specificity 90.1%

**Low risk studies:** Sensitivity 93.5%, Specificity 92.3%

**Difference:** $\Delta$Sensitivity +2.3% (p=0.04), $\Delta$Specificity +2.2% (p=0.05)

**Table S2: Characteristics of Included Studies Overview**

| **Characteristic** | **Category** | **Number of Studies** | **Percentage** | **Total Participants** |
| --- | --- | --- | --- | --- |
| **Publication Year** | 2020-2022 | 18 | 21.2% | 24,850 |
|  | 2023-2025 | 67 | 78.8% | 108,648 |
| **Geographic Region** | Middle East | 29 | 34.1% | 45,220 |
|  | South Asia | 25 | 29.4% | 38,950 |
|  | Europe/Americas | 18 | 21.2% | 31,280 |
|  | Sub-Saharan Africa | 13 | 15.3% | 18,048 |
| **AI Model Type** | Deep Learning | 28 | 32.9% | 46,320 |
|  | Random Forest | 18 | 21.2% | 29,850 |
|  | Ensemble Methods | 18 | 21.2% | 27,930 |
|  | Explainable AI (XAI) | 15 | 17.6% | 21,760 |
|  | Federated Learning | 6 | 7.1% | 7,638 |
| **Test Combination** | CBC Only | 48 | 56.5% | 68,450 |
|  | CBC + Blood Smear | 32 | 37.6% | 52,980 |
|  | CBC + HPLC | 5 | 5.9% | 12,068 |
| **Reference Standard** | Genetic Testing | 45 | 52.9% | 69,850 |
|  | HPLC | 35 | 41.2% | 52,648 |
|  | Capillary Electrophoresis | 5 | 5.9% | 11,000 |
| **Sample Size** | 100-500 | 28 | 32.9% | 8,960 |
|  | 501-1000 | 25 | 29.4% | 18,750 |
|  | 1001-5000 | 29 | 34.1% | 68,420 |
|  | >5000 | 3 | 3.5% | 37,368 |
| **Prevalence Range** | <5% | 32 | 37.6% | 51,280 |
|  | 5-10% | 25 | 29.4% | 39,850 |
|  | 10-15% | 18 | 21.2% | 29,640 |
|  | >15% | 10 | 11.8% | 12,728 |

**Summary Statistics:**

**Total:** 85 studies, 133,498 participants

**Mean Sample Size:** 1,570 (Range: 120-5,410)

**Mean Prevalence:** 8.7% (Range: 2.1-22.5%)

## Table S3: AI Model Type Classification

| Model Type | Algorithms Included | Key Features | Use Cases in HHA Screening | Implementation Examples |
| --- | --- | --- | --- | --- |
| Deep Learning | CNN, RNN, Transformers, Autoencoders | High accuracy, automatic feature extraction, handles complex patterns | Blood smear image analysis, complex pattern recognition | Study1, Study4, Study17, Study27 |
| Explainable AI (XAI) | SHAP, LIME, Decision Trees, Random Forest | Interpretable predictions, feature importance, clinical trust | Risk explanation, clinician acceptance, bias detection | Study9, Study14, Study19, Study34 |
| Federated Learning | Decentralized training, secure aggregation | Privacy-preserving, multi-center collaboration, data sovereignty | Cross-institutional studies, conflict zones, data-sensitive settings | Study2, Study11, Study16, Study31 |
| Edge AI | Mobile-optimized models, on-device inference | Offline capability, low latency, minimal connectivity requirements | Remote areas, point-of-care screening, low-resource settings | Study54, Study58, Study78 |
| Mobile CNN | Lightweight CNN architectures | Smartphone compatibility, real-time processing | Community health workers, field screening | Study57, Study65, Study70, Study73 |
| Blockchain AI | Secure validation, immutable records | Audit trail, data integrity, regulatory compliance | High-security settings, regulatory requirements | Study66 |
| Ensemble Methods | XGBoost, Gradient Boosting, Stacking | Robustness, reduced overfitting, improved generalization | Combined test interpretation, risk stratification | Study5, Study6, Study10, Study15 |

**Performance Comparison:**

- Highest Sensitivity: Deep Learning (95.1%)
- Highest Specificity: XAI (94.3%)
- Best Balance: Ensemble Methods (93.8% Sensitivity, 92.5% Specificity)
- Most Resource-Efficient: Edge AI (89.2% Sensitivity, 90.1% Specificity)

## Table S4: AI Model Card - HHA Screening Framework

| Component | Specification | Details/Values |
| --- | --- | --- |
| **Intended Use** | Target Population | Asymptomatic adults (18-45 years) in premarital/community screening |
|  | Clinical Setting | Primary care clinics, community health centers, mobile screening units |
|  | Geographic Scope | Global, with specific adaptations for high-prevalence regions |
| **Performance Metrics** | Overall Sensitivity | 92.8% (95% CI: 91.3-94.1) |
|  | Overall Specificity | 91.5% (95% CI: 89.7-93.0) |
|  | AUC | 0.93 (95% CI: 0.91-0.95) |
|  | Performance Disparity | ΔSensitivity: -12.6% between Middle East and Africa (p<0.001) |
|  | Data Representation Gap | Only 12% training data from Africa despite 40% disease burden |
| **Technical Specifications** | Hardware Requirements | Minimum: 4GB RAM, 2GHz CPU; Recommended: 8GB RAM, dedicated GPU |
|  | Software Dependencies | Python 3.8+, TensorFlow 2.8+, OpenCV, scikit-learn |
|  | Model Size | 45 MB (full), 5 MB (mobile-optimized) |
|  | Inference Time | <2 seconds per sample on recommended hardware |
| **Ethical Constraints** | Privacy Requirements | GDPR/HIPAA compliance, data anonymization, federated learning support |
|  | Informed Consent | Tiered approach (written/verbal/witnessed) based on literacy |
|  | Cultural Adaptations | Local language support, community leader involvement |
| **Implementation Recommendations** | Infrastructure Tier 1 (Basic) | Offline-capable edge devices, solar power, 48-hour battery |
|  | Infrastructure Tier 2 (Intermediate) | Hybrid cloud-edge, periodic updates, basic connectivity |
|  | Infrastructure Tier 3 (Advanced) | Full integration, real-time monitoring, continuous learning |
| **Bias Mitigation Strategies** | Data Diversity | Minimum 30% representation from underrepresented populations |
|  | Regular Auditing | Quarterly performance monitoring across demographic subgroups |
|  | Model Updating | Federated learning updates every 6 months |

## Table S5: Complete Search Strategies

| Database | Search Date | Search Syntax | Results | Notes |
| --- | --- | --- | --- | --- |
| PubMed/MEDLINE | 2025-06-30 | (“premarital screening” OR “carrier detection”) AND (“thalassemia” OR “sickle cell” OR “hemoglobinopathy”) AND (“artificial intelligence” OR “machine learning” OR “deep learning”) AND (“complete blood count” OR “blood smear” OR “CBC”) | 452 | Primary biomedical database |
| Embase | 2025-06-30 | ‘premarital screening’/exp AND (‘thalassemia’/exp OR ‘sickle cell anemia’/exp) AND (‘artificial intelligence’/exp OR ‘machine learning’/exp) AND (‘blood count’/exp OR ‘blood smear’/exp) | 387 | Includes conference abstracts |
| Scopus | 2025-06-30 | TITLE-ABS-KEY((premarital OR carrier) AND (screening OR detection) AND (thalassemia OR “sickle cell”) AND (“artificial intelligence” OR “machine learning”) AND (“blood count” OR “blood smear”)) | 321 | Multidisciplinary coverage |
| Cochrane CENTRAL | 2025-06-30 | (“premarital screening” OR “carrier screening”) AND (thalassemia OR “sickle cell disease”) AND (“artificial intelligence” OR AI) | 45 | Focus on systematic reviews |
| IEEE Xplore | 2025-06-30 | (“Abstract”:“thalassemia” OR “Abstract”:“sickle cell”) AND (“Abstract”:“AI” OR “Abstract”:“machine learning”) AND (“Abstract”:“screening” OR “Abstract”:“detection”) | 89 | Technical/AI algorithms |
| African Journals Online (AJOL) | 2025-06-30 | (premarital OR carrier) AND (thalassemia OR “sickle cell”) AND (screening) | 67 | African-specific research |
| LILACS | 2025-06-30 | (detección de portadores OR tamizaje premariatal) AND (talasemia OR anemia de células falciformes) AND (inteligencia artificial) | 42 | Latin American literature |
| GulfBase/IMEMR | 2025-06-30 | (فحص ما قبل الزواج OR كشف الحاملين) AND (الثلاسيميا OR فقر الدم المنجلي) AND (الذكاء الاصطناعي) | 58 | Middle Eastern databases |
| Gray Literature | 2025-06-30 | WHO IRIS, CDC reports, conference proceedings, theses | 112 | Unpublished/ongoing research |

**Search Strategy Notes:**

- Date Range: 2010-2025
- Language Filters: English, Arabic, French, Spanish
- Study Design Filters: Diagnostic accuracy studies, validation studies, clinical trials
- Inclusion Criteria: Asymptomatic adults, AI/ML models, routine tests (CBC/blood smear/ESR)
- Exclusion Criteria: Symptomatic patients, non-premarital contexts, neonatal screening

## Table S6: Visual Examples Key for AI-Interpreted Blood Smears

| Figure | Description | AI Interpretation | Clinical Significance |
| --- | --- | --- | --- |
| **Figure A** | Normal blood smear | No abnormal features detected | Baseline for comparison |
|  | RBC Morphology | Uniform size and shape (normocytic, normochromic) | Excludes hematological disorders |
|  | AI Confidence | 98.7% probability of normal | High reliability |
| **Figure B** | Thalassemia carrier smear | Microcytosis (MCV <80 fL), hypochromia, target cells | Suggests α- or β-thalassemia trait |
|  | Key Features | Mentzer index <13, RDW normal/mildly increased | Differentiates from iron deficiency |
|  | AI Annotations | Red circles: target cells; Blue arrows: microcytes | Visual guidance for clinicians |
| **Figure C** | Sickle cell features | Sickled cells, hemoglobin C crystals, Howell-Jolly bodies | Indicates hemoglobinopathy |
|  | Diagnostic Markers | 15% sickled cells, SC crystals present | Specific for HbS/HbC variants |
|  | Severity Grading | Moderate sickling (Grade 2/4) | Correlates with clinical severity |
| **Figure D** | Comparative analysis | Side-by-side: Manual vs AI interpretation | Demonstrates AI advantage |
|  | Manual Assessment | Variability: κ=0.65 between hematologists | Highlights subjectivity issue |
|  | AI Assessment | Consistency: 99.2% agreement across runs | Shows AI reproducibility |
|  | Time Comparison | Manual: 8.5 minutes vs AI: 1.2 minutes | Efficiency improvement |

**Interpretation Guidelines:**

1. Normal Range: <5% abnormal cells, MCV 80-100 fL, MCH 27-33 pg
2. Thalassemia Suspicion: MCV <80 fL, MCH <27 pg, normal RDW, target cells
3. Sickle Cell Indicators: >5% sickled cells, SC crystals, Howell-Jolly bodies
4. AI Confidence Thresholds: >95%: High confidence; 80-95%: Moderate; <80%: Requires review

**Table S7: Summary of 2x2 Contingency Data Across All Studies (Verified)**

| Statistic | Mean | Median | Range | IQR | SD |
| --- | --- | --- | --- | --- | --- |
| True Positives (TP) | 108.4 | 65 | 11-532 | 35-155 | 112.3 |
| False Positives (FP) | 96.8 | 62 | 8-468 | 35-138 | 94.5 |
| True Negatives (TN) | 1,312.5 | 922 | 305-4,870 | 642-2,045 | 1,245.8 |
| False Negatives (FN) | 32.6 | 19 | 0-253 | 10-55 | 38.4 |
| Sensitivity | 0.928 | 0.932 | 0.792-0.985 | 0.908-0.958 | 0.045 |
| Specificity | 0.915 | 0.919 | 0.830-0.972 | 0.889-0.943 | 0.038 |
| Prevalence | 0.087 | 0.065 | 0.021-0.225 | 0.043-0.117 | 0.052 |

This table summarizes the key statistical metrics derived from the **VERIFIED** 2x2 contingency data across all included studies (n=85). All data has been cross-checked for consistency between reported sensitivity/specificity and calculated values from TP/FP/TN/FN.

**Data Quality Indicators (Updated)**

The following indicators reflect the quality and consistency of the verified data used in the analysis:

**Complete 2x2 data:** 85 studies (100%)

**Data consistency after verification:** 100% (85/85 studies)

**Required correction:** 2 studies (Study 4, Study 7) - now verified

**Missing AUC data:** 12.9% (11/85 studies) - imputed from sensitivity/specificity

**Verification Protocol:**

All TP, FP, TN, FN values were verified using: Sensitivity = TP/(TP+FN), Specificity = TN/(TN+FP)

Discrepancies were resolved by checking original study reports or logical imputation

Final dataset includes 85 studies with 100% consistency between raw counts and reported metrics

**Meta-Regression Analysis of Diagnostic Accuracy Studies**

**Table S8: Meta-Regression Analysis of Sources of Heterogeneity**

| Covariate | Category | Coefficient (95% CI) | p-value | % Variance Explained | Interpretation |
| --- | --- | --- | --- | --- | --- |
| **Sample Size** (per 100 increase) | Continuous | -0.012 (-0.018 to -0.006) | 0.001 | 18.2% | Larger studies show slightly lower estimates (small-study effect) |
| **AI Model Type** | Deep Learning vs Others | 0.042 (0.018 to 0.066) | 0.003 | 15.7% | Deep Learning has 4.2% higher sensitivity |
| **Geographic Region** | Africa vs Non-Africa | -0.085 (-0.112 to -0.058) | <0.001 | 22.4% | African studies 8.5% lower sensitivity |
| **Reference Standard** | Genetic vs Other | 0.028 (0.008 to 0.048) | 0.02 | 8.3% | Genetic reference gives 2.8% higher estimates |
| **Test Combination** | CBC+Smear vs CBC Alone | 0.051 (0.031 to 0.071) | <0.001 | 20.1% | Adding blood smear improves sensitivity 5.1% |
| **Study Quality** | QUADAS-2 Low Risk | 0.019 (0.002 to 0.036) | 0.04 | 5.8% | Better quality studies 1.9% higher |
| **Publication Year** (per year) | Continuous | 0.005 (0.001 to 0.009) | 0.03 | 4.5% | Recent studies 0.5% higher per year |
| **Prevalence** (per 10% increase) | Continuous | -0.016 (-0.024 to -0.008) | 0.002 | 11.2% | Higher prevalence settings 1.6% lower sensitivity |

**Multivariate Model Results**

The multivariate model provided a strong fit for the data, explaining a significant portion of the observed heterogeneity.

**R² = 85.2%** (cumulative variance explained by all covariates)

**Residual heterogeneity:** τ² = 0.008 (I² = 32%)

**Model fit:** Q = 45.2, p = 0.12 (adequate fit)

**Key Findings from Meta-Regression**

The analysis identified several key factors influencing the diagnostic accuracy estimates:

**Geographic region is strongest predictor** (22.4% variance)

**Test combination second most important** (20.1% variance)

**African performance gap persists** even after adjusting for other factors

**Deep Learning superiority confirmed** independent of other factors

**Regional Subgroup Meta-Regressions**

Subgroup analysis by geographic region revealed differences in the strongest predictors of heterogeneity:

| Region | Strongest Predictor | Coefficient | p-value |
| --- | --- | --- | --- |
| **Africa** | Infrastructure quality | 0.128 (0.085-0.171) | <0.001 |
| **Middle East** | Model type | 0.055 (0.032-0.078) | 0.002 |
| **South Asia** | Sample size | -0.018 (-0.027 to -0.009) | 0.001 |
| **Europe/Americas** | Reference standard | 0.034 (0.018-0.050) | 0.003 |

**Clinical Implications**

The findings have several important implications for clinical practice and future research:

Geographic disparities not fully explained by study quality or methods

Blood smear addition valuable across all settings

Deep Learning performs best regardless of region

Need for region-specific model calibration evident

**Statistical Methods**

The meta-regression was conducted using the following statistical procedures:

Random-effects meta-regression using restricted maximum likelihood

Knapp-Hartung adjustment for small number of studies

Multivariate model with forward selection (p<0.10 for entry)

Variance inflation factors <2.0 for all covariates

**Table S9. Comparative Performance of AI Models Across Settings**

| **Deep Learning** | **28** | **95.1% (93.2-96.5)** | **92.8% (90.1-94.8)** | **0.96 (0.94-0.98)** | **Blood smear image analysis** | **Low-resource mobile settings** | **High accuracy, automatic feature extraction** | **Computationally intensive, "black box"** |
| --- | --- | --- | --- | --- | --- | --- | --- | --- |
| **Explainable AI (XAI)** | 15 | 94.3% (92.0-96.0) | 94.3% (92.0-96.0) | 0.95 (0.93-0.97) | Clinical decision support | Large-scale screening | Interpretable, builds clinical trust | Lower sensitivity than DL |
| **Random Forest** | 18 | 93.5% (91.2-95.3) | 91.8% (89.5-93.7) | 0.94 (0.92-0.96) | Tabular CBC data | Complex morphology | Robust, handles missing data | Limited with image data |
| **Ensemble Methods** | 18 | 93.8% (91.5-95.5) | 92.5% (89.9-94.5) | 0.95 (0.93-0.97) | Combined test interpretation | Rapid screening needs | Reduced overfitting, stable | Complex implementation |
| **Federated Learning** | 6 | 92.8% (90.1-94.9) | 90.7% (87.9-92.9) | 0.94 (0.91-0.96) | Multi-center studies | Small single centers | Privacy-preserving, collaborative | Lower individual center performance |
| **Edge/Mobile AI** | 4 | 89.2% (86.5-91.5) | 90.1% (87.3-92.4) | 0.90 (0.87-0.93) | Remote/conflict zones | High-throughput labs | Offline capability, low latency | Reduced accuracy |
| **Region** | Best Model | Sensitivity | Specificity | Recommended Model |  |  |  |  |
| **Sub-Saharan Africa** | XAI | 88.5% | 91.2% | XAI + Edge AI hybrid |  |  |  |  |
| **Middle East** | Deep Learning | 95.8% | 94.1% | Deep Learning |  |  |  |  |
| **South Asia** | Ensemble | 93.2% | 92.8% | Ensemble or Random Forest |  |  |  |  |
| **Europe/Americas** | Deep Learning | 94.5% | 93.7% | Deep Learning or XAI |  |  |  |  |
| **Conflict Zones** | Edge AI | 87.3% | 89.5% | Edge AI with fallback |  |  |  |  |
| **Model Type** | Minimum Hardware | Training Data Needed | Inference Time | Cost/Implementation |  |  |  |  |
| **Deep Learning** | GPU (8GB+ RAM) | >10,000 labeled images | 2-5 seconds | High ($$$) |  |  |  |  |
| **XAI** | CPU (4GB RAM) | 1,000-5,000 samples | 1-3 seconds | Moderate ($$) |  |  |  |  |
| **Random Forest** | CPU (2GB RAM) | 500-2,000 samples | <1 second | Low ($) |  |  |  |  |
| **Ensemble** | CPU (4GB RAM) | 2,000-5,000 samples | 1-2 seconds | Moderate ($$) |  |  |  |  |
| **Federated** | Distributed nodes | Collaborative pooling | Variable | High (infrastructure) |  |  |  |  |
| **Edge AI** | Smartphone/Edge | 500-1,000 samples | <1 second | Low-Moderate ($-$$) |  |  |  |  |
| **Clinical Setting** | Recommended Model | Alternative | Rationale |  |  |  |  |  |
| **Tertiary hospital** | Deep Learning | XAI | High accuracy needed |  |  |  |  |  |
| **District hospital** | XAI or Ensemble | Random Forest | Balance of accuracy/explainability |  |  |  |  |  |
| **Primary clinic** | Random Forest | Edge AI | Simple, interpretable |  |  |  |  |  |
| **Mobile screening** | Edge AI | Mobile CNN | Offline capability essential |  |  |  |  |  |
| **Multi-center network** | Federated Learning | Ensemble | Privacy, data sovereignty |  |  |  |  |  |
| **Conflict zone** | Edge AI + Fallback | Mobile AI | Resilience, offline operation |  |  |  |  |  |

For Africa: Lightweight XAI models optimized for local genotypes

Global: Standardized benchmarking across model types

Low-resource: Edge AI with federated learning updates

Clinical: XAI integration with electronic health records

**Table S10: Detailed Cost-Benefit Analysis by Implementation Scenario**

| Cost Component | Basic Tier (Low Resource) | Intermediate Tier | Advanced Tier | Notes |
| --- | --- | --- | --- | --- |
| **Initial Investment** |  |  |  |  |
| Hardware/Device | $120-200 (Edge AI device) | $300-500 (Tablet+server) | $800-1,200 (Workstation) | One-time cost |
| Software Licensing | $0 (Open source) | $50-100/year | $200-500/year | Annual |
| Installation/Setup | $50-100 | $100-200 | $200-500 | One-time |
| Staff Training | $200 (2 days) | $500 (5 days) | $1,000 (10 days) | One-time |
| **Total Initial Cost** | **$370-600** | **$950-1,300** | **$2,200-3,200** | Per site |
| **Operational Costs (Annual)** |  |  |  |  |
| Maintenance | $50-100 | $100-200 | $200-500 |  |
| Software Updates | $0-50 | $100-200 | $200-400 |  |
| Technical Support | $100-200 | $200-400 | $500-1,000 |  |
| Connectivity | $0 (Offline) | $100-200 | $200-500 |  |
| Quality Control | $50-100 | $100-200 | $200-500 |  |
| **Total Annual Opex** | **$200-450** | **$600-1,200** | **$1,300-2,900** | Per site |
| **Performance Metrics** |  |  |  |  |
| Screening Capacity | 20-50/day | 100-200/day | 300-500/day |  |
| Sensitivity | 85-90% | 90-94% | 94-97% |  |
| Specificity | 88-92% | 92-95% | 95-98% |  |
| Uptime | 90-95% | 95-98% | 98-99% |  |
| **Economic Benefits** |  |  |  |  |
| Cost per Test | $3.50-4.00 | $4.50-6.00 | $8.00-12.00 |  |
| Savings per Test | $5.20 | $8.50 | $12.30 | vs conventional |
| Break-even Volume | 1,250 tests | 850 tests | 700 tests |  |
| ROI Timeline | 24-36 months | 18-24 months | 12-18 months |  |
| **Regional Adaptation Costs** |  |  |  |  |
| Sub-Saharan Africa | +20% (infrastructure) | +15% | +10% |  |
| Middle East | +10% | +5% | Base cost |  |
| South Asia | +15% | +10% | +5% |  |
| Europe/Americas | Base cost | Base cost | Base cost |  |
| **Sensitivity Analysis** |  |  |  |  |
| Best Case (High volume) | ROI: 18 months | ROI: 12 months | ROI: 8 months | 5,000 tests/year |
| Worst Case (Low volume) | ROI: 48 months | ROI: 36 months | ROI: 24 months | 500 tests/year |
| **Public Health Impact** |  |  |  |  |
| Additional carriers detected/year* | 125 | 425 | 1,150 | per 10,000 screened |
| Affected births prevented/year* | 31 | 106 | 288 | per 10,000 screened |
| DALYs averted/year* | 620 | 2,120 | 5,750 | per 10,000 screened |
| **Scalability Factors** |  |  |  |  |
| Ease of Scale-up | High | Moderate | Low |  |
| Local Maintenance | Possible | Requires training | Specialist needed |  |
| Infrastructure Needs | Minimal | Moderate | High |  |
| **Risk Assessment** |  |  |  |  |
| Technical Failure Risk | Moderate | Low | Low |  |
| Adoption Barriers | Low | Moderate | High |  |
| Sustainability | High | Moderate | Low |  |

*Based on 10% carrier rate, Hardy-Weinberg assumptions, and regional performance data.

**Cost-Effectiveness Analysis (CEA):**

| Scenario | Incremental Cost | Incremental Effect (QALYs) | ICER ($/QALY) | WHO Threshold |
| --- | --- | --- | --- | --- |
| Basic vs Conventional | $2,500 | 6.2 QALYs | $403/QALY | Highly cost-effective (<1x GDP) |
| Intermediate vs Basic | $5,000 | 15.0 QALYs | $333/QALY | Highly cost-effective |
| Advanced vs Intermediate | $10,000 | 36.3 QALYs | $275/QALY | Highly cost-effective |

**Budget Impact Analysis (5-year horizon):**

| Population Coverage | Basic Tier | Intermediate | Advanced | Total Budget |
| --- | --- | --- | --- | --- |
| 100,000 people | $2.5M | $3.8M | $5.2M | $11.5M |
| 500,000 people | $9.5M | $15.2M | $22.5M | $47.2M |
| 1,000,000 people | $18.0M | $28.5M | $42.0M | $88.5M |

**Net Monetary Benefit (NMB) Analysis:** - Willingness-to-pay threshold: $5,000/QALY (LMIC setting) - Basic Tier NMB: $28,500 per 10,000 screened - Intermediate Tier NMB: $72,500 per 10,000 screened
- Advanced Tier NMB: $175,000 per 10,000 screened

**Implementation Recommendations:** 1. **Start with Basic Tier** in resource-limited settings 2. **Gradual transition** based on screening volume and infrastructure 3. **Regional hubs** for Intermediate/Advanced tiers to serve multiple sites 4. **Phased rollout** with continuous economic evaluation

**Key Assumptions:** - Discount rate: 3% per year - Time horizon: 10 years - Currency: 2025 USD - All costs adjusted for purchasing power parity (PPP)

## **Table S11: Sensitivity Analysis Results with Data Verification**

| **Analysis Scenario** | **n Studies** | **Sensitivity (95% CI)** | **Specificity (95% CI)** | **AUC (95% CI)** | **Difference from Main** | **p-value** | **Notes** |
| --- | --- | --- | --- | --- | --- | --- | --- |
| **Main Analysis** (All verified data) | **85** | **92.8% (91.3-94.1)** | **91.5% (89.7-93.0)** | **0.93 (0.91-0.95)** | **Reference** | **-** | **All verified data** |
| **Corrected Data Only** (Excluding corrected studies) | 79 | 92.9% (91.4-94.2) | 91.6% (89.8-93.1) | 0.93 (0.91-0.95) | +0.1% | 0.48 | Excluding 6 studies requiring correction |
| **Community Studies Excluded** | 82 | 92.8% (91.3-94.1) | 91.5% (89.7-93.0) | 0.93 (0.91-0.95) | 0.0% | 0.41 | No significant impact |
| **African Studies Only** (Sub-Saharan Africa) | 13 | 89.7% (87.1-91.8) | 88.3% (85.4-90.7) | 0.89 (0.86-0.91) | -3.1% | 0.008 | Significant regional difference |
| **High Bias Excluded** (QUADAS-2 low risk only) | 50 | 93.2% (91.5-94.6) | 92.1% (90.0-93.8) | 0.94 (0.92-0.96) | +0.4% | 0.38 | Slight improvement with better quality |
| **Small Studies Excluded** (Sample size ≥200) | 68 | 93.1% (91.6-94.4) | 91.8% (89.9-93.3) | 0.93 (0.91-0.95) | +0.3% | 0.45 | Minimal effect of study size |
| **Leave-One-Out** (Worst) (Excluding most influential) | 84 | 92.9% (91.4-94.2) | 91.6% (89.8-93.1) | 0.93 (0.91-0.95) | +0.1% | 0.62 | Robust to individual study influence |
| **Genetic Reference Only** (Genetic testing only) | 45 | 93.5% (91.7-95.0) | 92.3% (90.1-94.0) | 0.94 (0.92-0.96) | +0.7% | 0.21 | Slightly higher with genetic gold standard |
| **HPLC Reference Only** (HPLC only) | 35 | 91.8% (89.6-93.5) | 90.4% (87.8-92.5) | 0.91 (0.88-0.93) | -1.0% | 0.15 | Slightly lower with HPLC reference |
| **Data Verification Impact** (Corrected studies only) | 6 | 89.2% (86.5-91.5) | 90.1% (87.3-92.4) | 0.90 (0.87-0.93) | -3.6% | 0.03 | Studies requiring correction performed lower |

**Key to Verification Status:**

✅ Verified-Consistent: Reported metrics matched calculated values from raw 2x2 data

🔄 Verified-Corrected: Discrepancies identified and corrected using standardized protocol

All corrected data available in File S7_CORRECTED.csv

**Robustness Assessment Summary:**

1. Consistency: All sensitivity estimates within 3.6% of main analysis

2. Stability: Leave-one-out analysis shows minimal influence of individual studies (Δ < 0.1%)

3. Data Quality Impact: Corrected studies showed 3.6% lower performance (p=0.03), highlighting importance of data verification

4. Regional Consistency: African representation significantly impacts estimates (3.1% difference, p=0.008)

5. Methodological Robustness: Excluding high-bias studies improves estimates slightly (+0.4%)

**Statistical Notes:**

Main analysis includes all 85 studies with verified/corrected data

Differences calculated as: Δ = (Subgroup estimate - Main estimate)

p-values from Wald tests comparing subgroup vs. main estimates

Confidence intervals calculated using bivariate random-effects model

**Clinical Interpretation of Sensitivity Analyses:**

1. Primary findings are robust to methodological variations and data verification

2. African underrepresentation remains a critical concern (3.1% performance gap)

3. Data quality matters: Studies requiring correction performed 3.6% lower

4. Reference standard choice modestly affects estimates (genetic +0.7%, HPLC -1.0%)

5. Community studies inclusion does not bias results (Δ = 0.0%, p=0.41)

**Recommendations for Future Meta-Analyses:**

1. Implement mandatory data verification protocols

2. Standardize reporting of 2x2 contingency data

3. Increase representation of underrepresented regions

4. Conduct sensitivity analyses for data quality

5. Report both corrected and uncorrected estimates when discrepancies exist

## Table S12: Publication Bias Assessment Results

| Assessment Method | Test Statistic | p-value | Evidence of Bias? | Interpretation |
| --- | --- | --- | --- | --- |
| Deeks’ Funnel Plot Asymmetry | Slope coefficient: 1.85 | 0.03 | Yes | Small-study effects present |
| Egger’s Test | Intercept: 1.42, t=2.89 | 0.005 | Yes | Significant small-study bias |
| Begg’s Test | Kendall’s tau: 0.24 | 0.08 | Borderline | Mild asymmetry |
| Trim-and-Fill Analysis | Estimated missing studies: 8 | 0.04 | Yes | Imputation changes estimates |
| Fail-Safe N | Orwin’s method: 127 | <0.001 | No | Robust to missing studies |
| Contour-Enhanced Funnel Plot | Asymmetry in non-significant region | 0.02 | Yes | Bias not due to statistical significance |

**Impact of Publication Bias:**

| Metric | Observed Estimate | Trim-and-Fill Adjusted | Difference | Impact |
| --- | --- | --- | --- | --- |
| Sensitivity | 92.8% | 91.2% | -1.6% | Minor |
| Specificity | 91.5% | 90.1% | -1.4% | Minor |
| AUC | 0.93 | 0.91 | -0.02 | Minor |
| Between-Study Variance | τ²=0.045 | τ²=0.052 | +0.007 | Increased heterogeneity |

**Sensitivity to Bias:**

| Study Characteristic | Correlation with Effect Size | p-value | Contribution to Bias |
| --- | --- | --- | --- |
| Sample Size | r=-0.38 | 0.002 | Strong |
| Study Quality (QUADAS-2) | r=0.24 | 0.03 | Moderate |
| Publication Year | r=0.18 | 0.11 | Weak |
| Region | r=0.31 | 0.006 | Moderate |
| AI Model Type | r=0.15 | 0.18 | Weak |

**Mitigation Strategies Applied:**

1. Gray Literature Inclusion: 112 gray literature sources
2. Regional Database Search: AJOL, LILACS, GulfBase, IMEMR
3. Language Inclusion: English, Arabic, French, Spanish
4. Conference Proceedings: IEEE, hematology conferences
5. Theses/Dissertations: University repositories

**Conclusion:** Moderate publication bias present but adjusted estimates remain clinically significant.

## Table S13: Subgroup Analysis Summary

| Subgroup | n Studies | Sensitivity (95% CI) | Specificity (95% CI) | AUC | p-value for Difference |
| --- | --- | --- | --- | --- | --- |
| **By AI Model Type** |  |  |  |  |  |
| Deep Learning | 28 | 95.1% (93.2-96.5) | 92.8% (90.1-94.8) | 0.96 | 0.03 |
| Explainable AI | 15 | 94.3% (92.0-96.0) | 94.3% (92.0-96.0) | 0.95 | 0.12 |
| Federated Learning | 12 | 92.8% (90.1-94.9) | 90.7% (87.9-92.9) | 0.94 | 0.45 |
| Ensemble Methods | 18 | 93.8% (91.5-95.5) | 92.5% (89.9-94.5) | 0.95 | 0.28 |
| **By Test Combination** |  |  |  |  |  |
| CBC Only | 48 | 91.5% (89.3-93.3) | 89.2% (86.5-91.5) | 0.90 | Reference |
| CBC + Blood Smear | 32 | 95.7% (94.1-97.0) | 94.1% (92.3-95.5) | 0.97 | <0.001 |
| CBC + HPLC | 5 | 96.2% (93.8-97.8) | 96.0% (93.8-97.5) | 0.98 | <0.001 |
| **By Geographic Region** |  |  |  |  |  |
| Sub-Saharan Africa | 13 | 86.5% (84.2-88.7) | 88.3% (85.4-90.7) | 0.89 | <0.001 |
| Middle East | 29 | 94.8% (92.9-96.2) | 93.5% (91.2-95.3) | 0.96 | Reference |
| South Asia | 25 | 92.3% (90.1-94.0) | 90.8% (88.3-92.8) | 0.93 | 0.04 |
| Europe/Americas | 18 | 93.5% (91.2-95.3) | 92.7% (90.1-94.7) | 0.95 | 0.18 |
| **By Reference Standard** |  |  |  |  |  |
| Genetic Testing | 45 | 93.5% (91.7-95.0) | 92.3% (90.1-94.0) | 0.94 | 0.21 |
| HPLC | 35 | 91.8% (89.6-93.5) | 90.4% (87.8-92.5) | 0.91 | 0.15 |
| Capillary Electrophoresis | 5 | 90.2% (86.8-92.9) | 89.7% (86.2-92.4) | 0.90 | 0.08 |

**Key Findings from Subgroup Analyses:**

1. AI Model Performance: Deep Learning highest sensitivity, XAI highest specificity
2. Test Combination: Blood smear addition improves specificity by 5.5% (p<0.001)
3. Geographic Disparity: Africa shows 8.3% lower sensitivity than Middle East (p<0.001)
4. Reference Standard: Genetic testing yields slightly higher estimates (+1.7%)
5. Consistency: All subgroups show AI superiority over conventional methods

**Clinical Implications:**

- Resource-rich settings: Deep Learning + CBC + Blood Smear (optimal accuracy)
- Resource-limited settings: XAI + CBC (good accuracy with interpretability)
- Remote areas: Edge AI + Mobile CNN (offline capability)
- Multi-center networks: Federated Learning (data privacy)

## Table S14: Heterogeneity Analysis Results

| Outcome Measure | I² Statistic | Tau² | Cochran’s Q (p-value) | Prediction Interval | Interpretation |
| --- | --- | --- | --- | --- | --- |
| Sensitivity | 68% | 0.045 | 264.3 (<0.001) | 85.4-97.2% | Substantial heterogeneity |
| Specificity | 72% | 0.048 | 297.8 (<0.001) | 82.1-96.8% | Substantial heterogeneity |
| AUC | 63% | 0.003 | 226.4 (<0.001) | 0.84-0.98 | Moderate heterogeneity |
| Diagnostic Odds Ratio | 75% | 0.512 | 336.5 (<0.001) | 25.8-316.4 | High heterogeneity |

**Meta-Regression Results (Sources of Heterogeneity):**

| Covariate | Coefficient (95% CI) | p-value | % Variance Explained |
| --- | --- | --- | --- |
| Sample Size (per 100) | -0.012 (-0.018 to -0.006) | 0.001 | 18.2% |
| AI Model Type (Deep vs Other) | 0.042 (0.018 to 0.066) | 0.003 | 15.7% |
| Region (Africa vs Non-Africa) | -0.085 (-0.112 to -0.058) | <0.001 | 22.4% |
| Reference Standard (Genetic vs Other) | 0.028 (0.008 to 0.048) | 0.02 | 8.3% |
| Test Combination (CBC+Smear vs CBC) | 0.051 (0.031 to 0.071) | <0.001 | 20.1% |
| Study Quality (QUADAS-2 Low Risk) | 0.019 (0.002 to 0.036) | 0.04 | 5.8% |
| Publication Year (per year) | 0.005 (0.001 to 0.009) | 0.03 | 4.5% |
| Prevalence (per 10%) | -0.016 (-0.024 to -0.008) | 0.002 | 11.2% |

**Cumulative Variance Explained:** 85.2% (multiple covariates)

**Subgroup Heterogeneity:**

| Subgroup | Within-group I² | Between-group Q (p) | Contribution to Total Heterogeneity |
| --- | --- | --- | --- |
| AI Model Type | 58% | 18.4 (0.001) | 25.3% |
| Geographic Region | 52% | 32.7 (<0.001) | 45.1% |
| Test Combination | 48% | 24.9 (<0.001) | 34.3% |
| Reference Standard | 61% | 8.7 (0.013) | 12.0% |

**Sensitivity to Heterogeneity:**

| Analysis Method | Sensitivity Estimate | Difference from RE Model | Impact of Heterogeneity |
| --- | --- | --- | --- |
| Random Effects (DerSimonian-Laird) | 92.8% | Reference | - |
| Random Effects (Restricted ML) | 92.6% | -0.2% | Minimal |
| Fixed Effect (Inverse Variance) | 94.1% | +1.3% | Substantial |
| Bayesian (Vague Priors) | 92.4% | -0.4% | Minimal |
| Knapp-Hartung Adjustment | 92.8% (wider CI) | 0.0% | More conservative CI |

**Clinical Interpretation of Heterogeneity:**

1. Expected and Meaningful: Reflects real-world variation in AI models, populations, settings
2. Not Random: Systematic differences explain 85% of variance
3. Actionable: Subgroup analyses provide context-specific estimates
4. Not a Limitation: Enhances generalizability when properly analyzed

**Recommendations:**

1. Always report heterogeneity metrics (I², Tau², prediction intervals)
2. Use random-effects models for meta-analysis of diagnostic studies
3. Conduct pre-specified subgroup analyses to explain heterogeneity
4. Interpret with prediction intervals rather than confidence intervals alone
5. Consider Bayesian methods when substantial heterogeneity persists

**Table S15. Variables extracted for data management**

| Data Category | Variables Extracted |
| --- | --- |
| Study Characteristics | Author, year, country, sample size, prevalence |
| AI Model | Algorithm type, input features, validation method |
| Diagnostic Accuracy | Sensitivity, specificity, AUC (with 95% CIs) |
| Clinical Utility | Cost savings, turnaround time, reduction in confirmatory testing |
| Implementation | Infrastructure requirements, reported challenges |

**Table S16. Subgroup Analysis by Geographic Region**

| Region | Sensitivity (95% CI) | Infrastructure Barriers | Algorithm Bias Risk |
| --- | --- | --- | --- |
| Sub-Saharan Africa (n = 13) | Pooled sensitivity 86.5% (95% CI: 84.2–88.7) | 76% | 48% |
| Middle East (n = 29) | 94.8% (92.9–96.2%) | 25% | 60% |
| South Asia (n = 25) | 92.3% (90.1–94.0%) | 55% | 30% |
| Europe/Americas (n = 18) | 93.5% (91.2–95.3%) | 15% | 70% |

% studies reporting infrastructure limitations

**Table S17. demonstrates how infrastructure gaps directly impact cost savings:**

| Region | Avg. Savings (USD) | Device Cost (USD) | Key Barriers |
| --- | --- | --- | --- |
| Sub-Saharan Africa | 5.20 | 120-300 | Power outages (76%) |
| Middle East | 9.80 | 80-200 | Technician training (35%) |
| Europe/Americas | 12.30 | 200-500 | Regulatory compliance (60%) |

**Table S18: Diagnostic Accuracy by Disease Category (HHA Type)**

| Disease Category | n Studies | Pooled Sensitivity (95% CI) | Pooled Specificity (95% CI) | AUC | Key Challenge |
| --- | --- | --- | --- | --- | --- |
| β-thalassemia carriers | 28 | 94.2% (92.5–95.6%) | 93.1% (90.8–94.9%) | 0.96 | MCV/MCH thresholds vary by ethnicity |
| α-thalassemia carriers | 18 | 89.8% (87.1–92.1%) | 90.5% (87.9–92.7%) | 0.91 | Normal HbA2; requires genetic confirmation |
| Sickle cell trait (HbAS) | 22 | 93.5% (91.2–95.3%) | 92.8% (90.1–94.9%) | 0.95 | HbS quantification by HPLC needed |
| HbC carriers | 8 | 88.2% (84.5–91.3%) | 89.7% (86.2–92.5%) | 0.89 | AI bias documented (Study 54, Mali) |
| HbE carriers | 6 | 91.5% (88.3–94.0%) | 90.8% (87.5–93.5%) | 0.92 | Southeast Asian populations only |
| Compound variants (HbS/C, HbS/β-thal) | 3 | 86.7% (82.1–90.4%) | 88.3% (84.6–91.2%) | 0.87 | Limited data; high bias risk |

**Key Findings:**

β-thalassemia carriers showed the highest AI sensitivity (94.2%), likely due to well-defined quantitative RBC indices (MCV <80 fL, MCH <27 pg) that AI models readily learn.

α-thalassemia carriers had significantly lower pooled sensitivity (89.8%; p = 0.01 vs β-thalassemia), reflecting the diagnostic challenge of normal HbA2 levels requiring genetic confirmation.

Clinically important: The 4.4% sensitivity gap between β-thalassemia and α-thalassemia detection (p = 0.01) suggests that AI models trained primarily on β-thalassemia data may underperform for α-thalassemia carriers, who constitute 40-60% of HHA carriers in Southeast Asian populations.

HbC carriers showed the lowest sensitivity (88.2%), consistent with algorithmic bias documented in our bias audit (File S10), where 30% of models underperformed for African HbS/HbC variants (Δ sensitivity: -8.2%, p = 0.01).

Compound variant detection remains challenging (AUC = 0.87), highlighting the need for training data that includes diverse genotypes.

**Clinical Implication:**

Programs should select AI models calibrated to their predominant local hemoglobinopathy. For regions with mixed HHA types (e.g., Middle East with both β-thalassemia and sickle cell), ensemble models or federated learning across disease categories is recommended.
