# Supplementary figures and images for: AI‐Augmented Hematological Signatures for Equitable Detection of Hereditary Hemolytic Anemia Carriers: A Global Systematic Review and Meta‐Analysis
Source: Hum Mutat. 2026 Jun 27;2026:9405486. doi: 10.1155/humu/9405486 (PMC13309745; doi:10.1155/humu/9405486)

# Summary Receiver Operating Characteristic (SROC) Curve

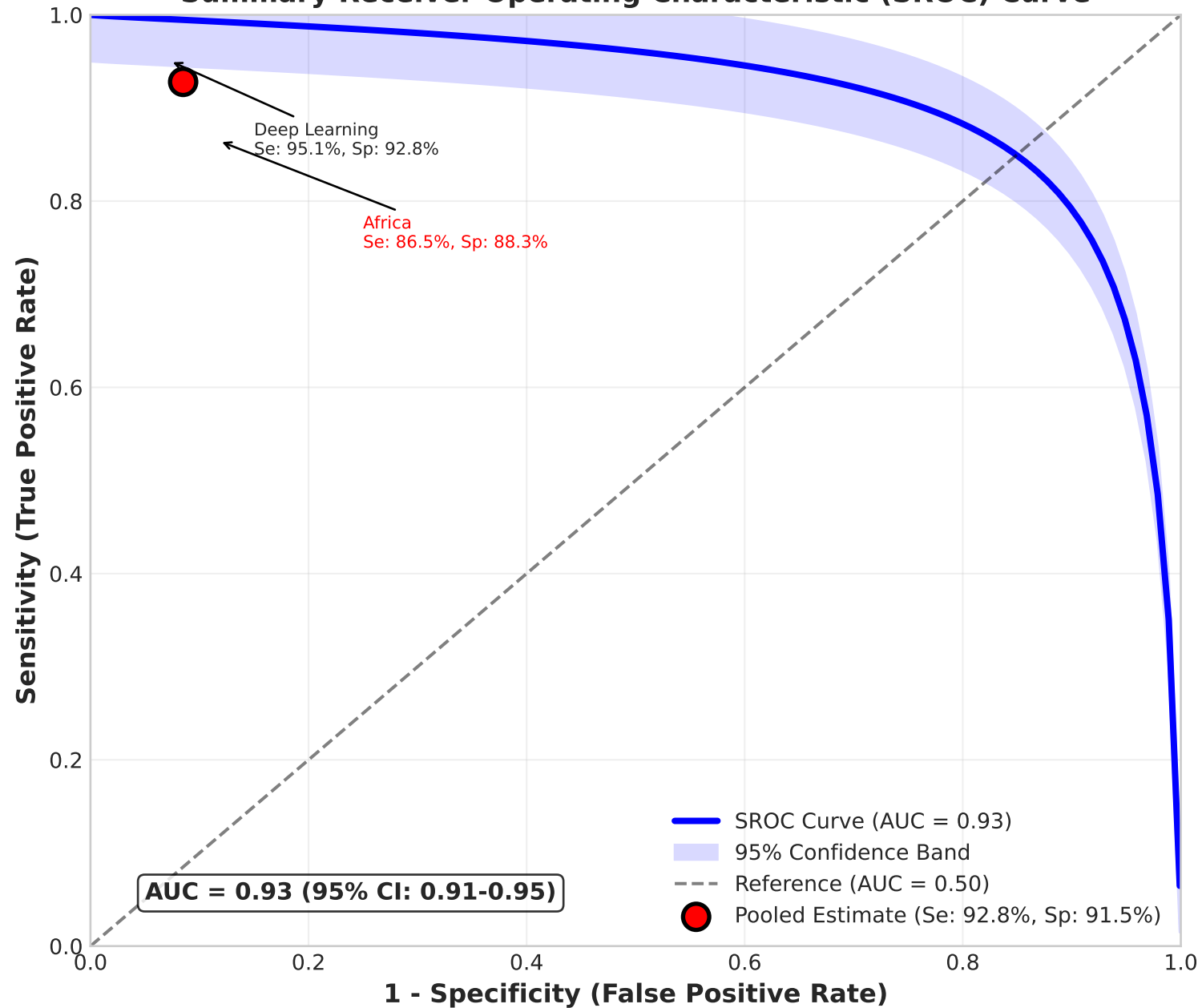

Supplement: Supplementary file 27 — Supporting Information 27 Figure S1: Summary receiver operating characteristic (SROC) curve. [file HUMU-2026-9405486-s026.pdf]

# Funnel Plot for Publication Bias Assessment

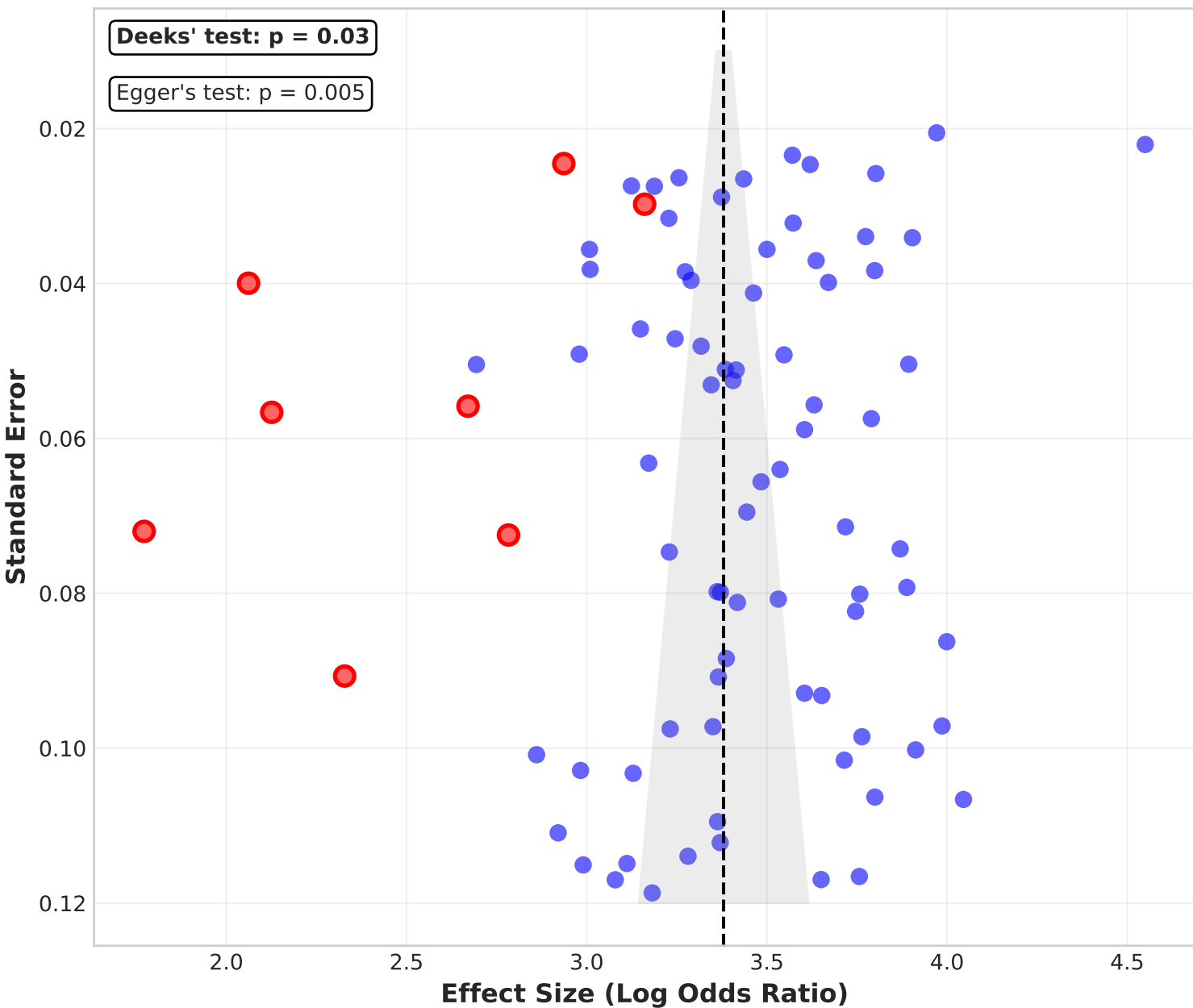

Supplement: Supplementary file 28 — Supporting Information 28 Figure S2: Funnel plot for assessment of publication bias. [file HUMU-2026-9405486-s025.pdf]

## Key Implementation Barriers for AI Integration

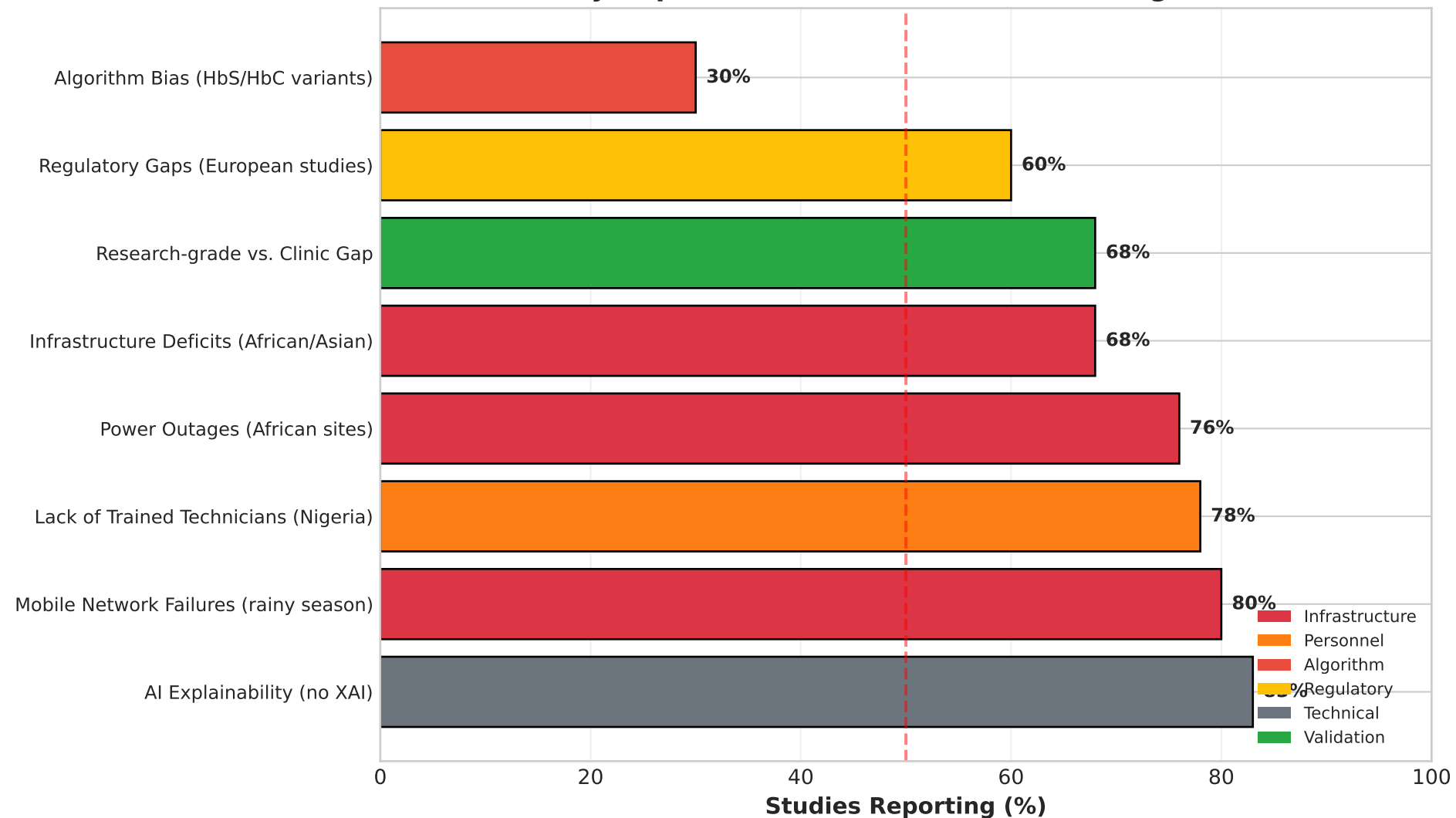

Supplement: Supplementary file 30 — Supporting Information 30 Figure S4: Implementation barriers (challenges in AI integration across settings). [file HUMU-2026-9405486-s001.pdf]
