# Supplementary material for: AI‐Augmented Hematological Signatures for Equitable Detection of Hereditary Hemolytic Anemia Carriers: A Global Systematic Review and Meta‐Analysis
Source: Hum Mutat. 2026 Jun 27;2026:9405486. doi: 10.1155/humu/9405486 (PMC13309745; doi:10.1155/humu/9405486)
Supplement: Supplementary file 29 — Supporting Information 29 Figure S3: Subgroup analysis: sensitivity by region (geographic disparities in AI performance). [file HUMU-2026-9405486-s024.pdf]

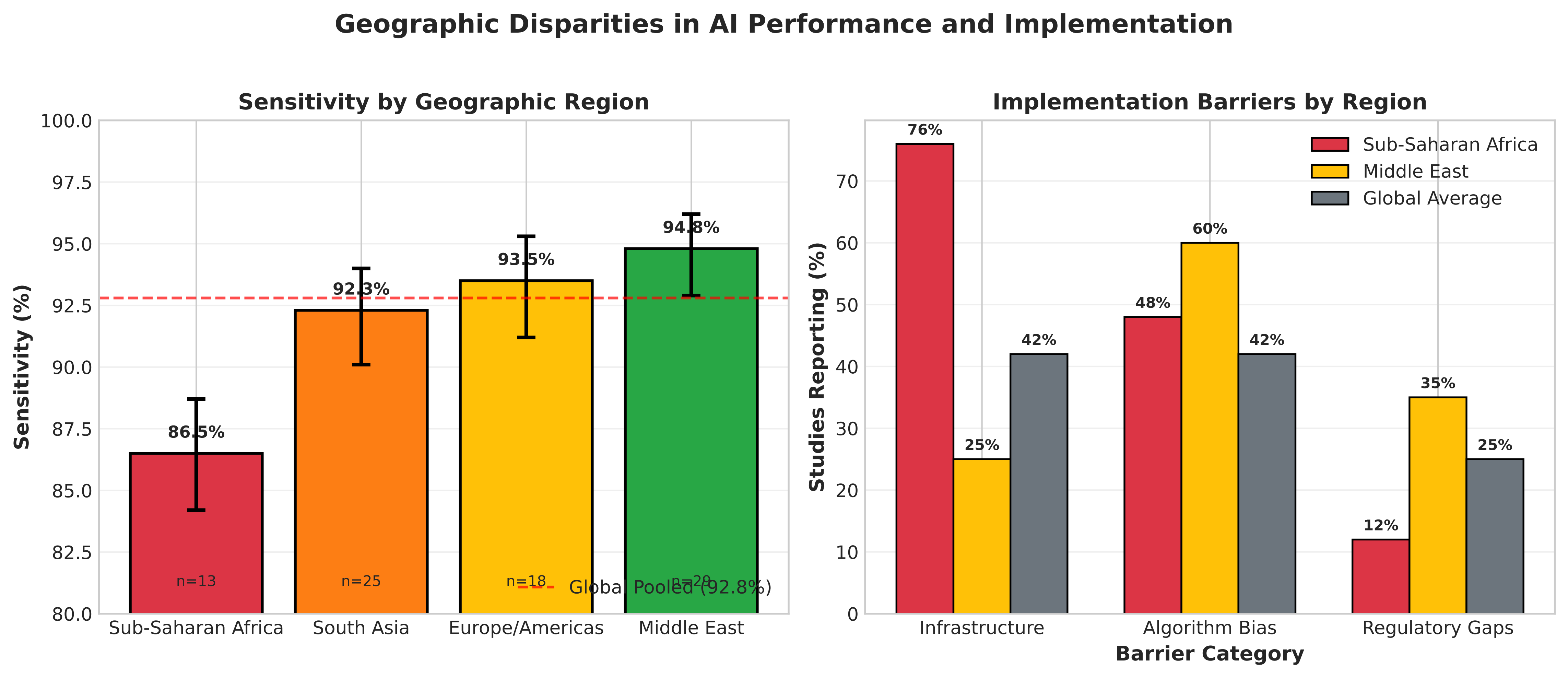
Geographic Disparities in AI Performance and Implementation
Sensitivity by Geographic Region
Sensitivity (%)
86.5%
92.3%
93.5%
94.8%
n=13
n=25
n=18
n=29
Global Pooled (92.8%)
Sub-Saharan Africa
South Asia
Europe/Americas
Middle East
Implementation Barriers by Region
Studies Reporting (%)
Sub-Saharan Africa
Middle East
Global Average
76%
25%
42%
48%
60%
42%
12%
35%
25%
Infrastructure
Algorithm Bias
Regulatory Gaps
Barrier Category
