# Supplementary material for: AI‐Augmented Hematological Signatures for Equitable Detection of Hereditary Hemolytic Anemia Carriers: A Global Systematic Review and Meta‐Analysis
Source: Hum Mutat. 2026 Jun 27;2026:9405486. doi: 10.1155/humu/9405486 (PMC13309745; doi:10.1155/humu/9405486)

Tiered Implementation Framework for AI-Augmented HHA Screening

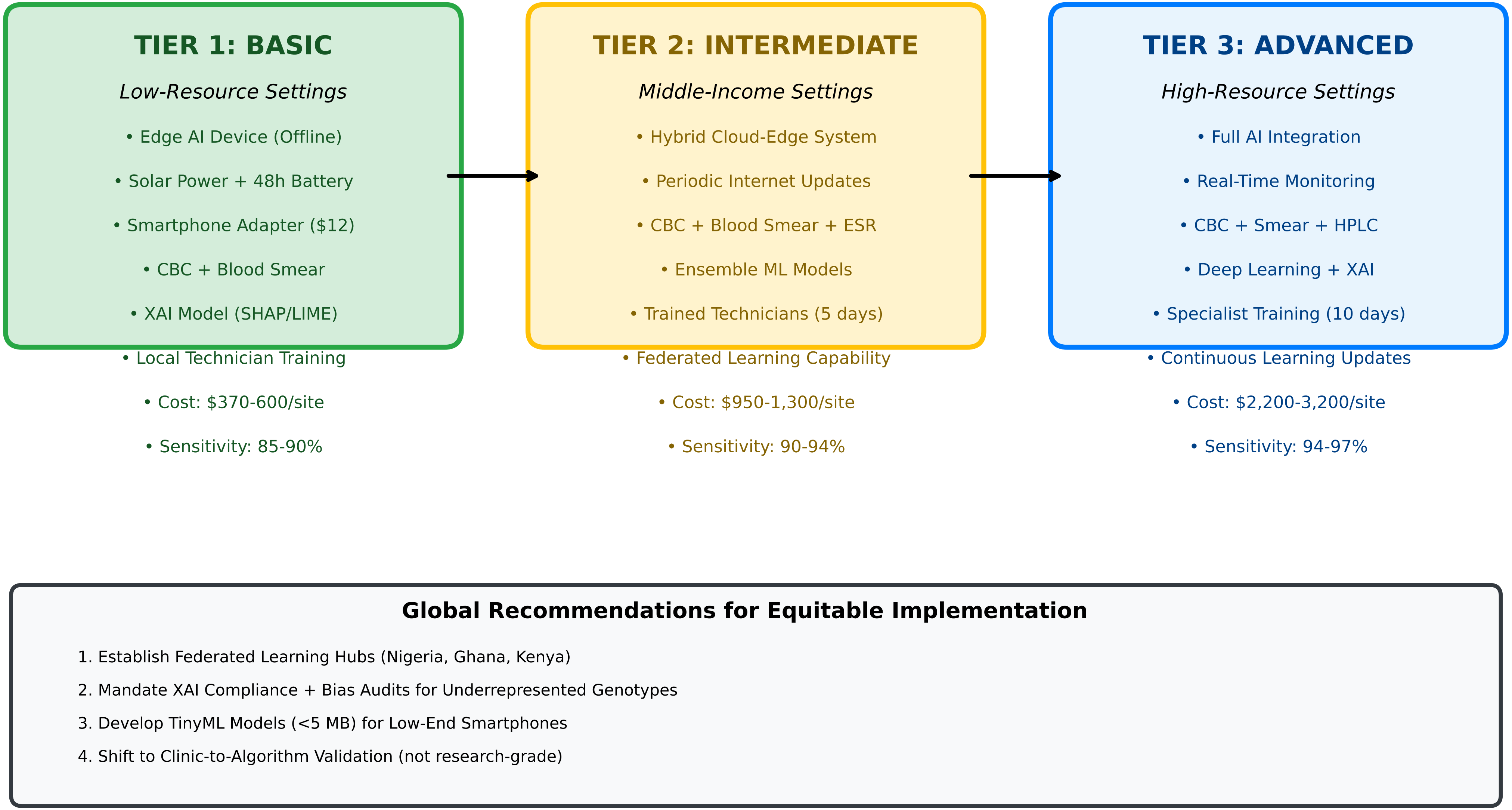

Supplement: Supplementary file 31 — Supporting Information 31 Figure S5: Tiered implementation framework (context‐specific AI integration strategy). [file HUMU-2026-9405486-s002.pdf]
